# A spatially specified systems pharmacology therapy for axonal recovery after injury

**DOI:** 10.3389/fphar.2023.1225759

**Published:** 2023-09-20

**Authors:** Mustafa M. Siddiq, Nicholas P. Johnson, Yana Zorina, Arjun Singh Yadaw, Carlos A. Toro, Jens Hansen, Vera Rabinovich, Sarah M. Gregorich, Yuguang Xiong, Rosa E. Tolentino, Sari S. Hannila, Ehud Kaplan, Robert D. Blitzer, Marie T. Filbin, Christopher P. Cardozo, Christopher L. Passaglia, Ravi Iyengar

**Affiliations:** ^1^ Department of Pharmacological Sciences, Mount Sinai Institute for Systems Biomedicine, Icahn School of Medicine at Mount Sinai, New York, NY, United States; ^2^ Departments of Chemical and Biomedical Engineering, University of South Florida, Tampa, FL, United States; ^3^ National Center for the Medical Consequences of Spinal Cord Injury, James J. Peters VA Medical Center, New York, NY, United States; ^4^ Department of Medicine, Icahn School of Medicine at Mount Sinai, New York, NY, United States; ^5^ Department of Human Anatomy and Cell Science, Basic Medical Sciences Building, Winnipeg, NM, United States; ^6^ Department of Philosophy of Science, Prague and the National Institute of Mental Health, Charles University, Prague, CZ, United States; ^7^ Department of Biological Sciences, Hunter College, City University of New York, New York, NY, United States; ^8^ Department of Rehabilitation Medicine, Icahn School of Medicine at Mount Sinai, New York, NY, United States

**Keywords:** microfluidic chambers, retinal ganglion cell, iDISCO, electrophysiology, electroretinogram

## Abstract

There are no known drugs or drug combinations that promote substantial central nervous system axonal regeneration after injury. We used systems pharmacology approaches to model pathways underlying axonal growth and identify a four-drug combination that regulates multiple subcellular processes in the cell body and axons using the optic nerve crush model in rats. We intravitreally injected agonists HU-210 (cannabinoid receptor-1) and IL-6 (interleukin 6 receptor) to stimulate retinal ganglion cells for axonal growth. We applied, in gel foam at the site of nerve injury, Taxol to stabilize growing microtubules, and activated protein C to clear the debris field since computational models predicted that this drug combination regulating two subcellular processes at the growth cone produces synergistic growth. Physiologically, drug treatment restored or preserved pattern electroretinograms and some of the animals had detectable visual evoked potentials in the brain and behavioral optokinetic responses. Morphology experiments show that the four-drug combination protects axons or promotes axonal regrowth to the optic chiasm and beyond. We conclude that spatially targeted drug treatment is therapeutically relevant and can restore limited functional recovery.

## Introduction

Injury in the adult CNS is often permanent due to inability of severed axons to regenerate. This inability has two major causes: extracellular factors, including myelin-associated molecules, which inhibit axonal outgrowth, (Cajal, 1991; Filbin, 2004; Buchli and Schwab, 2005; Galtrey and Fawcett, 2007; He and Jin, 2016), and lack of appropriate activation of intracellular signaling pathways. Among these are the mTOR and STAT3 pathways (Wu and Bradshaw, 1996; He et al., 2005; Park et al., 2008; Quarta et al., 2014). Simultaneous genome-level activation of these pathways led to robust and sustained axonal regeneration *in vivo* (Sun et al., 2011). Likewise, sustained activation of the mTOR pathway by genetic manipulation, combined with visual stimulation, leads to extended regeneration of optic nerve axons to the brain (Bei et al., 2016; Lim et al., 2016). Other targets of genetic manipulation are the deletion or suppression of the genes for PTEN or SOCS3, which negatively regulate the mTOR and JAK/STAT signaling pathways, respectively. Deletion of PTEN, combined with growth factor-stimulating drugs, also leads to extended optic nerve regeneration and restoration of simple visual behavior (de Lima et al., 2012). A similar study shows optic nerve axons reaching the suprachiasmatic nucleus (Luo et al., 2013). Transcriptomic analyses of dorsal root ganglion neurons following peripheral nerve injury have identified the involvement of numerous signaling pathways including neurotrophins, TGFβ cytokine, and JAK-STAT. Using these transcriptomic data and the Connectivity Map, researchers identified the drug ambroxol, a Na^+^ channel inhibitor that promoted modest axon regeneration after optic nerve crush in mice (Chandran et al., 2016). Despite these extensive studies, no drug or drug combinations have been identified that result in substantial axonal regeneration *leading to restoration of physiological function* have been identified. Initial therapies for severe pathologies often show modest benefits but are none-the-less necessary milestones in developing effective treatments for debilitating diseases. 50 years of investigating antitumor chemicals elapsed before Louis Goodman and Sidney Farber, independent of each other, published landmark advancements in chemotherapy drugs, building on the relentless advancement of the previous half century (Farber and Diamond, 1948; Goodman et al., 1984; Khan et al., 2021). The field of psychiatry has, and continues to, rely on modest effect sizes to treat depression (Khan et al., 2021). The neuroscience community, and the population at large, are so hopeful for a treatment for Alzheimer’s disease that in 2021 the FDA approved aducanumab despite a modest effect on cognitive decline (Ashique et al., 2023). These latter two examples demonstrate the necessity of actionable therapies for diseases with complex and debilitating pathologies, and the hope that the results produced today translate to robust therapies tomorrow.

We have been studying signaling through the G_o/i_ pathway for over 2 decades and found that activated G_αo_ activates STAT3 to promote oncogenic transformation (Ram et al., 2000). Studies of Neuro-2A cells treated with a cannabinoid receptor-1 (CB1R) agonist indicated that this receptor—acting through the GTPases Rap, Ral, and Rac—activates Src and STAT3 to stimulate neurite outgrowth (He et al., 2005; Ram et al., 2000; Jordan et al., 2005). An extensive study identified a complex upstream signaling network that controls multiple transcriptional factors including STAT3 and CREB to regulate neurite outgrowth (Bromberg et al., 2008). This study indicated that several types of receptors regulate STAT3 to drive neurite outgrowth. Likewise, IL-6 promotes neurite outgrowth in a neurite-producing cell line and overcomes myelin-mediated inhibitors to promote neurite outgrowth in primary neurons (Cao et al., 2006; Wu and Bradshaw, 1996). Previously, we showed that submaximal, potentially therapeutic concentrations of the CB1R agonist HU-210 with IL-6 can activate STAT3 in a sustained manner and induce neurite outgrowth in both Neuro2A cells and primary rat cortical neurons in an inhibitory environment *in vitro* (Zorina et al., 2010). Initial reasoning indicates that IL-6 might be deleterious as it is proinflammatory. However, at appropriately low concentrations its beneficial effects could be resolved from its proinflammatory effects.

**FIGURE 1:**
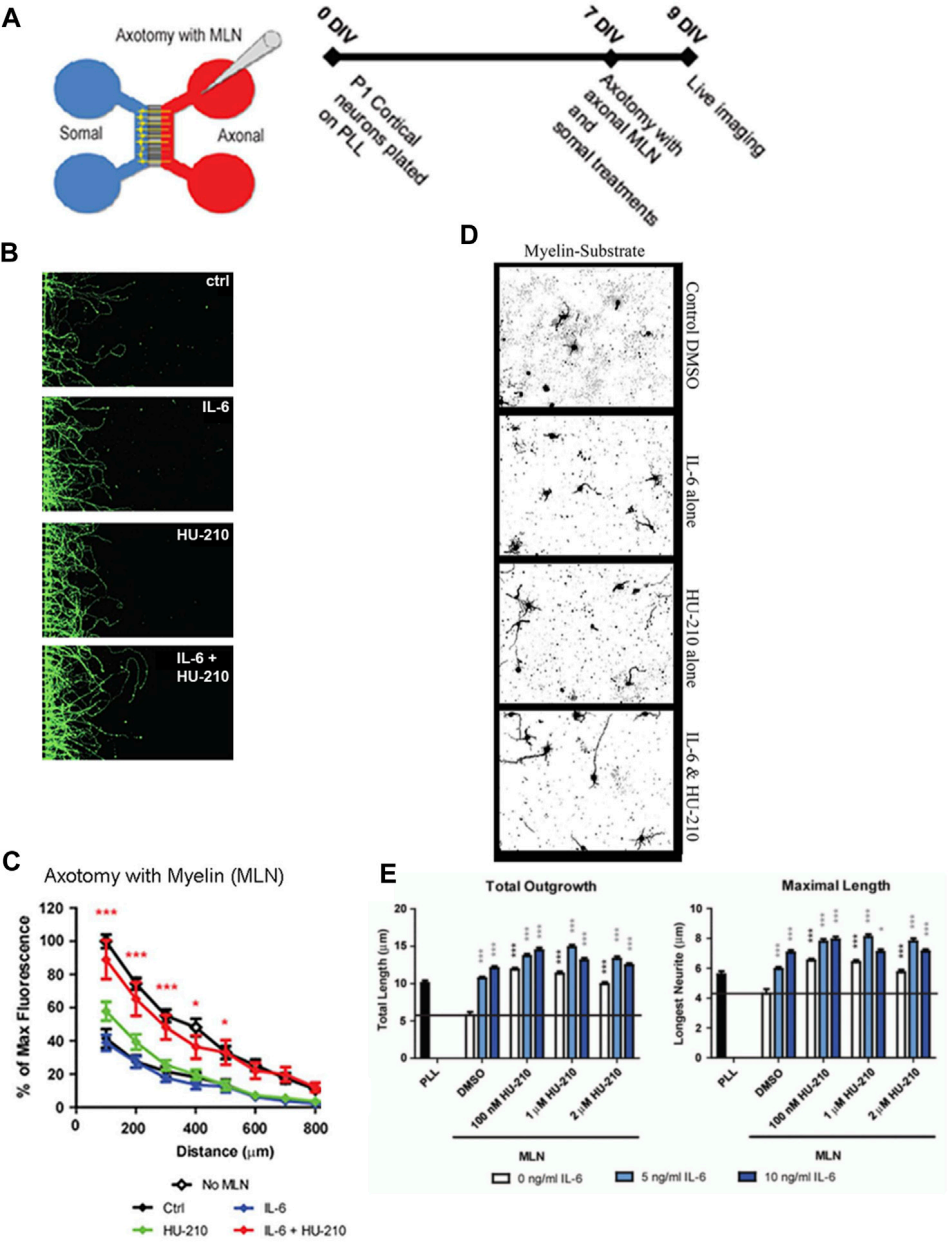
Somal treatment with IL-6 and HU-210 promotes neurite outgrowth on myelin. **(A)** Schematic representation of the microfluidic chamber. **(B)** Neurons were cultured in chambers on PLL for 7 days *in vitro* (DIV), and axotomy was performed. The axonal compartment was then filled with 20 μg/mL solution of myelin (MLN). 10 ng/mL IL-6 and/or 200 nM HU-210 treatments were applied to the somal compartment of each respective chamber. The neurons were imaged live after 48 h using Calcein AM. Representative confocal images of neurons following axotomy and treatment. **(C)** Neurite outgrowth was quantified by measuring the total fluorescence at multiple cross-sections of the image. The zero point on the x-axis represents the right edge of microgrooves. All data points were normalized to the first point of “No MLN” as 100 percent. Statistical differences were calculated from four independent experiments using two-way ANOVA. Asterisks show significance compared to Ctrl treatment at a given distance; *, *p* < 0.05; ***, *p* < 0.001. **(D)** P1 cortical neurons were plated on myelin–coated slides and treated with IL-6 and/or HU-210 over a range of concentrations. The neurons were fixed after 24 h and labeled for β-III-tubulin. Representative images of neurons plated on myelin (MLN) substrate treated from top to bottom, with either DMSO, IL-6, HU-210 or IL-6&HU-210 labeled with β-III-tubulin show treatment with IL-6 and HU-210 together promotes neurite outgrowth in an inhibitory environment in a dose-dependent manner. **(E)** Total outgrowth of neurites and neurons’ longest neurite were quantified using two-way ANOVA. Asterisks show significance compared to DMSO alone (black bar); *, *p* < 0.05; ***, *p* < 0.001.

We anticipated that stimulating signaling and transcription in the cell body of injured neurons to enable growth in inhibitory environments alone would not lead to long-distance axon regeneration to the brain. Numerous studies have shown that both transport of vesicles to the axon tip to deliver new membrane (VV) and growth of microtubules in the axons (VV) are required for neurite extension and axon growth. We hypothesized that we would not only need to stimulate biosynthetic pathways in the cell body, but also modulate these cell biological processes for effective axonal growth. Based on this hypothesis we sought to develop a systems-level combination therapy based on spatial specification of drug action at the cell body and at the axons the site of injury. We used our *in vitro* experiments to identify a 2-drug combination that act on cell surface receptors at low concentrations could stimulate biosynthetic processes in the injured neuron *in vivo*. We sought to combine this with additional drugs that could regulate cell biological processes in the axon. To test if our hypothesis regarding the different drug groups and actions are likely to be correct, we used computational models of neurite outgrowth, which serves as a surrogate for axonal growth. We identified drugs that could selectively modulate subcellular function in the cell body and axon (Alushin et al., 2014). We then utilized this spatial information to develop a multi-drug combination with the potential for treating CNS nerve injury by promoting long-range axonal regeneration *in vivo*. Since transcriptional regulation is critical for neurite outgrowth and for axonal regeneration, (Cao et al., 2006), we reasoned that stimulation of CB1R and IL-6Rs occurs in the cell body. Experimental evidence *in vitro* supports our reasoning (Yadaw et al., 2019). We then looked for potential drugsthat target cell biological processes in the axon. We utilized computational dynamical models of neurite outgrowth to predict how different subcellular processes could promote axonal regeneration and how these processes could be modulated by drugs (Alushin et al., 2014) Based on these simulations, we decided that our focus on microtubule growth, which is required for axon regeneration, is likely to be correct. Microtubules are stabilized by Taxol by reducing the intrinsic GTPase turnover rate of tubulin (Hellal et al., 2011). Increasing conversion of dynamic to stable microtubules increases axonal length to promote regeneration (Shim et al., 2005). Additionally, axonal growth requires the incorporation of membrane vesicles at the growing tip, which is inhibited by cell remnants at the site of injury because cell debris binds the growth cone membrane receptors of the same family that regulate fusion of membrane vesicles (Chen et al., 2001; Mosnier et al., 2007). Since computational modeling showed that regulation of vesicle fusion dynamics is an important regulator of neurite length, treating the site of injury to clear it of inhibitory agents and reduce the inflammatory response could contribute to axonal regeneration. Based on these considerations, we chose a protease that could act locally to reduce the debris field and inhibit inflammation. We selected Activated Protein C (APC), which is a serine protease endogenous to the coagulation system that is anti-inflammatory and promotes stem cell activity in neuronal tissue in mice (Siddiq et al., 2015; Wang et al., 2016). Thus, using a combination of computational modeling and experimental evidence we identified a spatially specified four-drug combination for axonal regeneration. The overall logic for this four-drug combination is schematically shown in Figure 1. We tested if a combination of these drugs, two applied at the cell body and two at the axon at the site of injury, promoted long distance regeneration such that visual stimulation led to restoration of physiological function.

## Materials and methods

### Care and use of animals statement

These experiments were conducted under the guidance of the Institutional Animal Care and Use Committee established in the Icahn School of Medicine at Mount Sinai and in accordance with federal regulations and the guidelines contained in the National Research Council Guide for the Care and Use of Laboratory Animals. To replace the use of animals, when possible, rigorous computational models and *in vitro* experiments were employed prior to *in vivo* experiments. Likewise, the optic nerve crush model was chosen to deliver robust and reproducible results while minimizing the distress animals experience during these experiments. Adult Sprague-Dawley or Long Evans rats (250–280 g, 8–10 weeks old), as well as postnatal day 1 Sprague-Dawley rats, were used in these experiments. Animals were housed in individually ventilated cages under the supervision of the Icahn School of Medicine Center for Comparative Medicine and Surgery and kept on a 12 h light/dark cycle. During surgeries, deep anesthesia was induced using 2.5%–3.0% isoflurane (Piramal Pharma Solutions, Sellersville, PA), and post-surgical pain was managed with 0.1 mg/kg slow-release buprenophine HCL (Par Pharmaceutical, Woodcliff Lake, NJ).

### Rat primary cortical neuron cultures

Rat primary cortical cultures were dissected from postnatal day 1 Sprague Dawley rat brains. Cortices were incubated twice for 30 min with 0.5 mg/mL papain in plain Neurobasal (NB) media (Invitrogen, Waltham, MA) with DNase (Sigma, St. Louis, MO). Cell suspensions were layered on an Optiprep density gradient (Sigma) and centrifuged at 1900 *g* for 15 min at room temperature (22°C). The purified neurons were then collected at 1,000 x *g* for 5 min and counted.

Suspensions of purified CNS myelin (1–2 μg/well) were plated in microfluidic chamber slides and desiccated overnight to create a substrate of myelin on eight well chamber slides as previously described (Mukhopadhyay et al., 1994; Tang et al., 2016). Primary cortical neurons were diluted to 35,000 cells/mL in NB supplemented with B27, L-glutamine, and antibiotics (Thermo Fisher Scientific, Waltham, MA). 300 μL of the cell suspension was seeded to each well and incubated for 24 h. The microfluidic chambers allow to quantify the outgrowth, we immunostained the neurites using Calcein AM (ThermoFisher Scientific, Waltham, MA). Outgrowth was quantified by measuring the total fluorescence at multiple cross-sections from the right of the microgrooves. The zero point on the x-axis represents the right edge of microgrooves. All data points were normalized to the first point of “No MLN” (see Supplementary Materials). 7 days *in vitro* (DIV), axonal injury was modeled by axotomy. The media was aspirated from the axonal chambers with the tip of the aspirator held against the microgrooves. This was repeated three times per chamber. Fresh supplemented NB media containing 10 ng/mL IL-6, 200 nM HU-210, or a combination of both, was supplied to the chambers and live imaging was conducted the following day. Neuronal survival was quantified by treating the somal compartment of microfluidic chambers with Calcein AM (green, alive) and ethidium homodimer-1 (red, dead) for 48 h following axotomy. The ratio of living to dead cells was calculated by measuring the number of pixels of each color.

Primary cortical neurons were seeded on myelin-coated slides and treated with IL-6 and/or HU-210 at a range of concentrations and were fixed after 24 h. Total outgrowth and the longest neurite were calculated using the AutoNeuriteJ macro in ImageJ (Eric Denarier) and compared to DMSO alone.

For Western Blots, primary neurons were treated with IL-6, HU-210, or both and lysed in radioimmunoprecipitation assay (RIPA) buffer (Cell Signaling Technology, Danvers, MA) supplemented with protease and phosphatase inhibitors (Thermo Fisher Scientific) over ice. After determination of concentration with a standard protein assay kit (Bio-Rad, Hercules, CA), the cell lysates were subjected to immunoblot analysis using standard procedures and visualized by enhanced chemiluminescences (ECL). Polyclonal rabbit antibodies directed against phosphor-STAT3 and total STAT3 were from Cell Signaling Technology Inc.

### Computational model

We employed a top-down model previously described that predicts the necessary parameters for given neurite outgrowth velocities, which were estimated from the literature (Alushin et al., 2014). The model assumes that, for a given growth velocity, the *production of membrane components, vesicle transport and exocytosis,* and *microtubule growth* should be balanced at steady state. The classical model describes kinesin-mediated anterograde and dynein-mediated retrograde transport of membrane vesicles between the Trans Golgi Network (TGN) and the growth cone plasma membrane (GC-PM) through three intermediate compartments—the cell body cytoplasm, neurite shaft cytoplasm, and growth cone cytoplasm—where the binding of vesicles to the GC-PM results in a neurite growing longer. In each compartment, kinesin has a different affinity for microtubules, leading to varying fractions of vesicles that are transported to the growth cone, and in physiological conditions, leads to a reservoir of membrane vesicles near the growth cone. In this model, we determined the size of the reservoir under various drug treatment conditions, and the size of the reservoir was indicative of the capacity of axonal growth in response to treatment with different drugs. Simultaneously, microtubule growth is simulated by stable microtubules and alternating phases of growth and catastrophic breakdown of dynamic microtubules.

Here, we divide the neurite shaft cytoplasm into a proximal and distal compartment to account for the action of multiple drugs. Since HU-210 and IL-6 stimulate transcription, we have assumed that this stimulation leads to sufficient levels of biosynthesis of molecules needed for axon regeneration at all times. Based on this assumption, the levels of all components are buffered in the model when the cell body is stimulated by HU-210 and IL-6.

**FIGURE 2:**
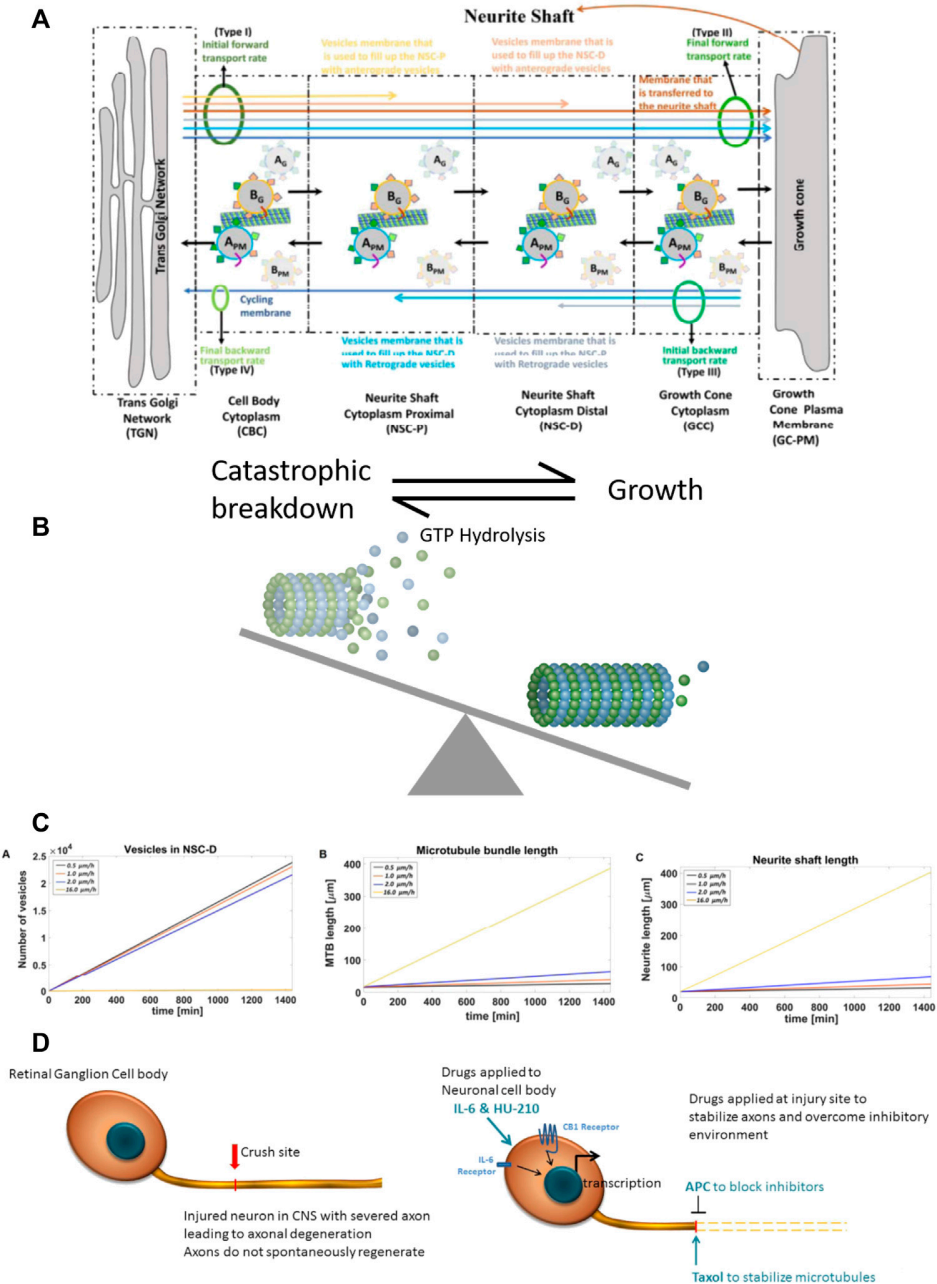
Computational model of neurite outgrowth. **(A)** Schematic representation of the model used to generate an analytical solution for predicting kinetic parameters of neurite outgrowth. The model distinguishes four different membrane types (I-IV, green ovals) from TGN to the growth cone. Initial (I) and final (II) anterograde membrane transport rates refer to budding and fusion rates at the TGN and GC, respectively. Initial (III) and final (IV) retrograde membrane transport rates represent endocytosis and fusion rates at the GC and TGN, respectively. Budded vesicles at the TGN can be distinguished by their destination: the yellow arrow represents anterograde vesicle loss in the NSC-P compartment; light brown arrow, anterograde vesicle loss in the NSC-D; brown arrow, fusion with the GC membrane and addition to the neurite shaft; light gray arrow, retrograde vesicle loss in the NSC-D; light blue arrow, retrograde vesicle loss in the NSC-P; and dark blue arrow, recycling membrane from the GC-PM to the TGN. Anterograde moving vesicle B_G_ moves forward while anterograde moving vesicle A_G_ back fuses with the TGN because coat protein A SNAREs have a higher affinity with TGN SNAREs. Likewise, endocytosed vesicle B_PM_ back fuses with the GC-PM because coat protein B SNAREs have a higher affinity for GC SNAREs. **(B)** Dynamic microtubules have a stochastic profile, which changes as they undergo catastrophic disassembly by hydrolysis of GTP bound tubulins at the tip. Taxol present at the injury site reduces GTP hydrolysis, leading to elongation of the dynamic microtubule bundle and conversion to stable microtubules. Here, the balance between catastrophic breakdown and growth is shifted towards growth by the presence of Taxol. **(C)** The calculated values for the vesicle levels in the NSC-D compartment under various drug conditions and corresponding rates of microtubule elongation and neurite shaft length. The profile of the plots indicate that the synergistic effects of the four-drug combination can be explained parsimoniously by the action of APC, which, by removing the extracellular inhibitory environment, allows for vesicle movement from the NSC-D to the GC-PM. Enhanced microtubule elongation due to Taxol-dependent stabilization of dynamic microtubules allows for the observed overall growth rate of the axon. **(D)** Cartoon schematic for spatial systems reason for combinational therapy to promote CNS axonal regeneration. Drugs are applied to both the injury site (APC and Taxol) and directly to the RGC cell bodies (IL-6 and HU-210), indicating a spatially resolved approach to applying drugs is required to promote robust axonal regeneration.

Experimentally-observed velocities of axonal growth for different drugs and drug combinations were used as a constraint for our model to calculate required membrane fusion at the GC-PM. The model constraints specified the rate of back-transported membrane from the growth cone to the TGN and the fraction of moving and stationary vesicles in the various compartments. We assumed the endocytosed vesicles should transport 0.5 μm membrane/min back to the TGN based on experiments in the literature (Alushin et al., 2014). We calculated other parameters of the model analytically for a given neurite velocity. Velocities of growth were obtained from preliminary *in vivo* experiments, where we graphed the Intensity of Fluorescent signal of Cholera Toxin B (CTB) over distance (μm) from the crush site and measured average growth with the indicated combinations or single drug over a 3-week period. We then divided the total average length of growth by 21 days and determined the average velocities. See Figure 2A for the distribution of all membrane proteins, membrane vesicles, and fluxes in the terminal and intermediate compartments at steady state used in the model.

### Optokinetic responses

Awake rats were placed on an elevated platform in a virtual reality chamber composed of four computer monitors facing inward (OptoMotry system, Cerebral Mechanics Inc, Lethbridge, AB, Canada). A virtual cylinder covered with a vertical sine wave grating was presented to the rat and a video camera situated above the animal provided real-time feedback to the observer. The spatial frequency of the sine was maintained by manually repositioning the center of the cylinder at the animal’s head. The sine wave drifted clockwise or counterclockwise and the observer judged whether the rat tracked the grating with slow movements of its head and body, with the direction of the stimulus alternating randomly between trials. Spatial frequencies were increased if tracking was observed and decreased if no tracking was observed. Visual thresholds were obtained using a staircase method in which spatial frequency steps were halved after a reversal and terminated after 10 reversals. One staircase was performed in each direction as each direction elicited an independent response from each eye, with the clockwise direction corresponding to the left eye and the counterclockwise direction corresponding to the right eye. The observer was blind to the direction of the stimulus, as well as the spatial frequency and the number of reversals.

### Electrophysiological recordings

All electrophysiology was performed using Long Evans (pigmented) rats. Flash ERG (fERG) and visual evoked potential (VEP) recordings were performed at the University of South Florida (Ertürk et al., 2012). Rats were anesthetized with intraperitoneal ketamine (75 mg/kg) and xylazine (5 mg/kg) and placed on a heating pad. Cannulas were surgically inserted into the femoral vein for intravenous drug delivery and the trachea for mechanical ventilation if necessary. Anesthesia was maintained with intravenous infusion (all dosages are mg/kg/hr) of ketamine (50), xylazine (1.5), dextrose (600), and physiological saline. Heart rate was monitored with ECG electrodes placed in the trunk of the animal, and body temperature was monitored with a rectal thermometer. The eyes were covered with clear contact lenses, and pupils were dilated with mydriatic eye drops (cyclopentolate hydrochloride, Sandoz, Basel, Switzerland). Flash stimuli were produced by a green LED encased in an opaque tube the blocked the escape of light and the tube exit was fitted with a hemispherical diffuser that covered the eye to provide Ganzfeld illumination. fERGs were recorded with a ring-shaped gold electrode placed on the cornea. Platinum needle electrodes were inserted in the temples and tail to serve as reference and ground, respectively. VEPs were recorded from both hemispheres via two 1.3 mm steel screws inserted through the skull 7 mm posterior to Bregma and ± 2.5 mm lateral to the midline. Recorded signals were differentially amplified (10,000X) and filtered (0.1—1,000 Hz) by a multichannel bioamplifier and digitized at 1 kHz. Animals were dark-adapted for 15 min prior to data collection, and then baseline fERG and bilateral VEP data were collected simultaneously for a series of 100 brief (10 ms) flashes with an interstimulus interval of 3 s to allow for recovery of visual sensitivity. Average responses were measured for a flash series of increasing light intensity in seven logarithmic steps to a maximum of 1.32 log candelas/m^2^/s (cd·s/m^2^, 100% condition) presented to one eye and then the other. Since approximately 90% of the fibers in the rat optic nerve cross to the opposite side to innervate the brain, when we flashed light and recorded from the right injured optic nerve, we recorded the corresponding VEP on the left side of the brain. All procedures were approved by the IACUCs of the Icahn School of Medicine at Mount Sinai and University of South Florida at Tampa in accordance with the NIH guidelines and AAALAC accreditation.

### Rat optic nerve regeneration *in vivo*

Adult male Sprague-Dawley rats were anesthetized with 2.5%–3.0% isoflurane and placed in a stereotaxic frame. The right optic nerve was exposed and crushed with fine forceps for 10 s. For drug treatment, animals received a single 2.5 μL intravitreal (intraocular) injection of either 0.5% DMSO or a combination of IL-6 (5 μg/mL) and HU-210 (300 μM) (see Table 1) immediately after the crush. In later experiments, animals also received gelfoam soaked in 0.5% DMSO, APC (4.1 mg/mL, Haematologic Technologies, Essex, VT), and/or Taxol (5 μM) placed over the injury site. Three days later, we confirmed that lens injury was avoided and injected for a second time intravitreally 2.5 μL of DMSO or IL-6 (5 μg/mL) and HU-210 (300 μM). Three days prior to sacrificing the animals we labelled the regenerating axons with 5 μL of 1 mg/mL cholera toxin B (CTB, List Biological Lab, Campbell, CA) coupled to Alexa-488 (Invitrogen), which we intravitreally injected. Alternatively, regenerating axons were labelled by injecting 0.5 μL of high titer (>10^12^ genome copies per ml) AAV8-GFP intravitreally 2 weeks prior to sacrifice. On day twenty-one post-crush, animals were anesthetized with ketamine (100 mg/kg) and xylazine (20 mg/kg) injected intraperitoneally, and the brain was fixed by transcardial perfusion with cold 4% PFA in PBS (pH 7.4). The optic nerves and chiasm attached were dissected out and post-fixed in 4% PFA overnight at 4°C, switched to 30% sucrose in PBS until the nerves sank to the bottom of the tube (1–2 days), and rinsed for 1 hour in ice-cold PBS, and then prepared for chemical clearing. Since the advent of the 3DISCO clearing techniques, (Mehta et al., 2007), we are no longer dependent on sectioning the tissue; this method also eliminates bias associated with artifacts produced by sectioning. The whole nerve was exposed to a graded series of dehydrations with tetrahydrofurane (THF; Sigma) diluted in water, final concentrations THF: 50%, 70%, 80%, 100%, and 100% again. The nerves were incubated in each solution for 20 min at room temperature on an orbital shaker, and then incubated in the clearing agent, dibenzyl ether (DBE; Sigma) overnight at room temperature on the orbital shaker. Microscope slides were mounted with Fastwell chambers (Electron Microscopy Sciences, Hatfield, PA) and the cleared sample was placed on the slide and covered with DBE and a No. 1.5 micro coverglass. We imaged the whole sample on an Olympus Multiphoton microscope with a 25X water immersion lens. Optic nerve immunostaining was alternatively conducted using an anti-GAP-43 antibody (Novus Biologicals, Centennial, CO) coupled to Alexa-488 (Invitrogen)

**TABLE 1:** Summary of visual evoked potentials (VEP).

Treatment	Number of animals tested	Number of animals with VEP in response to light
No treatment	7	0
4-Drug Combination	19	5

For quantification of axonal regeneration in our ONC samples, multiple images of each sample were taken under multiphoton microscopy and combined in photomontages using Adobe Photoshop (Adobe, New York, NY). We used ImageJ software (NIH) to measure the axonal outgrowth determined by immunolabeling in our regenerated nerves in photomontages (Mukhopadhyay et al., 1994). Pixel thresholding was performed to identify labeled axons, and we measured in 250 micron increments from the crush site. We graphed the Intensity of Fluorescent signal (area occupied) over distance (μm) from the crush site using GraphPad Prism Software (GraphPad, San Diego, CA).

### Statistical analysis

Except where noted, analyses were performed using GraphPad Prism software (GraphPad, San Diego, CA), and data are represented as mean ± SEM. Statistical significance was assessed using paired one-tailed Student’s *t* tests to compare two groups, and one-way ANOVAs with Bonferroni’s *post hoc* tests to compare among three or more groups.

To evaluate the effectiveness of the four-drug treatment in restoring the VEP following injury, we analyzed traces from each animal to detect physiological responses that exceeded RMS noise by a Z-score of at least three. We set this as a criterion to reliably eliminate false positives, as we could not distinguish two signal-free (RMS noise) traces at a Z-score of 3. RMS noise was calculated for each time point using the detrended traces recorded at the three lowest stimulus intensities (0.001%, 0.01%, and 0.1%), since none of the injured nerves showed apparent VEPs at these intensities. For each animal, the mean of these designated noise traces was normalized to 0 mV. We then measured the peak amplitudes of the responses to stimulation at 10%, 50%, and 100%, and these were converted to Z-scores against the mean noise at the corresponding time point. The presence of a VEP was defined by Z-score > 3. Chi-square analysis was used to compare the number of treated and untreated animals that showed VEPs in response to both the 10% and 100% stimuli.

## Results

### IL-6 and HU-210 together stimulates neurite outgrowth in an inhibitory environment

We previously found that submaximal concentrations of IL-6 and HU-210 in combination have an additive effect on neurite outgrowth for rat cortical neurons in primary culture (Yadaw et al., 2019). We tested if this effect could be observed for regeneration of growing neurites that had been severed *in vitro* and grown in an inhibitory environment. For these experiments, we used microfluidic chambers with 450 μm long microgrooves, where the cell bodies could be compartmentalized from the growing axons (Figure 1A). We plated primary rat cortical neurons in these chambers and allowed them to grow long neurites. To mimic axotomy, we lesioned all the neurites in the chambers on the right side and then added 200 nM HU-210 and 10 ng/mL IL-6 to the somal chamber (Figure 1B) with the addition of 20 μg/mL myelin (MLN), a non-permissive substrate used to inhibit axonal growth. Neurite outgrowth was quantified as fluorescent intensity relative to controls. Fluorescent intensity of each treatment and control were calculated in 100 μm sections starting from the edge of the microgrooves. We observed that addition of the combination of IL-6 and HU-210 to the cell bodies promoted longer growth of axotomized processes in the presence of MLN, compared to either treatment alone (Figure 1C). The myelin substrate here strongly inhibits outgrowth, and neurites are expected to be very short. Controls and treatments with IL-6 and HU-210 in the absence of MLN (“No MLN”) are shown in Supplementary Figure S1.

As we had previously shown for Neuro2A cells, the combination of IL-6 and HU-210 in primary cortical neurons stimulated STAT3 by phosphorylation at Tyr705 (Supplementary Figure S2), consistent with our earlier observation that HU-210-stimulated neurite outgrowth depends on Stat3-mediated transcription (Ertürk et al., 2012). We confirmed the presence of the receptors for IL-6 (gp130) and CB1 (CB1R) in our neuronal cultures by RT-PCR, as shown in Supplementary Figure S3-I. We also detected the receptors by immunolabeling and found both gp130 and CB1R were expressed in the somal compartment of microfluidic chambers (Supplementary Figure S3-II). Axotomy significantly increased the number of dead somata *in vitro*, while IL-6 and the combination of IL-6 and HU-210 alleviated cell death (Supplementary Figure S3-III). These experiments indicate that HU-210 + IL6 applied at the cell body stimulate biosynthetic processes that promoted neuroprotection and enabled neurite outgrowth.

To further test this hypothesis, we measured neurite outgrowth as well as the length of the longest axon of primary cortical neurons plated on a myelin substrate (Figure 1D). Cortical neurons were treated with DMSO, a range of concentrations of IL-6 (5—10 ng), and/or a range of concentrations of HU-210 (100 nM—2 µM). Each concentration of IL-6, each concentration of HU-210, and the combination of the drugs at any concentration resulted in more neurite outgrowth as well as longer neurites compared to DMSO (*p* < 0.05). Furthermore, the combination of IL-6 and HU-210 consistently produced significantly more total outgrowth and maximal growth than IL-6 or HU-210 alone (*p* < 0.05, Figure 1E).

### Neurite outgrowth is due to drug-drug synergy

Prior computational models showed that the combination of stabilizing growing microtubules and enabling fusion of membrane vesicles at the growth cone under conditions of continuous biosynthesis promoted synergistic growth of axons (Alushin et al., 2014). Thus, we focused on external inhibitors of axonal growth, including myelin-associated proteins such as Nogo. Biochemical experiments have shown that Nogo receptors and gangliosides inhibit neurite outgrowth. Binding of extracellular agents such as Nogo to gangliosides can promote endocytosis, (Dent et al., 1999; Mimura et al., 2006; Fewou et al., 2014), thus disrupting delivery of new membrane vesicles to the growth cone plasma membrane (GC-PM) that is essential for continued growth of the axon. Since the site of injury contains many such myelin-related proteins, to relieve inhibition we decided to test the application of a protease that could act locally. For this we chose Activated Protein C (APC), which has been reported to promote neuronal repair and axonal regeneration (Siddiq et al., 2015; Wang et al., 2016). APC inhibits inflammatory activity of endothelial cells near the injury site, which can increase intracellular vesicle mobility from a vesicle reservoir to the GC-PM. Taxol is known to stabilize dynamic microtubules, leading to elongation of stable microtubules that form the growing neurite scaffold, and thus promotes axonal regeneration (Hellal et al., 2011). We used gel foam to apply 5 μM Taxol or 4.1 mg/kg APC to the site of injury, either alone or in combination with HU-210 (300 μM) and IL-6 (5 mg/mL) applied to the cell body through two intravitreal injection.

Experimentally, HU-210 and IL-6 applied at the cell body allow neurite outgrowth with a velocity of 0.5 μm/h. When Taxol was applied alongside HU-210 and IL-6, neurites grew at 1 μm/h. Growth of the microtubule bundle was inhibited by external debris, as growing microtubules loop back on themselves and fragment when they encounter the growth cone membrane (Leon et al., 2000). When APC was applied alongside HU-210 and IL-6, neurites grew at 2 μm/h, as vesicles could move from the vesicle reservoir to the growth cone cytoplasm and fuse with the GC-PM (0.6052 μm^2^/min, calculated analytically). However, there was insufficient microtubule elongation for the neurite to grow faster.

Analytical solutions (Supplementary Equations S1–S158) for the model identified how the four-drug combination could allow axons to grow with a velocity of 16 μm/h. These calculations indicate that the synergistic effects if the four-drug combination can be explained parsimoniously by the action of combined action of APC and Taxol, depleting of the membrane vesicle pool in the distal neurite shaft cytoplasm, allowing for the expansion of the GC-PM, which in turn enables the microtubules to be stabilized and elongate without inhibition.

**FIGURE 3:**
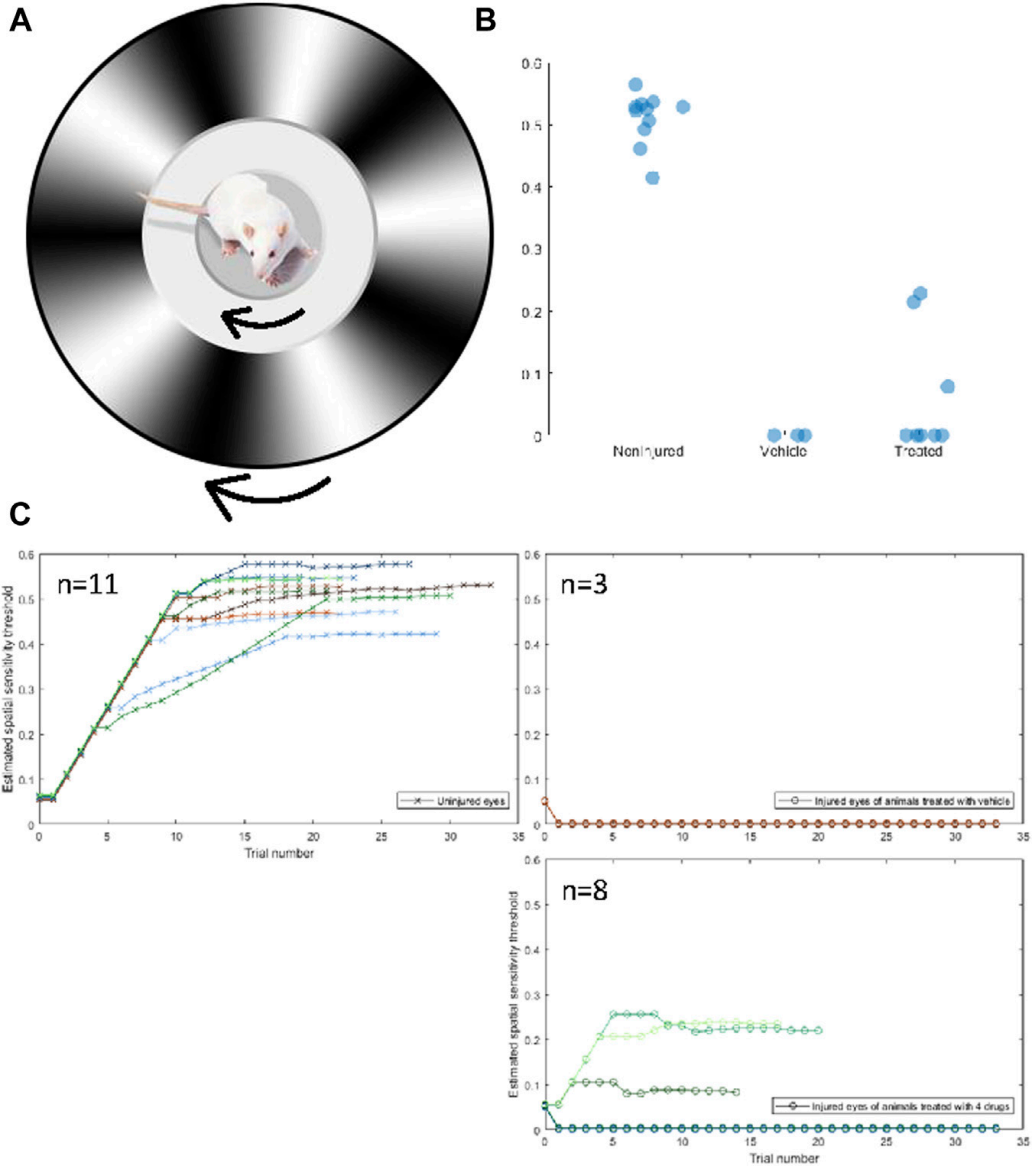
Four-drug combination partially restores visual function compared to injured optic nerves treated with vehicle only. **(A)** The Optokinetic response is measured by placing a rat on an elevated platform and presented a virtual environment showing a drifting sinusoidal wave with specific spatial and temporal frequencies. The rat unconsciously tracks the drifting pattern, with clockwise tracking dependent on the left (uninjured) eye and counterclockwise tracking dependent on the right (injured) eye. The spatial frequency of the drifting pattern increases with consecutive identifiable tracking and decreases if no tracking is observed. The spatial sensitivity threshold is the maximum spatial frequency at which tracking is observed. **(B)** The uninjured eyes of rats had a mean (±std) spatial sensitivity threshold of 0.506 ± 0.042 cpd. The threshold of injured eyes of treated rats was 0.065 ± 0.100 cpd, and the threshold of injured eyes of nontreated rats was 0 ± 0 cpd. Three out of eight treated rats displayed a threshold above 0 cpd in their injured eye, whereas none of the nontreated rats displayed a threshold above 0 cpd. A one-sided independent *t*-test was conducted to determine if the thresholds of treated rats were significantly different from untreated rats. The difference was not significant (*p* = 0.544), but the distribution of thresholds of treated rats was determined to be significantly more than 0 with a variance equal to that of the uninjured eye (z = 4.391†). Both groups are significantly lower than the noninjured eye (*p* < 0.05*) **(C)** Spatial frequency as a function of trials for the uninjured (CW, crosses) and injured (CCW, circles) of untreated rats (left) and treated rats (right).

Figure 2 illustrates the computational model. We assumed the initial forward transport rate at the TGN was constant because of the activity of HU-210 and IL-6 increase the capacity of membrane production at the TGN. To achieve a specific axonal outgrowth velocity, we calculated the fraction of bound kinesin-mediated membrane vesicle transport from the reservoir (NSC-D) to the Growth Cone Cytoplasm (GCC). Membrane that is incorporated into vesicles that bud from the TGN can be separated into 4 different membrane vesicle types (I, II, III, IV). Type-I vesicles are added to the growing distal neurite shaft cytoplasm (NSC-D) as part of a reservoir which can be mobilized on sudden demand for membrane at the growth cone. Type-II vesicles are added to the GC-PM and from there the growing neurite shaft. Type-III and type-IV vesicles are also added to the GC-PM, but the fused membrane will then be incorporated into endocytic retrograde vesicles and sent back to the TGN. This constitutes the cycling of membrane between the TGN and GC-PM. As a simplification, we assume in our solution that membranes transported in an anterograde manner is only transported as vesicles that bud from the TGN with coat protein B and retrogradely transported membranes are vesicles that bud from the GC-PM with coat protein A. Overall, our model indicates that relief of inhibition of GC-PM expansion is sufficient to account for the observed experimental growth rates. From the simulations in Figure 2 and the experiments in Figure 1 we developed the overall logic for four-drug combination is schematically shown in Figure 3.

### The four-drug combination treatment restores function in animals subjected to optic nerve crush

Our primary goal in developing therapies is to restore neurological function following injury. Thus, we developed an *in vivo* optic nerve crush model, in which the somas of retinal ganglion cells were accessible through the vitreous of the eye and the axons of retinal ganglion cells were accessed through an incision behind the eye. The optic nerve was exposed and crushed with fine forceps. The conceptual layout is described in Figure 2D. Briefly, we tested whether the combination of IL-6 (5 mg/mL) and HU-210 (300 μM) at the cell body and APC (4.1 mg/mL) and Taxol (5 μM) at the injury site led to restoration of visual function after optic nerve crush. Regeneration of retinal ganglion cells has been previously documented (Douglas et al., 2005). The rat’s optic nerve is over 6 mm from the retina to the optic chiasm, thus, any regeneration to the chiasm—and beyond to the superior colliculus—would represent a significant achievement over our previous *in vitro* experiments.

Visual function was assessed in 11 animals using the optokinetic response (OKR). The OKR is a reflexive movement of the head and eyes that results from visual stimulation of the accessory optic system, which is independent of the primary visual pathway (Simpson, 1984; Creel, 2019). The OKR of each eye can be quantified individually, allowing for the assessment of the crushed optic nerve in comparison to the non-injured nerve. Using the OKR, we examined the spatial sensitivity threshold. Animals were placed on an elevated platform surrounded by computer monitors on all sides. The computer monitors were used to produce a virtual environment displaying a drifting sinusoidal gradient that rotated 360° around the cursor, which was placed on the animal’s head when viewed from above. The gradient rotated clockwise to produce the OKR of the left, uninjured eye, and counterclockwise to produce the OKR of the injured eye (Figure 3A). An observer, blind to the direction of the stimulus and the treatment of the animal, determined if the OKR occurred. The observer verified that the OKR occurred at least three times before advancing to the next trial. The spatial frequency increased as the OKR was observed on consecutive trials until the threshold was reached. Uninjured eyes had a spatial sensitivity threshold of 0.5 ± 0.05 cpd, there was no difference between treated and untreated animals, which passed a Shapiro-Wilks test of normality. Variance can be explained by differences in animal visual acuity due to age, attentiveness to the stimulus, tolerance of being handled, etc. The thresholds of uninjured (n = 11), non-treated (n = 3), and treated (n = 8) eyes are displayed in Figure 3B, and the spatial frequency as a function of trial for each animal is displayed in Figure 3C. Three animals that were treated with the four-drug combination displayed a moderate spatial sensitivity threshold from their injured eye. Without the four-drug treatment, no injured, untreated animal showed an OKR from their injured eye. The mere fact that some animals were able to respond to a visual stimulus presented to their injured eye is incredible. That these animals were able to reliably respond to the stimulus suggests a relatively robust signal is being transmitted from the retinal to the accessory optic system. While the threshold of both groups of injured eyes was significantly lower than that of the uninjured eyes, the distribution of thresholds of treated animals was significantly different from that of untreated animals, assuming the variance of the uninjured eyes (z = 4.391). This indicates that the 4-drug combination had a regenerative or protective, albeit moderate, effect on crushed optic nerves. A representative video of an animal performing the OKR is shown in the Supplementary Materials. A response in the injured eye upon drug treatment can be observed at 34 s.

**FIGURE 4:**
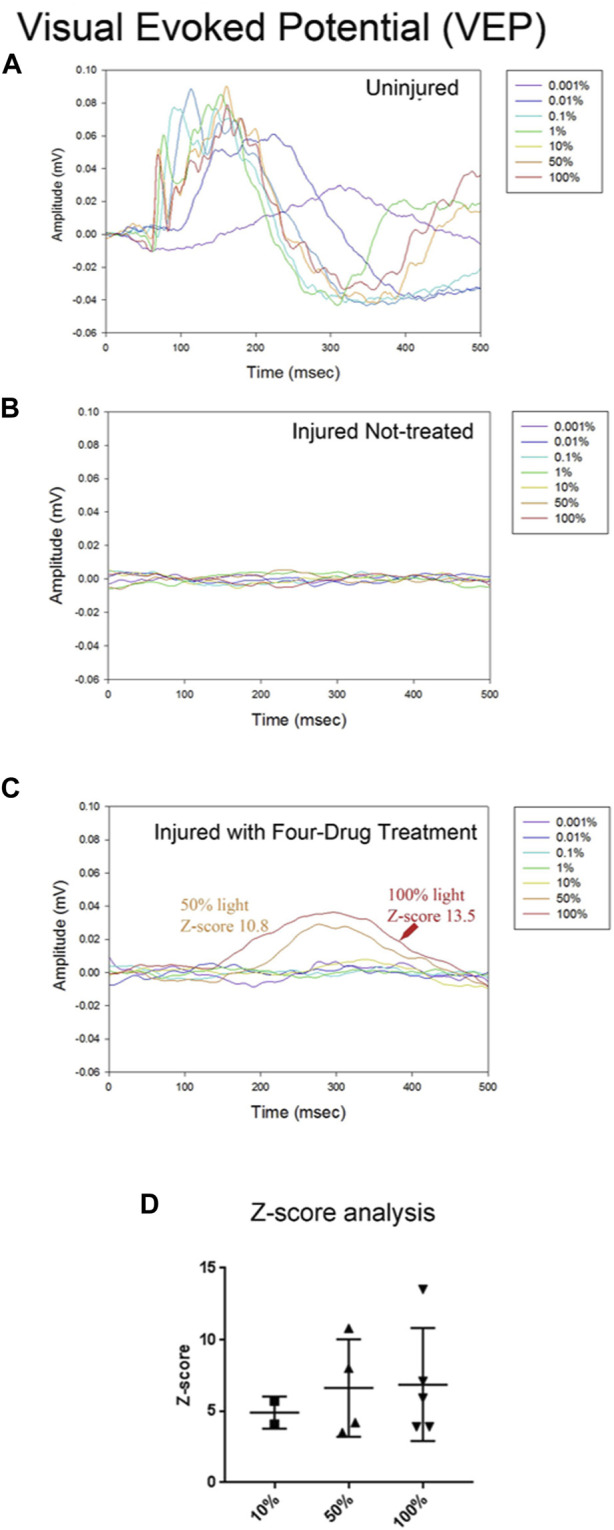
The four-drug combination enhances VEP responses compared to injured optic nerves treated with vehicle only. Visual evoked potential (VEPs) from the brain were simultaneously recorded with the fERGs. In the uninjured eye **(A)** the VEP was slow for the dimmest flash, peaking around 300 ms, and had fast onset with multiple wave components and compound action potentials for the brightest flashes. Injured eyes with no treatment **(B)** have no detectable VEP activity. The VEP in several treated animals **(C)** exhibited a slow waveform in response to the highest intensities of light (50% and 100%), suggesting that some axons regenerated all the way to the brain. Z-score values for 50% and 100% are shown in the figure, indicating they are significantly different from the noise. **(D)** Summary of the Z-scores calculated for the five animals with four-drug treatment that showed VEP signals at some intensity. Of the five animals tested at 10%, 2/5 met the criterion to be above a Z-score of 3; all 4 tested at 50%, showed a response; and all 5 tested at 100% showed a response. The one animal not tested at 50% showed VEPs at 10% and 100%.

Having seen some recovery of visual function with the OKR, we investigated the function of the visual pathway directly. To do this, we stimulated each eye with a series of light flashes at different intensities (0.001%–100% of 1800 cd/m^2^/s) and simultaneously recorded the flash ERG (fERG)- which is indicative of activity in all neuronal cells of the retina (not just the RGCs that populate the optic nerve)- and the visual evoked potential (VEP) detected bilaterally from the primary visual cortex (Renier et al., 2014). In Figure 4A, we show the VEP for light flashes presented to the non-injured eye. The VEP is slow and monotonic at lower intensities (<0.1%) and becomes markedly faster and multiphasic at higher intensities. The VEP is completely absent for flashes to the injured eye of vehicle-treated animals (Figure 4B). In contrast, VEPs were reproducibly detected at high light intensities in several animals that were treated with the four-drug combination (Figure 4C). At high intensities, the signal was slow and monotonic in these animals, like non-injured eyes at low light intensities. Z-score analysis was performed on each VEP to determine if potentials rose above the levels of noise (further details in Methods section). We found that responses for all the vehicle-treated animals (*n* = 7) were not significantly different from noise, even at the highest flash intensities. However, 5 of 19 drug-treated animals showed VEP responses above the cut-off criterion at different flash intensities (Figure 4D; Table 1), indicating partial restoration of functional connectivity in those animals. The major target of healthy RGC axons is the dorsal lateral geniculate nucleus, which projects axons to the primary visual cortex. Another target of RGC axons is the subcortical accessory optic system. Even a few RGC axons synapsing onto subcortical or geniculate bodies could produce the functional and physiological activity observed in this study. This data excludes any nerves with regenerating axons that have yet to synapse onto cerebral targets. Data of VEP recordings for three individual animals for the non-injured eye, injured eye with vehicle only (DMSO), and injured eye with four-drug treatment are shown in Supplementary Figures S4–S6.

Visual information reaches the brain, albeit incompletely. We recorded the fERG to determine if the loss of retinal neurons accounted for the partial or complete loss of visual function in injured eyes. Supplementary Figure S7 shows representative fERGs for light flashes to the uninjured eye, the injured eye of a vehicle-treated animal, and the injured eye of a drug-treated animal. While fERG signals appeared altered and often reduced at all intensities for injured eyes with vehicle treatment relative to the uninjured control side, which is consistent with neurodegeneration that occurs due to the crush, there were no statistical differences between the injured eyes of treated and nontreated animals. All animals retained an a-wave, b-wave, and oscillatory potentials. These results indicate that the animals did not experience retinal ischemia or generalized retinal dysfunction, and that the reduction in visually-induced activity in the brain, and visually-induced behavior, is a product of the loss of connection between the retina and the lateral geniculate nucleus. That is, the loss of retinal ganglion cell axons. Likewise, we conclude that the recovery in cortical activity and behavior is a product of axonal regeneration.

We also quantified the activity of RGC axons between the crush site and the optic chiasm with the Compound Action Potential (CAP), which represents the global activity of cells in the region of interest. The CAPs from injured optic nerves of animals both treated with the four-drug combination and given the vehicle were too noisy to differentiate (data not shown).

### Four drug combination treatment results in morphological regeneration of crushed axons *in vivo*

The administration of the four-drug combination led to moderate functional recovery, as we had predicted such an effect from the mechanistic computational model. These observations imply axonal regeneration at the cellular level. Therefore, we examined the morphology of the optic nerve. In a different group of animals from those from whom functional experiments were conducted, we sacrificed animals after 3 weeks of drug or vehicle treatment. In a subset of studies, we intravitreally injected AAV8-GFP or CTB coupled to Alexa fluor 488 1 week prior to euthanasia to visualize the regenerated axons. The whole nerve was chemically cleared using the 3-DISCO technique. In separate studies, we immunostained the whole nerve with GAP-43 and chemically cleared them using the iDISCO technique (Strittmatter et al., 1994; Fischer et al., 2017). Both CTB- and GFP-labeled nerves were imaged on a multiphoton microscope.

**FIGURE 5:**
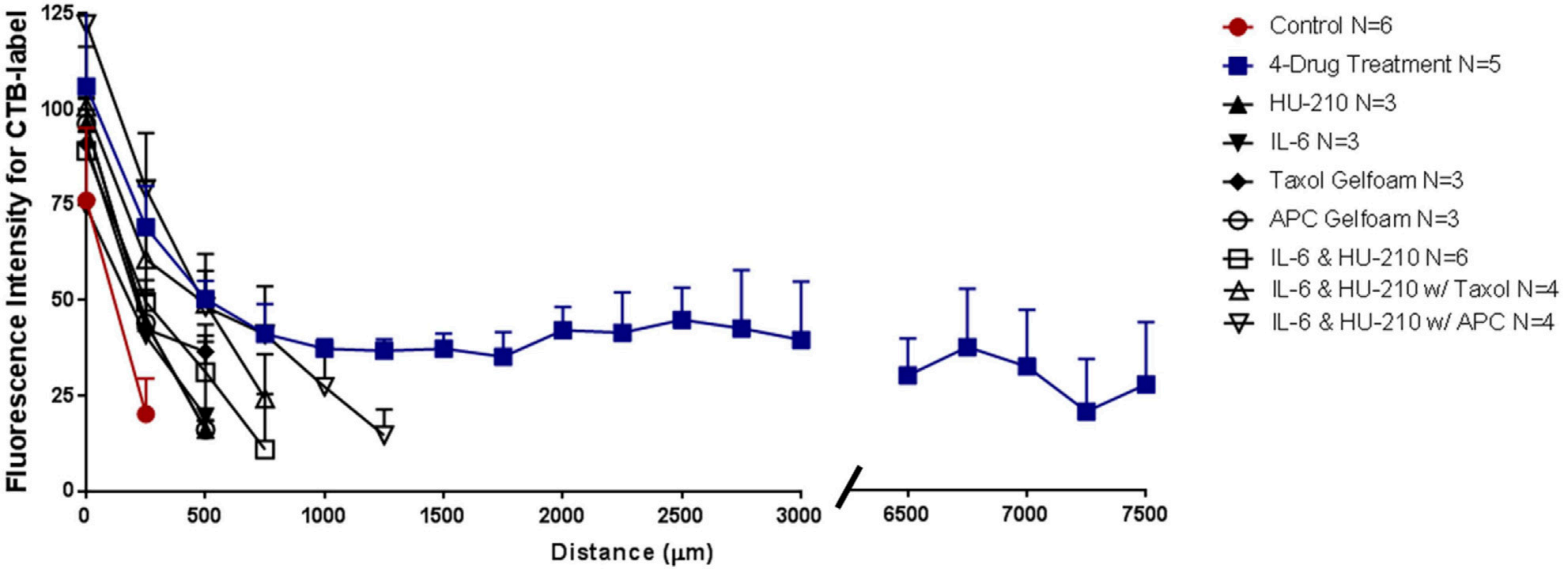
Four-drug combination in the rat ONC model promotes axonal regeneration to the chiasm. Length of axons in the injured nerve were quantified by the fluorescent intensity of the CTB label for animals treated with vehicle, individual drugs, IL-6 and HU-210 combined, both combined with Taxol or combined with APC, or all four drugs together. CTB labelling showed that long axons only grew in the presence of all four drugs. Representative images of CTB-labeled axons are found in Figures 6, 7.

With our four-drug combination, we observed that seven out twenty-nine animals tested (5 with CTB-labeling and 2 with GFP immunostaining) had regenerating axons that could be detected at the crush site and continuing to the chiasm, at 7.5 mm from the crush site (Table 2). Fluorescence of the CTB label was quantified at 250-µm intervals from the crush site, revealing a striking difference between the treated and untreated groups (Figure 5, see Figures 8–10 for representative images of each condition). Moderate regeneration was observed with the combination of IL-6 and HU-210, slightly more with the addition of Taxol or APC, but no other combination of drugs approached the regeneration produced by the four-drug combination. Fluorescence was detected at least 3.0 mm from the crush site, well into the optic chiasm, in eleven out of twenty-nine animals treated with the four drugs, compared to just over 1 mm with the combination of IL-6, HU-210, and APC; which produced the second greatest amount of regeneration. One-way ANOVA found a difference among the six groups, *p* < 0.0001. Tukey pairwise comparisons between-group differences conserving the overall Type one error at 0.05, i.e., comparing the four-drug treatment to the four other groups measured (control no treatment, APC alone, Taxol alone and IL-6 and HU-210) and concluded that four drug treatment was significant from each other group by *p* < 0.0001. Data are represented as mean ± STDEV for optic nerve regeneration.

**TABLE 2:** Summary of optic nerve regeneration.

Treatment	Number of animals tested	Regenerating axons not detected past crush site	Regenerating axons at least 3 mm from crush site	Regenerating axons to the chiasm (nearly 8 mm from crush site)
No treatment	11	11	0	0
4-Drug Combination	29	18	11	7

Note Table 1 and Table 2 are two separate groups of animals.

**FIGURE 6:**
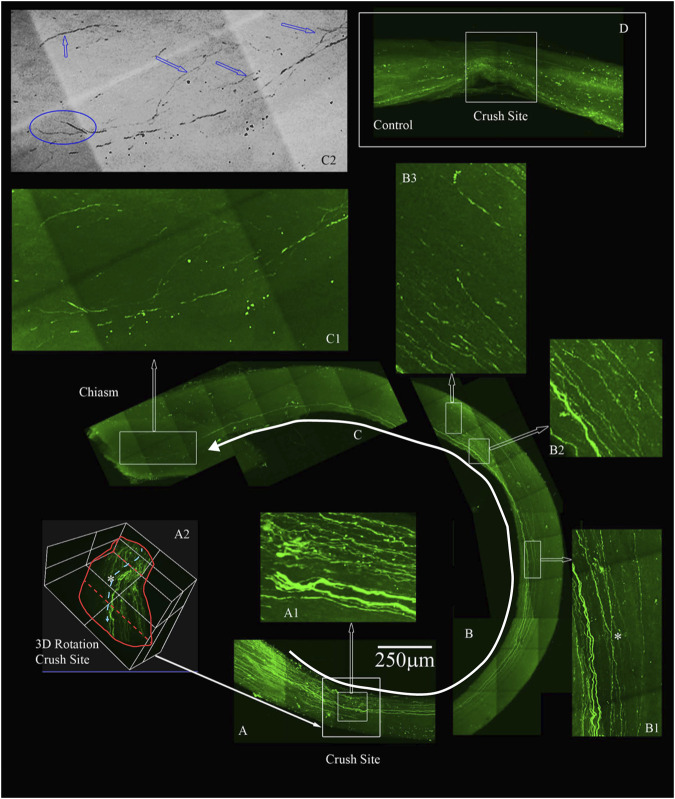
Four-drug treatment promotes robust axonal regeneration in the rat ONC model as detected by AAV8-GFP labeling. We labeled the regenerating axons with AAV8-GFP injected intra-vitreally to the RGC cell bodies 2 weeks prior to euthanizing. The optic nerves are immunolabeled by iDISCO with anti-GFP and then chemically cleared. **(A)** Crush site of the nerve of displaying regenerating fibers expressing GFP. The inner box is magnified in **A1**, displaying tortured growing axons in the center of the nerve (inner box with traced white arrow). **A2**, is a 3D rendering of the nerve at the crush site (outer white box in A shown by filled white arrow) that is rotated on its axis to emphasize regenerating axons are not growing on the edges but more in the center of the nerve (see asterisk). **(B)** Continuation of the nerve displaying GFP expressing axons that are growing in the center of the nerve and even crossing over in **B1** (see asterisk). **B2** and **B3** are magnified regions with GFP labeled axons over 4 mm away from the crush site. **(C)** We detect GFP labeled axons growing into the chiasm of the nerve and the end of the chiasm, we have a magnified region in **C1**. The image in C1 was converted to grayscale and then inverted in **C2**, to emphasize the tortured growth into the chiasm as demarcated by the blue arrows and the bifurcation (blue circle in **C2**) of the axons growing into the chiasm. **(D)** An example of an adult rat that only received vehicle controls. No GFP labeled axons are displayed past the crush site. All images are taken on an Olympus Multiphoton microscope with a 25X water immersion lens. The images in **A1**, **B1**-3 and **C1** are taken at 25x with a 1.5x Zoom. Micrometer of 250 microns is shown in **(A)**.

To confirm that the morphological growth we were seeing was not dependent on a particular method of labelling the regenerating axons, we used GFP labels Animals transfected with GFP were sacrificed 3 weeks after optic nerve crush and axons were labeled with anti-GFP antibody. iDISCO was performed to detect GFP label in regenerating axons in the optic nerve of animals with the four-drug treatment. We show a robust response to our four-drug treatment starting at the crush site in Figure 6A. We observed tortured growth at the crush site (Figure 6A1), and by preparing a 3D image of the crush site (highlighted by asterisk in Figure 6A2) (Davies et al., 1999; Steward et al., 2008). The nerve is outlined in red with axons growing from the superior to the inferior plane, indicating displaced axons regenerated following optic nerve crush. These observations indicate that the axons are not spared fibers. The growth continues along the optic nerve into Figure 6B, where we point out GFP labeled axons crossing one another (highlighted by asterisk in Figure 6B1) and displaying tortured, discontinuous growth in the center of the nerve (Figure 6B2, B3). We detected growth into the chiasm, as shown in Figure 6C. In the magnified area shown in Figure 6C1, we see torturous axonal growth, including possible bifurcating axons, which is better displayed in the inverted image in Figure 6C2. Blue arrows indicate tortured growing axons, and the blue oval indicates the bifurcation, hallmarks of regenerating fibers (Fischer et al., 2017). In total, three out of eleven animals labeled with GFP show long, regenerating axons at least 3 mm past the crush site.

**FIGURE 7:**
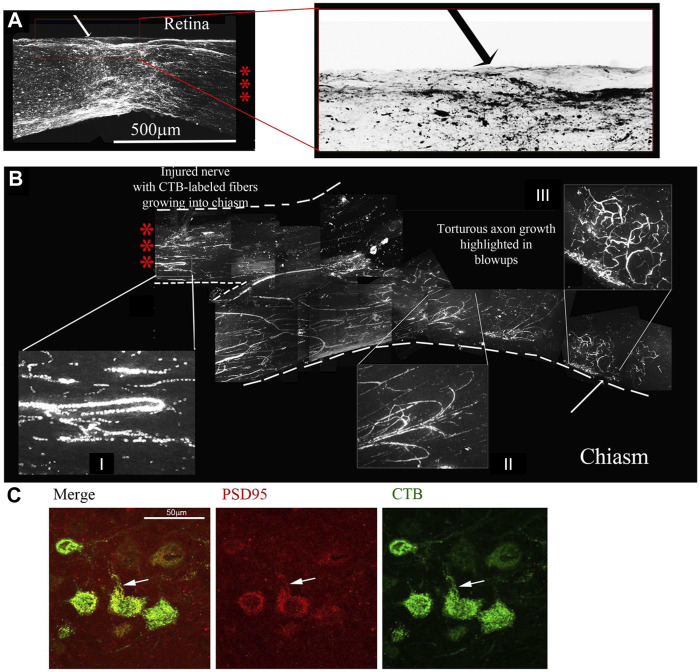
Four-drug treatment promotes robust axonal regeneration in the rat ONC model as detected by CTB-labelling. CTB labelling revealed a torturous pattern of growth from the site of the optic nerve crush **(A)** to the chiasm **(B)** of the same animal 7 days post CTB labelling. The three red asterisks between the retina with the crush site **(A)** to the chiasm **(B)** represent the same nerve without 6 mm of the nerve between, from 0.5 mm from the crush site to the chiasm. The red box is enlarged of the crush site in A and inverted to reveal the crushed regenerating axons. I and II display random processes and bifurcation of axons, a result of growth without guidance cues. III displays torturous axon growth further into the optic chiasm. **(C)** Regenerating axons reach the brain. Brain sections were immunostained for CTB, revealing CTB labeled neurons and fibers in the superior colliculus. CTB (green) and PSD95 (red) colocalize (yellow) in neurons in the superior colliculus, as highlighted by the white arrows.

Figure 7 displays an example of extensive regeneration of CTB-labeled fibers in a treated animal. A close-up view of the crush site with inverted image colors shows that our crushes are complete with no observable sparing (Figure 7B, the right panel shows a magnification and color inversion of the area in the red box for clarity). The axons we observe at the crush site and in the chiasm displayed hallmarks of regenerating fibers (Figure 7C). Magnifications I and II show random processes and bifurcation of axons due to growth without guidance cues. Magnification III shows more torturous axon growth. Furthermore, we see CTB-labeled axons in the center of the injured nerve and not just at one edge of the nerve, which would be indicative of spared fibers. Additional examples of the torturous pattern of axonal regeneration are found in Supplementary Figure S8.

Given the regrowth of the severed axons up to the optic chiasm and the physiological and behavioral effects of the four-drug treatment, we decided to look for regeneration into the brain. We sectioned the contralateral brain and observed with immunostaining for CTB that there was label in the superior colliculus, an area innervated by the optic nerve. We also looked for the synaptic marker PSD95 and found it co-localizing with the CTB, indicating the presence of new synapses (Figure 7D).

**FIGURE 8:**
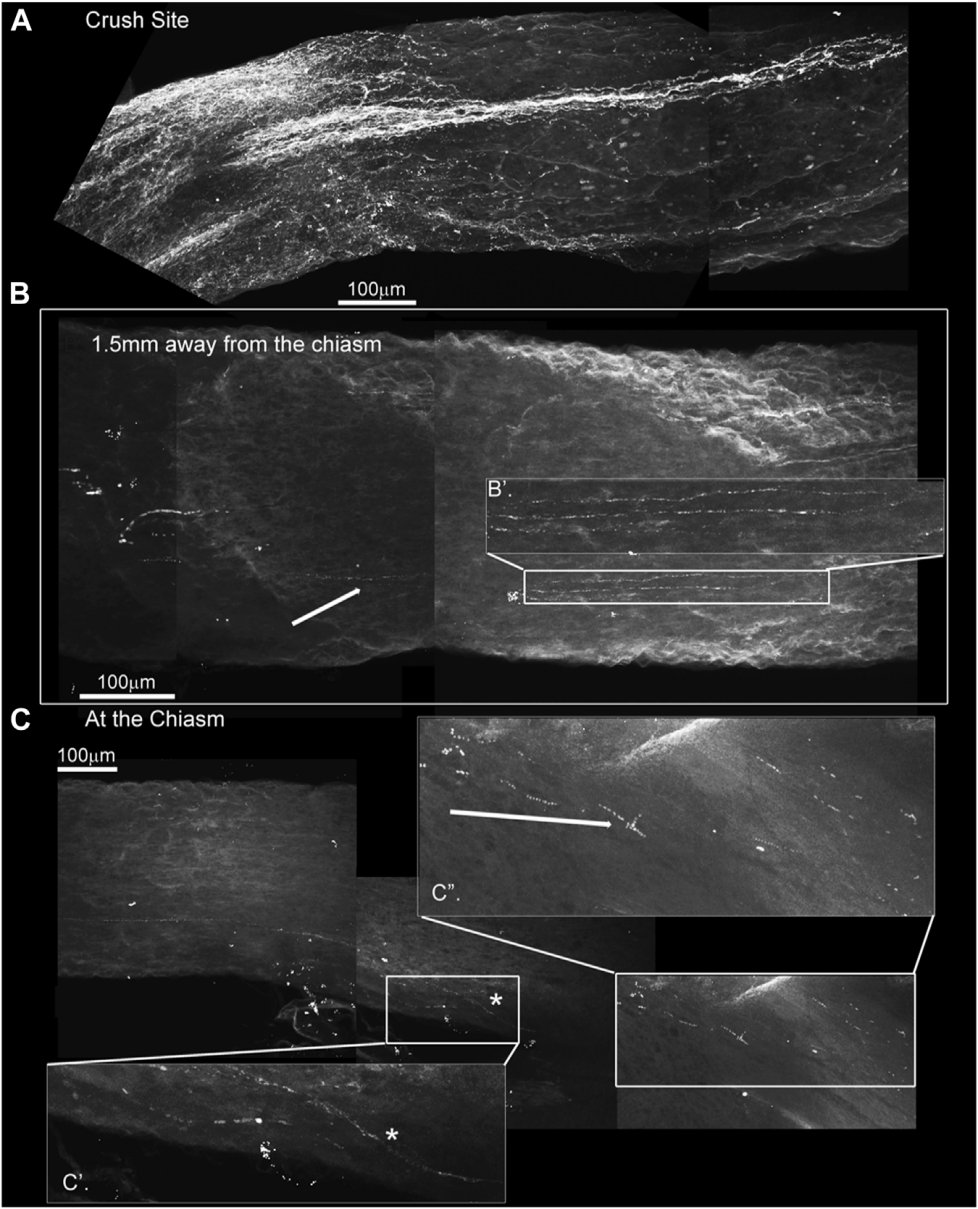
GAP-43 immunostaining using the iDISCO method to stain and chemically clear the entire optic nerve that was crushed and 4-drug treated reveals GAP-43 staining in the chiasm. ONC with 4-drug treatment and i-DISCO GAP-43 staining reveals the crush site with regenerating fibers **(A)**. Approximately 6 mms away from the crush, near the chiasm we also detect GAP-43 positive axons **(B)** the blowup displays several of these GAP-43+ axons. We even detect fibers in the chiasm **(C)** as revealed by the blowups in C’ and C”.

We also assayed endogenous markers of neuronal regeneration. Figure 8A shows GAP-43-expressing axons regenerating past the crush site in a nerve cleared with the iDISCO technique. GAP-43 is a marker of regeneration as it is expressed in growing axons and enriched in the growth cone membrane during periods of axonal extention (Fischer et al., 2017). In this animal, GAP-43 was also detected near and within the optic chiasm (Figures 8B,C, respectively). Magnifications B’, C’, and C’’ highlight fibers 1.5 and 6.0 mm away from the site of injury, once again suggesting extensive axonal regeneration from the crush to the chiasm. Although sparse, GAP-43 immunostaining indicates that the imaged axons are indeed growing and are not senescent spared fibers.

Thus, using three separate labeling approaches, we were able to document that regenerated axons reach the chiasm. Four additional animals with the four-drug treatment combination showed axonal regeneration extending to about 3 mm from the crush site (Table 1). The proportion of animals that displayed significant axonal regeneration is roughly equal to the proportion of animals that displayed restored VEPs and OKRs. Therefore, we reasonably conclude that the functional recovery previously observed can be attributed to robust axonal regeneration.

### Effects of individual drugs and three drug combinations

We tested whether, when delivered either individually or in pairs, the drugs affected axonal regeneration in our ONC model. Immediately following surgery and 3 days after surgery, we treated the injured eye with 5 mg/mL HU-210 or 300 μM IL-6 injected intravitreally, and after 2 weeks we evaluated for regeneration by labeling axons with the axonal marker GAP-43 and sectioning the nerve. Alternatively, axons were labeled with CTB before the animal was sacrificed.

**FIGURE 9:**
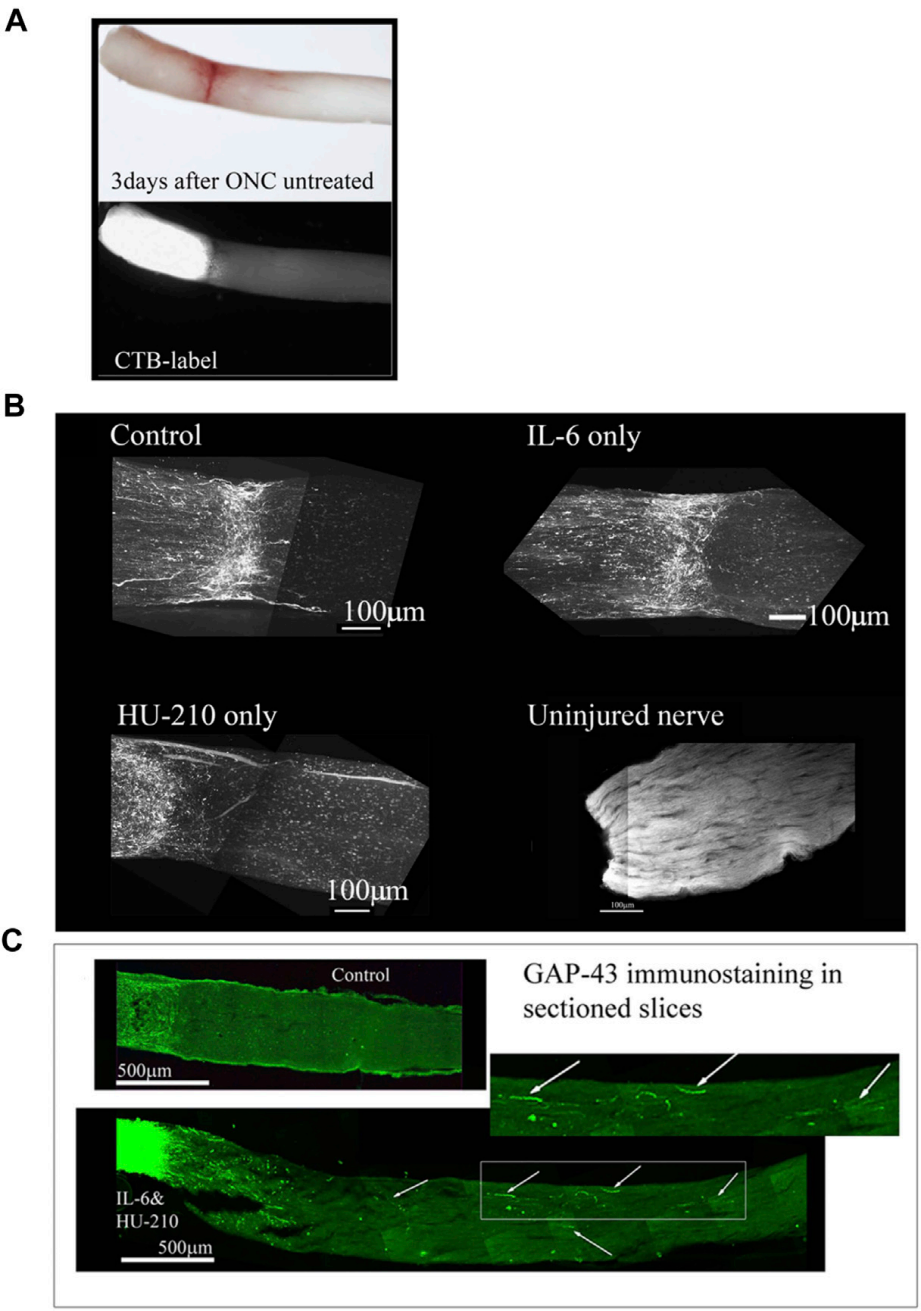
Individual drugs promote limited axonal regeneration. We visualized the whole nerve with CTB labelling using a Multiphoton microscope. **(A)** Control animals injected with 2.5 μL 0.5% DMSO, have few labeled axons crossing a short distance (less than 0.2 mm from the crush site), this was consistent for IL-6 (5 mg/mL) and HU-210 (300 μM) evaluated individually as well, where no significant axonal regeneration was detected past the crush site. Uninjured nerve with CTB labeling and 3-DISCO clearing shows the abundance of axons in the rat optic nerve. **(B)** Untreated crushed optic nerve was labeled with CTB immediately after injury and 3 days after crush we viewed the nerve under the light microscope (top image), and the CTB-label under fluorescence, showing no spared fibers (bottom image). **(C)** Fischer adult rats had unilateral ONC performed followed by intravitreal injections of either 0.5% DMSO for vehicle control (top) or the combination of IL-6 (5 mg/mL) and HU-210 (300 μM, bottom). Intravitreal injections were repeated on day 3 post-crush. After 3 weeks the nerves were removed, sectioned and immunolabeled with GAP-43. Controls had clear crush sites, but no GAP-43 labeled axons crossing the crush site, while IL-6 and HU-210 had regenerating axons nearly 3 mm away as indicated by arrows.

**FIGURE 10:**
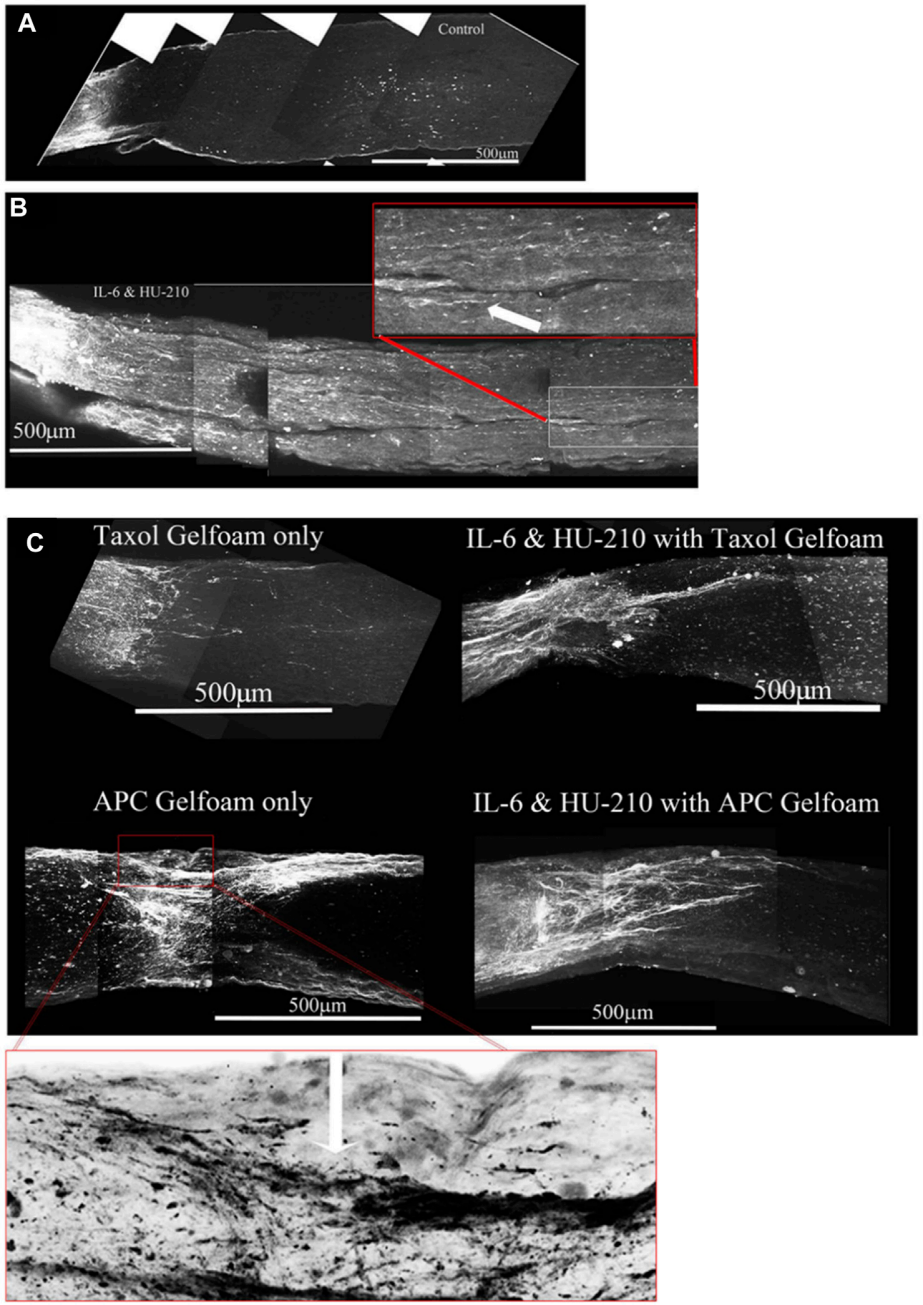
Addition of Taxol and/or APC promotes longer axonal regeneration. **(A)** CTB-labeled regenerating axons within the whole nerve were detected after applying the 3-DISCO clearing technique. Control animals had clear crush sites with few fibers crossing 3 weeks after injury. **(B)** However, with IL-6 and HU-210 treatment we could detect CTB-labeled axons within the nerve bundle shown, above the boxed panel is a magnified region demarcated by the red box, showing the extent of regenerating axons over 2 mm away from the crush site, as pointed out by the white arrows. **(C)** Taxol applied in gelfoam alone had promoted limited axonal regeneration. Adding Taxol gelfoam with IL-6 and HU-210 injections resulted in more robust growth, but still less than 1 mm from the crush site. APC gelfoam alone also promoted modest axonal regeneration from the crush site. Slightly more robust growth is detected when we combine IL-6 and HU-210 with APC gelfoam. The CTB-labeled axons are growing towards the edge where the APC-gelfoam was applied. In the enhanced and inverted image of APC gelfoam demarcated by the red box, we show the crush site that was treated with APC-gelfoam over the injury site. The white arrow in the inverted image of the original emphasizes the crushed fibers that are regenerating, as determined by CTB-labeling.

To evaluate the efficiency of the crush method, we performed ONC with no drug treatment and CTB-labeled the nerves immediately afterwards. When we examined the nerve 3 days later, we observed complete crushes in the brightfield setting and the CTB-label was not detected past the crush site (Figure 9A; Figure 10A). We observed a modest effect of combined HU-210 and IL-6 treatment. We detected and visualized regenerating processes by labeling the axons with CTB coupled to Alexa-488 as previously described, (Mukhopadhyay et al., 1994), chemically clearing the nerves using the 3-DISCO technique and obtaining high intensity projection images (Figure 9B). (Mehta et al., 2007) We also visualized axons by sectioning the nerve and immunostaining with the axonal marker GAP-43 (Figure 9C). Most of the crushed axons without drug treatment had no detectable GAP-43 labeling even 0.2 mm distal to the crush site, while intravitreal injections of a combination of HU-210 + IL-6 resulted in detectable axons nearly 3 mm away from the crush site (Figure 9C, bottom image). Using these techniques, we confirmed that IL-6 and HU-210 in combination promoted axonal regeneration, suggesting that the combination of the two drugs is likely to be synergistic, but not sufficient for extensive regeneration to the chiasm.

The morphological observations here indicates that the effects of HU-210 and IL-6, injected intravitreally, targeting the cell bodies reflect upregulated transcriptional and biosynthetic processes and that these agents have the potential to function as therapeutic agents for axonal regeneration *in vivo* (Cao et al., 2006; Yadaw et al., 2019). In nerves from animals that received intravitreal injections of IL-6 and HU-210, we could observe CTB-labeled fibers over 2 mm from the crush site (Figure 10B). However, in neither Spague-Dawley or Long Evans rats did HU-210- and IL-6-stimulated axonal regeneration reach the optic chiasm. It has been recognized that biosynthesis alone does not result in significant axonal regeneration after injury. Thus, it is unlikely that the effects of HU-210 and IL-6 alone are solely responsible for the functional recovery previously observed. Axonal damage is first induced by neurofilament (microtubule) disassembly, and inflammation can lead to apoptosis and inhibit regeneration (Povlishock, 1993; McDonald and Sadowsky, 2002; Sengottuvel et al., 2011).

Taxol alone in gelfoam had a modest effect on promoting some regenerating fibers as reported earlier (Figure 10C, top left). (Siddiq et al., 2021) APC alone promoted significant axonal regeneration about 0.5 mm past the crush site (Figure 10C, bottom left), as reported earlier (Davies et al., 1999). The effect of APC was most prominent on one edge of the nerve, presumably where we had placed the drug-impregnated gelfoam. In order to ascertain whether these were not spared fibers, we carefully examined the crush site of this nerve treated with APC- highlighted in the red box and an enlarged image where we inverted the image to help reveal the crushed regenerating fibers (red box and in the magnified inverted image)- and observed that the crush was in fact complete, with regenerating CTB-labeled fibers growing along the edge of the nerve. Combining IL-6 and HU-210 with Taxol gelfoam (Figure 10C, top right) produced a more robust response compared to the three agents individually or IL-6 and HU-210 combined, growing over 0.5 mm distal to the crush site. More extensive growth was observed when APC was combined with IL-6 and HU-210 (Figure 10C, bottom right). However, none of the drugs tested could promote extensive axonal regeneration individually or in three drug combinations, as shown in the statistical analysis of axon length presented in Figure 5.

## Discussion

In this study, we designed a drug therapy for a complex pathophysiology using systems-based logic. We considered the multiple subcellular processes occurring at distinct locations within the cell that could be involved in axonal preservation and regeneration and identified four drugs that could regulate these subcellular processes. Three of these four drugs had previously never been tested for axonal regeneration activity *in vivo*. Hence, our logic was based on *in vitro* experiments, mostly on cultured neurons. We focused on drugs that would increase capacity for regeneration at the neuronal cell body, or promote preservation of injured axons and enhance the ability of the axons to grow longer by modulating local subcellular processes in the axonal shaft. However, our drugs could regulate recovery of spared fibers as well as regeneration of severed ones. For example, microtubules may break following ONC without the total loss of the axon, but the four drug combination stabilizes existing microtubules enough to bridge the lesion. Our experiments do not allow us to distinguish between these different effects. We assumed that the process of neurite outgrowth *in vitro* involves many if not all the subcellular processes that neurons use to regenerate axons *in vivo*. Cell signaling experiments in our laboratory had shown that transcriptional regulation through STAT3 and CREB played a key role in neurite outgrowth (Farber and Diamond, 1948; Yadaw et al., 2019). Independent studies had shown a role for the cAMP pathway and SOCS3, (Filbin, 2004), a STAT inhibitor (Quarta et al., 2014). Hence, we reasoned that application of receptor ligands that stimulate the STAT3 pathway among other transcriptional regulators could increase the intrinsic capacity of neurons to regenerate (Cao et al., 2006). Since CB-1 and IL-6 receptors are expressed in adult neurons, we selected agonists for these receptors as pro-regenerative drugs. *In vitro* experiments showed that application of these drugs at the cell body was more effective in promoting neurite outgrowth, and thus *in vivo* we applied the drug at the cell body as well. As we used the optic nerve crush model, we injected the receptor agonists intravitreally so that they could act on the RGC cell bodies. This resulted in modest but significant drug-stimulated axon regeneration beyond the site of injury. Furthermore, the regeneration occurred rapidly, or the preservation occurred, within a time frame that allows RGC axons to reconnect to post-synaptic targets, preventing the loss of lateral geniculate, superior colliculus, and subcortical neurons. These experiments provided us with an important guideline: that not only should we select the right subcellular process to target, but also that the drug should be applied at the right location. Using this reasoning we selected the two core sets of subcellular processes that function within the growing axon and could targeted by drugs: microtubule growth, and membrane vesicle fusion at the growth cone. Since Taxol stabilizes dynamic microtubules, thus allowing axonal processes to grow, we applied Taxol to the growing axonal regions. By integrating observations in the literature, (Shim et al., 2005; Hellal et al., 2011), we hypothesized that the debris at the injury site including myelin-associated proteins might inhibit axonal growth by inhibiting the exocytotic delivery of new membranes to the growth cone, which is needed for axonal growth. Hence, we selected a locally acting protease that could clear the debris field and block these inhibitory agents. Our experiments show that this combination of increasing capacity at the cell body and modulating subcellular processes in the growing axon enables extensive regeneration from the site of injury near the eye to the chiasm. This was a novel effect that we could not have predicted from the *in vitro* studies alone. *In vitro* studies indicated that treatment with IL-6 protected primary neurons from cell death. As a neuroprotective effect could generate the functional and physiological effects we observed, we carefully examined the appearance of axons in our biochemical assays. Axons extending past the injury site demonstrated tortured growth, and axons in the optic chiasm demonstrated a lack of guidance cues. Based on these observations, we concluded that at least some axons were new growths (Davies et al., 1999; Steward et al., 2008).

**FIGURE 11:**
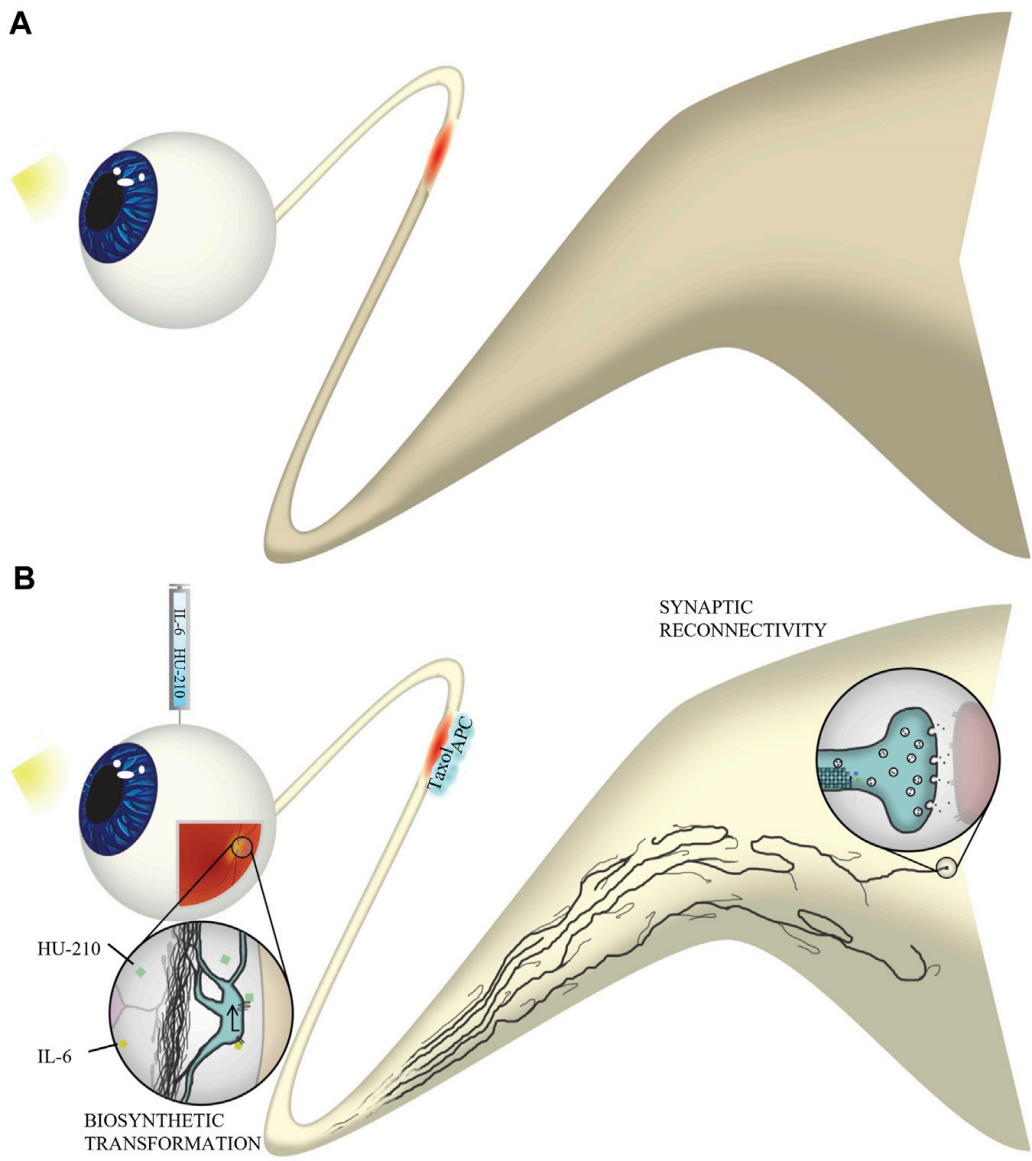
Experimental overview. **(A)** Without four-drug intervention, axons do not regenerate through the injured optic nerve. Natural stimulation with light does not result in visual activity in the brain. **(B)** Following optic nerve crush, we injected IL-6 and HU-210 into the vitreous and applied Taxol and APC in gelfoam at the injury site. IL-6 and HU-210 promoted oncogenic transformation at the cell body of retinal ganglion cells through activation of the STAT3 transcription pathway. Taxol stabilizes dynamic microtubules at the injury site, which works synergistically with APC, which clears the inhibitory environment and allows for fusion of membrane vesicles with the growth cone plasma membrane, to promote axon elongation. Axons regenerate to the optic chiasm and even make synaptic connections with the superior colliculus. Thus, natural stimulation with light results in function visual activity in our optic nerve crush model of axonal regeneration.

Any level of functional recovery after complete injury is encouraging, and one question we are left with is why some animals experienced recovery of simple visual behavior while others seemed to have not therapeutic effect. We hypothesize that the size of the animal may contribute to the magnitude of recovery. Other likely factors may be the length of time the animal was under anesthesia during the optic nerve crush procedure, or whether there was a significant amount of bleeding during the surgery. Damage to structures near the optic nerve, such as the frontal sinus, could result in increased inflammation. Additionally, some animals may not be responsive to drug therapy for reasons we do not understand. It is very rare that a drug works on all individuals. As axonal injury, via ischemia and reperfusion injury, is implicated in neuronal apoptosis, we anticipated RGC death in our optic nerve crush model. While some modest changes in the fERG were observed, we found that optic nerve crush did not significantly alter the activity of retinal neurons. Importantly, the observed abnormalities were absent in animals that received drug intervention. We also find that we were able to partially restore electrophysiological communication from the eye to the brain. Since some of our drugs, particularly APC and Taxol are delivered to the injury site they could be protecting and preserving injured axons. Thus restoration of function could be attributed to both preservation and regeneration. A more extensive study in the future is required to better understand the extent of regeneration compared to preservation of injured axons. Here we conclude that both preservation of injured axons and regeneration of severed axons give rise to functional recovery of vision-dependent behavior, as animals with partially-restored VEPs also exhibited an OKR. Our results provide an example of the systems therapeutic approach enabling a rational basis for the design of combination therapies applied in a spatially specified manner to treat complex pathophysiologies, in this case nerve damage in the CNS. A summary illustration of our findings can be found in Figure 11.

### Study limitations and future experiments

Although it is encouraging that we have an identifiable path for a systems logic-based design of therapeutics, much further work is needed to translate these findings into viable treatments even in animal models. The study has multiple limitations: We have not considered dosing regimens or adverse events associated with this drug therapy. Also, we do not understand the reasons for animal -to animal variations that we see. Some of these variations may be somatic genomics or epigenetics. Further experiments are needed to understand the variability in drug responsiveness In spite of these limitations this exploratory study shows a reproducible but limited recovery in a small set of injured animals. Future experiments will evaluate both different dosing regimens and multiple therapeutic time points to determine if the multidrug treatment can have greater efficacy in a larger number of animals. Such studies could make these results more clinically relevant. We also need additional strategies to further establish the relationship between the recovery of electrophysiological function and vision-dependent behaviors in the whole animal. Studies are also required to better address the extent of preservation of injured axons compared to the number of regenerating axons. These types of experiments will form the basis for future studies. Nevertheless, since observations in the optic nerve crush model are most often applied to spinal cord injury, it will be useful to assess if this four-drug combination can partially reverse spinal cord injury and restore some movement in future experiments.

## Data Availability

The raw data supporting the conclusion of this article will be made available by the authors, without undue reservation.

## Abbreviations

CB1R, cannabinoid 1 receptor; APC, Activated Protein C; CNS, Central Nervous System; ONC, Optic Nerve Crush; NOG, Neurite Outgrowth Assay; CTB, Cholera Toxin B; MLN, Myelin; VEP, visual evoked potential; CAP, Compound Action Potential; OKR, optokinetic response.

## References

[B1] Alushin G. M. Lander G. C. Kellogg E. H. Zhang R. Baker D. Nogales E. (2014). High-resolution microtubule structures reveal the structural transitions in αβ-tubulin upon GTP hydrolysis. Cell 157, 1117–1129. 10.1016/j.cell.2014.03.053 24855948 PMC4054694

[B2] Ashique S. Sirohi E. Kumar S. Rihan M. Mishra N. Bhatt S. (2023). Aducanumab in alzheimer's disease: A critical update. Curr. Med. Chem. 31. 10.2174/0929867331666230727103553 37497712

[B3] Bei F. Lee H. H. C. Liu X. Gunner G. Jin H. Ma L. (2016). Restoration of visual function by enhancing conduction in regenerated axons. Cell 164, 219–232. 10.1016/j.cell.2015.11.036 26771493 PMC4863988

[B4] Bromberg K. D. Maayan A. Neves S. R. Iyengar R. (2008). Design Logic of a cannabinoid receptor signaling network that triggers neurite outgrowth. Science 320, 903–909. 10.1126/science.1152662 18487186 PMC2776723

[B5] Buchli A. D. Schwab M. E. (2005). Inhibition of Nogo: a key strategy to increase regeneration, plasticity and functional recovery of the lesioned central nervous system. Ann. Med. 37, 556–567. 10.1080/07853890500407520 16338758

[B6] Cajal R. Y. (1991). Cajal’s degeneration and regeneration of the nervous system. Oxford Press.

[B7] Cao Z. Gao Y. Bryson J. B. Hou J. Chaudhry N. Siddiq M. (2006). The cytokine interleukin-6 is sufficient but not necessary to mimic the peripheral conditioning lesion effect on axonal growth. J. Neurosci. 26, 5565–5573. 10.1523/JNEUROSCI.0815-06.2006 16707807 PMC6675293

[B8] Chandran V. Coppola G. Nawabi H. Omura T. Versano R. Huebner E. A. (2016). A systems-level analysis of the peripheral nerve intrinsic axonal growth program. Neuron 89, 956–970. 10.1016/j.neuron.2016.01.034 26898779 PMC4790095

[B9] Chen Y. A. Scales S. J. Scheller R. H. (2001). Sequential SNARE assembly underlies priming and triggering of exocytosis. Neuron 30 (1), 161–170. 10.1016/s0896-6273(01)00270-7 11343652

[B10] Creel D. J. (2019). Visually evoked potentials. Handb. Clin. neurology 160, 501–522. 10.1016/B978-0-444-64032-1.00034-5 31277872

[B11] Davies S. J. Goucher D. R. Doller C. Silver J. (1999). Robust regeneration of adult sensory axons in degenerating white matter of the adult rat spinal cord. J. Neurosci. 19 (14), 5810–5822. 10.1523/JNEUROSCI.19-14-05810.1999 10407022 PMC6783087

[B12] de Lima S. Koriyama Y. Kurimoto T. Oliveira J. T. Yin Y. Li Y. (2012). Full-length axon regeneration in the adult mouse optic nerve and partial recovery of simple visual behaviors. Proc. Natl. Acad. Sci. U. S. A. 09 (23), 9149–9154. 10.1073/pnas.1119449109 PMC3384191 22615390

[B13] Dent E. W. Callaway J. L. Zxebenyi G. Baas P. W. Kalil K. (1999). Reorganization and movement of microtubules in axonal growth cones and developing interstitial branches. J. Neurosci. 19 (20), 8894–8908. 10.1523/JNEUROSCI.19-20-08894.1999 10516309 PMC6782770

[B14] Douglas R. M. Alam N. M. Silver B. D. McGill T. J. Tschetter W. W. Prusky G. T. (2005). Independent visual threshold measurements in the two eyes of freely moving rats and mice using a virtual-reality optokinetic system. Vis. Neurosci. 22 (5), 677–684. 10.1017/S0952523805225166 16332278

[B15] Ertürk A. Mauch C. P. Hellal F. Förstner F. Keck T. Becker K. (2012). Three-dimensional imaging of the unsectioned adult spinal cord to assess axon regeneration and glial responses after injury. Nat. Med. 18, 166–171. 10.1038/nm.2600 22198277

[B16] Farber S. Diamond L. K. (1948). Temporary remissions in acute leukemia in children produced by folic acid antagonist, 4-aminopteroyl-glutamic acid. N. Engl. J. Med. 238 (23), 787–793. 10.1056/NEJM194806032382301 18860765

[B17] Fewou S. N. Plomp J. J. Willison H. J. (2014). The pre-synaptic motor nerve terminal as a site for antibody-mediated neurotoxicity in autoimmune neuropathies and synaptopathies. J. Anat. 224, 36–44. 10.1111/joa.12088 23937354 PMC3867885

[B18] Filbin M. T. (2004). Myelin-associated inhibitors of axonal regeneration in the adult mammalian CNS. Nat. Rev. Neurosci. 4, 703–713. 10.1038/nrn1195 12951563

[B19] Fischer D. Harvey A. R. Pernet V. Lemmon V. P. Park K. K. (2017). Optic nerve regeneration in mammals: Regenerated or spared axons? Exp. Neurol. 296, 83–88. 10.1016/j.expneurol.2017.07.008 28716559 PMC5564230

[B20] Galtrey C. M. Fawcett J. W. (2007). The role of chondroitin sulfate proteoglycans in regeneration and plasticity in the central nervous system. Brain Res. Rev. 54, 1–18. 10.1016/j.brainresrev.2006.09.006 17222456

[B21] Goodman L. S. Wintrobe M. M. Dameshek W. Goodman M. J. Gilman A. McLennan M. T. (1984). Landmark article Sept. 21, 1946: Nitrogen mustard therapy. Use of methyl-bis(beta-chloroethyl)amine hydrochloride and tris(beta-chloroethyl)amine hydrochloride for Hodgkin's disease, lymphosarcoma, leukemia and certain allied and miscellaneous disorders. By Louis S. Goodman, Maxwell M. Wintrobe, William Dameshek, Morton J. Goodman, Alfred Gilman and Margaret T. McLennan. JAMA 251 (17), 2255–2261. 10.1001/jama.251.17.2255 6368885

[B22] He J. C. Gomes I. Nguyen T. Jayaram G. Ram P. T. Devi L. A. (2005). The G alpha(o/i)-coupled cannabinoid receptor-mediated neurite outgrowth involves Rap regulation of Src and Stat3. J. Biol. Chem. 280, 33426–33434. 10.1074/jbc.M502812200 16046413

[B23] He Z. Jin Y. (2016). Intrinsic control of axon regeneration. Neuron 90, 437–451. 10.1016/j.neuron.2016.04.022 27151637

[B24] Hellal F. Hurtado A. Ruschel J. Flynn K. C. Laskowski C. J. Umlauf M. (2011). Microtubule stabilization reduces scarring and causes axon regeneration after spinal cord injury. Science 331, 928–931. 10.1126/science.1201148 21273450 PMC3330754

[B25] Jordan J. D. He J. C. Eungdamrong N. J. Gomes I. Ali W. Nguyen T. (2005). Cannabinoid receptor-induced neurite outgrowth is mediated by Rap1 activation through G(alpha)o/i-triggered proteasomal degradation of Rap1GAPII. J. Biol. Chem. 280, 11413–11421. 10.1074/jbc.M411521200 15657046

[B52] Khan A. Mar K. F. Brown W. A. (2021). Consistently modest antidepressant effects in clinical trials: the role of regulatory requirements. Psypharmacol. Bull. 51 (3), 79–108. 10.64719/pb.4412 PMC8374926 34421147

[B26] Leon S. Yin Y. Nguyen J. Irwin N. Benowitz L. I. (2000). Lens injury stimulates axon regeneration in the mature rat optic nerve. J. Neurosci. 20, 4615–4626. 10.1523/JNEUROSCI.20-12-04615.2000 10844031 PMC6772462

[B27] Lim J. H. Stafford B. K. Nguyen P. L. Lien B. V. Wang C. Zukor K. (2016). Neural activity promotes long-distance, target-specific regeneration of adult retinal axons. Nat. Neurosci. 19 (8), 1073–1084. 10.1038/nn.4340 27399843 PMC5708130

[B28] Luo X. Salgueiro Y. Beckerman S. R. Lemmon V. P. Tsoulfas P. Park K. K. (2013). Three-dimensional evaluation of retinal ganglion cell axon regeneration and pathfinding in whole mouse tissue after injury. Exp. Neurol. 247, 653–662. 10.1016/j.expneurol.2013.03.001 23510761 PMC3726550

[B29] McDonald J. W. Sadowsky C. (2002). Spinal-cord injury. Lance 359 (9304), 417–425. 10.1016/S0140-6736(02)07603-1 11844532

[B30] Mehta N. R. Lopez P. H. Vyas A. A. Schnaar R. L. (2007). Gangliosides and Nogo receptors independently mediate myelin-associated glycoprotein inhibition of neurite outgrowth in different nerve cells. J. Biol. Chem. 282, 27875–27886. 10.1074/jbc.M704055200 17640868 PMC2377359

[B31] Mimura F. Yamagishi S. Arimura N. Fujitani M. Kubo T. Kaibuchi K. (2006). Myelin-associated glycoprotein inhibits microtubule assembly by a Rho-kinase-dependent mechanism. J. Biol. Chem. 281 (23), 15970–15979. 10.1074/jbc.M510934200 16595691

[B32] Mosnier L. O. Zlokovic B. V. Griffin J. H. (2007). The cytoprotective protein C pathway. Blood 109, 3161–3172. 10.1182/blood-2006-09-003004 17110453

[B33] Mukhopadhyay G. Doherty P. Walsh F. S. Crocker P. R. Filbin M. T. (1994). A novel role for myelin-associated glycoprotein as an inhibitor of axonal regeneration. Neuron 13, 757–767. 10.1016/0896-6273(94)90042-6 7522484

[B34] Park K. K. Liu K. Hu Y. Smith P. D. Wang C. Cai B. (2008). Promoting axon regeneration in the adult CNS by modulation of the PTEN/mTOR pathway. Science 322, 963–966. 10.1126/science.1161566 18988856 PMC2652400

[B35] Povlishock J. T. (1993). Pathobiology of traumatically induced axonal injury in animals and man. Ann. Emerg. Med. 22 (6), 980–986. 10.1016/s0196-0644(05)82738-6 8503536

[B36] Quarta S. Baeumer B. E. Scherbakov N. Andratsch M. Rose-John S. Dechant G. (2014). Peripheral nerve regeneration and NGF-dependent neurite outgrowth of adult sensory neurons converge on STAT3 phosphorylation downstream of neuropoietic cytokine receptor gp130. J. Neurosci. 34, 13222–13233. 10.1523/JNEUROSCI.1209-13.2014 25253866 PMC4172810

[B37] Ram P. T. Horvath C. M. Iyengar R. (2000). Stat3-mediated transformation of NIH-3T3 cells by the constitutively active Q205L Galphao protein. Science 287, 142–144. 10.1126/science.287.5450.142 10615050

[B38] Renier N. Wu Z. Simon D. J. Yang J. Ariel P. Tessier-Lavigne M. (2014). iDISCO: A simple, rapid method to immunolabel large tissue samples for volume imaging. Cell 159, 896–910. 10.1016/j.cell.2014.10.010 25417164

[B39] Sengottuvel V. Leibinger M. Pfreimer M. Fischer D. (2011). Taxol facilitates axon regeneration in the mature CNS. J. Neurosci. 16, 2688–2699. 10.1523/JNEUROSCI.4885-10.2011 PMC6623712 21325537

[B40] Shim S. Goh E. L. Ge S. Sailor K. Yuan J. P. Roderick H. L. (2005). XTRPC1-dependent chemotropic guidance of neuronal growth cones. Nat. Neuro 8, 730–735. 10.1038/nn1459 PMC4005724 15880110

[B41] Siddiq M. M. Hannila S. S. Carmel J. B. Bryson J. B. Hou J. Nikulina E. (2015). Metallothionein-I/II promotes axonal regeneration in the central nervous system. J. Biol. Chem. 290, 16343–16356. 10.1074/jbc.M114.630574 25947372 PMC4481232

[B42] Siddiq M. M. Hannila S. S. Zorina Y. Nikulina E. Rabinovich V. Hou J. (2021). Extracellular histones, a new class of inhibitory molecules of CNS axonal regeneration. Brain Commun. 3 (4), fcab271. 10.1093/braincomms/fcab271 34993473 PMC8728726

[B43] Simpson J. I. (1984). The accessory optic system. Annu. Rev. Neurosci. 7, 13–41. 10.1146/annurev.ne.07.030184.000305 6370078

[B44] Steward O. Zheng B. Tessier-Lavigne M. Hofstadter M. Sharp K. Yee K. M. (2008). Regenerative growth of corticospinal tract axons via the ventral column after spinal cord injury in mice. J. Neurosci. 28 (27), 6836–6847. 10.1523/JNEUROSCI.5372-07.2008 18596159 PMC2745399

[B45] Strittmatter S. M. Igarashi M. Fishman M. C. (1994). GAP-43 amino terminal peptides modulate growth cone morphology and neurite outgrowth. J. Neurosci. 14 (9), 5503–5513. 10.1523/JNEUROSCI.14-09-05503.1994 8083750 PMC6577098

[B46] Sun F. Park K. K. Belin S. Wang D. Lu T. Chen G. (2011). Sustained axon regeneration induced by co-deletion of PTEN and SOCS3. Nature 480, 372–375. 10.1038/nature10594 22056987 PMC3240702

[B47] Tang X. Tzekov R. Passaglia C. L. (2016). Retinal cross talk in the mammalian visual system. J. Neurophysiol. 115, 3018–3029. 10.1152/jn.01137.2015 26984426 PMC4946590

[B48] Wang Y. Zhao Z. Rege S. V. Wang M. Si G. Zhou Y. (2016). 3K3A-activated protein C stimulates postischemic neuronal repair by human neural stem cells in mice. Nat. Med. 22, 1050–1055. 10.1038/nm.4154 27548576 PMC5215920

[B49] Wu Y. Y. Bradshaw R. A. (1996). Induction of neurite outgrowth by interleukin-6 is accompanied by activation of Stat3 signaling pathway in a variant PC12 cell (E2) line. J. Biol. Chem. 271, 13023–13032. 10.1074/jbc.271.22.13023 8662645

[B50] Yadaw A. Siddiq M. M. Rabinovich V. Iyengar R. Hansen J. (2019). Dynamic balance between vesicle transport and microtubule growth enables neurite outgrowth. PLoS Comput. Biol. 15 (5), e1006877. 10.1371/journal.pcbi.1006877 31042702 PMC6546251

[B51] Zorina Y. Iyengar R. Bromberg K. D. (2010). Cannabinoid 1 receptor and interleukin-6 receptor together induce integration of protein kinase and transcription factor signaling to trigger neurite outgrowth. J. Biol. Chem. 285, 1358–1370. 10.1074/jbc.M109.049841 19861414 PMC2801262

