# Supplementary material for: A spatially specified systems pharmacology therapy for axonal recovery after injury
Source: Front Pharmacol. 2023 Sep 20;14:1225759. doi: 10.3389/fphar.2023.1225759 (PMC10547904; doi:10.3389/fphar.2023.1225759)
Supplement: Supplementary file 1 [file DataSheet1.PDF]

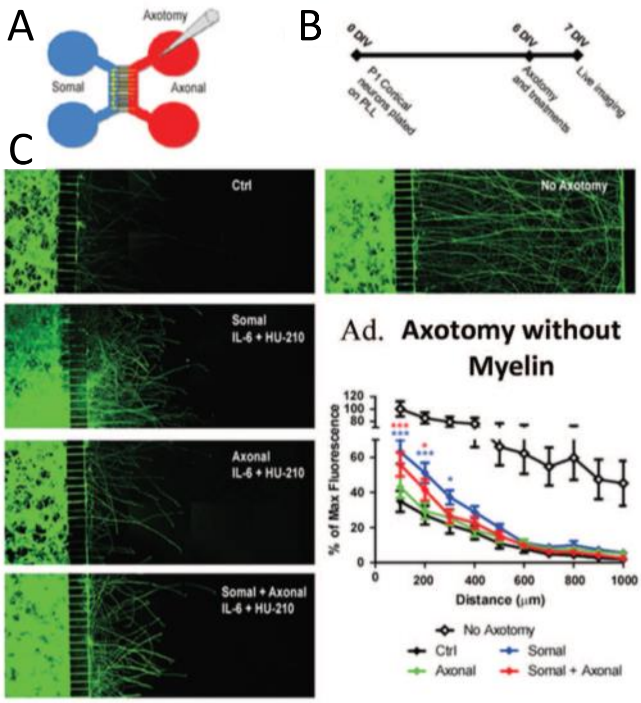
A
Axotomy
Somal
Axonal
B
0 DIV
6 DIV
7 DIV
P1 Cortical neurons plated on PLL
Axotomy and treatments
Live imaging
C
Ctrl
No Axotomy
Somal
IL-6 + HU-210
Axonal
IL-6 + HU-210
Somal + Axonal
IL-6 + HU-210
Ad. Axotomy without Myelin
% of Max Fluorescence
Distance (μm)
No Axotomy
Ctrl
Somal
Axonal
Somal + Axonal

Figure S1. Somal application of IL-6 and HU-210 results in enhanced neurite outgrowth after axotomy. Cortical neurons were plated in the somal compartment (blue) of microfluidic chambers, schematically represented in (A) and allowed to grow axons across the microgrooves into the axonal compartment (red). The gray cone depicts a pipet used to perform axotomy by aspirating medium from the axonal compartment. (B) For axotomy experiments, neurons were cultured in chambers on PLL for 6-7 days, and axotomy was performed by aspirating all medium from the axonal compartments 2-4 times.. 5 ng/ml IL-6 and 100 nM HU- 210 were added together to the somal, axonal or both compartments after axotomy. Treatments were added to the indicated compartments in neurobasal (NB) immediately after axotomy, and neurons were imaged live after 24 hrs using Calcein AM. (C) Representative confocal images after axotomy and treatment. (Ad.) Neurite outgrowth was quantified by measuring the total fluorescence at multiple cross-sections of the image. The zero point on the x-axis represents the right edge of microgrooves. All data points were normalized to the first point of "No Axotomy" at 100 µm from the microgrooves as 100 percent. Statistical differences were calculated from two independent experiments using two-way ANOVA. Asterisks show significance compared to Ctrl treatment at a given distance; *, $p < 0.05$; ***, $p < 0.001$.

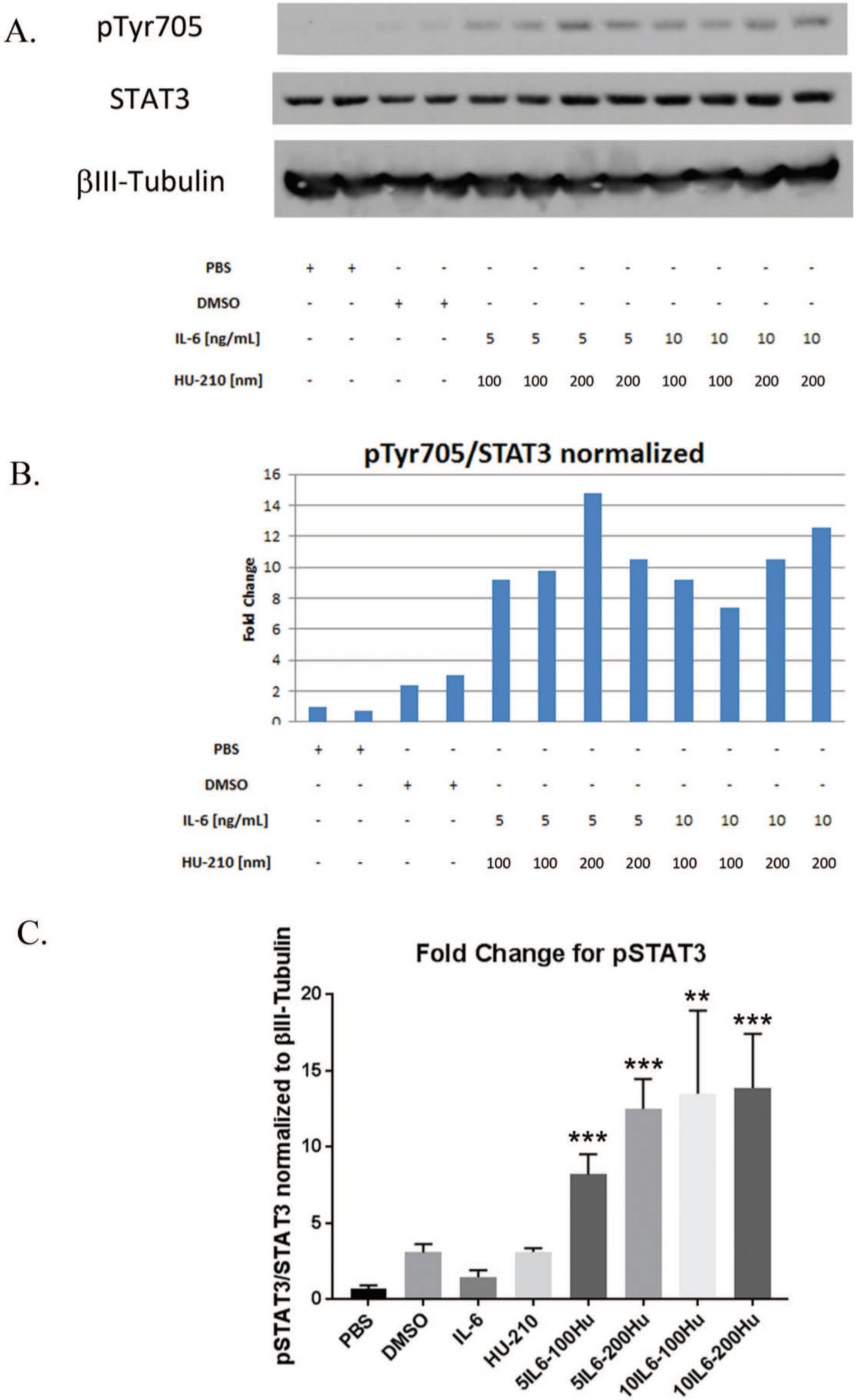
A.
pTyr705
STAT3
βIII-Tubulin
PBS + + - - - - - - - - - -
DMSO - - + + - - - - - - - -
IL-6 [ng/mL] - - - - 5 5 5 5 10 10 10 10
HU-210 [nm] - - - - 100 100 200 200 100 100 200 200
B.
pTyr705/STAT3 normalized
Fold Change
0
2
4
6
8
10
12
14
16
PBS + + - - - - - - - - - -
DMSO - - + + - - - - - - - -
IL-6 [ng/mL] - - - - 5 5 5 5 10 10 10 10
HU-210 [nm] - - - - 100 100 200 200 100 100 200 200
C.
Fold Change for pSTAT3
pSTAT3/STAT3 normalized to βIII-Tubulin
0
5
10
15
20
***
***
**
***
PBS
DMSO
IL-6
HU-210
5IL6-100Hu
5IL6-200Hu
10IL6-100Hu
10IL6-200Hu

Figure S2: Combination of IL-6 and HU-210 activates STAT-3. (A) Cortical neurons in nonsupplemented neurbasal media were treated with IL-6 and HU-210 or controls as described for 2 hrs. The cells were lysed and subjected to immunoblot analysis ofr phospho-STAT3 (pTyr705), total STAT3 and βIII-tubulin. (B) We normalized the samples for phospho-STAT3 to total STAT3, and fold changes are displayed. (C) In three independent experiments, we averaged phospho-STAT3 to total STAT3 normalized to βIII-tubulin. For controls we used PBS and 0.5% DMSO. For the treated samples we used 5 ng/ml IL-6 and 100 nM HU-210 (5IL6-100Hu), 5 ng/ml IL-6 and 200 nM HU-210 (5IL6-200Hu), 10 ng/ml IL-6 and 100 nM HU-210 (10IL6-100Hu) and 10 ng/ml and 200 nM HU-210 (10IL6-200Hu). All statistics are unpaired t-test, **$p<0.01$, ***$p<0.001$.

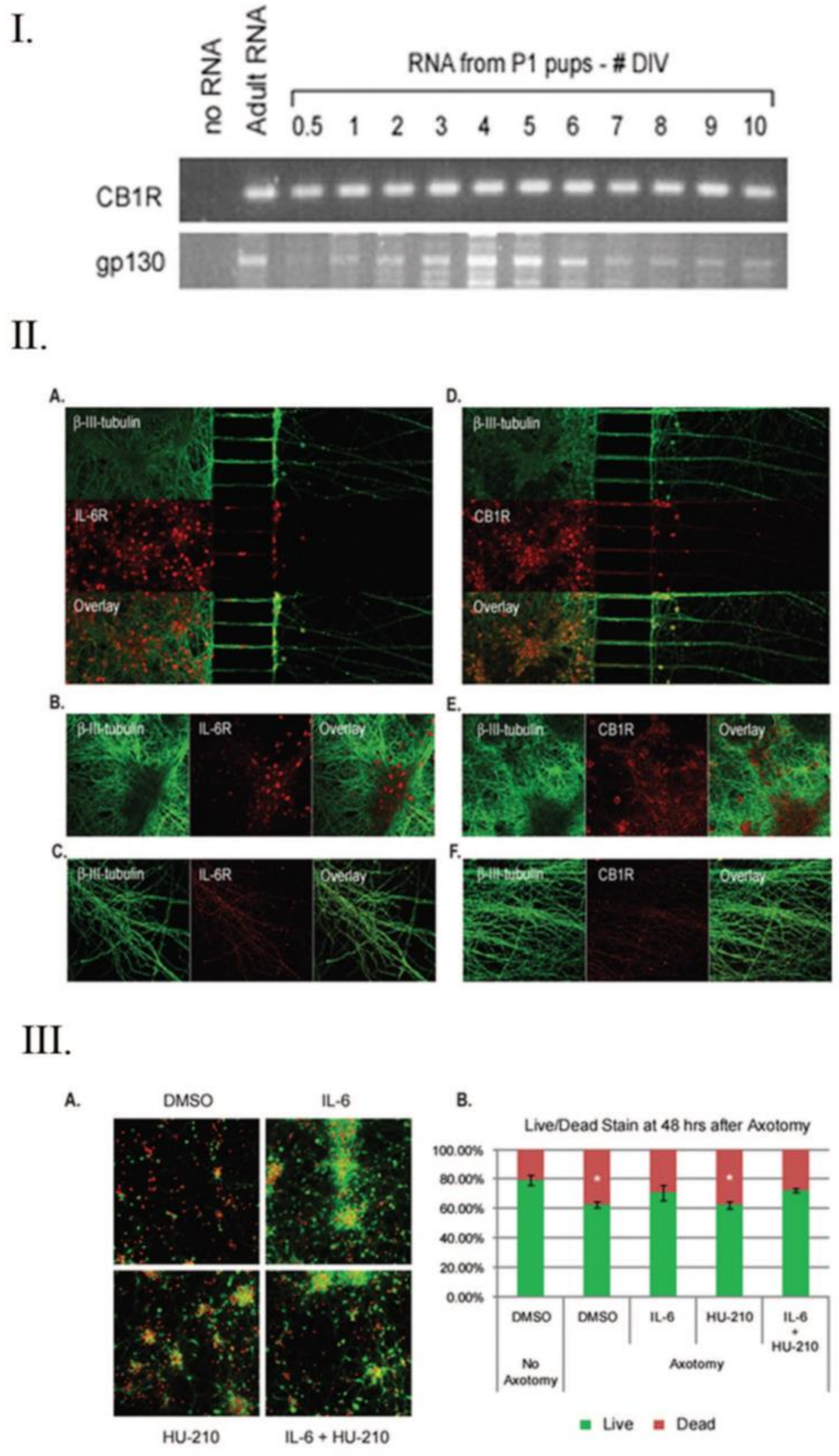
I.
no RNA
Adult RNA
RNA from P1 pups - # DIV
0.5
1
2
3
4
5
6
7
8
9
10
CB1R
gp130
II.
A.
β-III-tubulin
IL-6R
Overlay
D.
β-III-tubulin
CB1R
Overlay
B.
β-III-tubulin
IL-6R
Overlay
E.
β-III-tubulin
CB1R
Overlay
C.
β-III-tubulin
IL-6R
Overlay
F.
β-III-tubulin
CB1R
Overlay
III.
A.
DMSO
IL-6
HU-210
IL-6 + HU-210
B.
Live/Dead Stain at 48 hrs after Axotomy
100.00%
80.00%
60.00%
40.00%
20.00%
0.00%
DMSO
DMSO
IL-6
HU-210
IL-6 + HU-210
No Axotomy
Axotomy
Live
Dead

Figure S3-I: CB1R and IL-6R expression in rat cortical neurons. P1 cortical neurons were plated on poly-L-lysine-coated dishes for the indicated number of days *in vitro*, and mRNA was isolated and subjected to RT-PCR for CB1R or gp130.

Figure S3-II: IL-6R and CB1R are expressed strongly in the soma and weakly in the axons of cortical neurons. Cortical neurons were plated in microfluidic chambers and allowed to grow axons across the microgrooves for 6-7 days. Neurons were fixed and immunolabeled for β- III-tubulin (green) and (A-C) IL-6R (red) or (D-F) CB1R (red), to determine the location of receptors. (A, D) Tiled composite images of neurons in microfluidic chambers. (B, E) Representative fields of neurons in the somal compartments. (C, F) Representative fields of neurons in the axonal compartments.

Figure S3-III: Somal treatment with IL-6 promotes neuronal survival after axotomy. Neurons were grown in chambers for 7 days, starved in NBS for 1-2 h, subjected to axotomy and treated for 48 h. Calcein AM (green) and ethidium homodimer-1 (red) were applied to the somal compartment and live imaging was performed. Images were taken from regions immediately to the left of the microgrooves to ensure imaging neurons that were previously axotomized. (A) Representative confocal images after axotomy and treatment. (B) Quantification of total green to red fluorescence ratio was performed by measuring number of pixels of each color. Statistical differences from two independent experiments were calculated using a one-way ANOVA followed by Bonferroni's multiple comparison test. Asterisks show significance in comparison to "No Axotomy"; *, $p < 0.05$.

UNINJURED VEP

I.

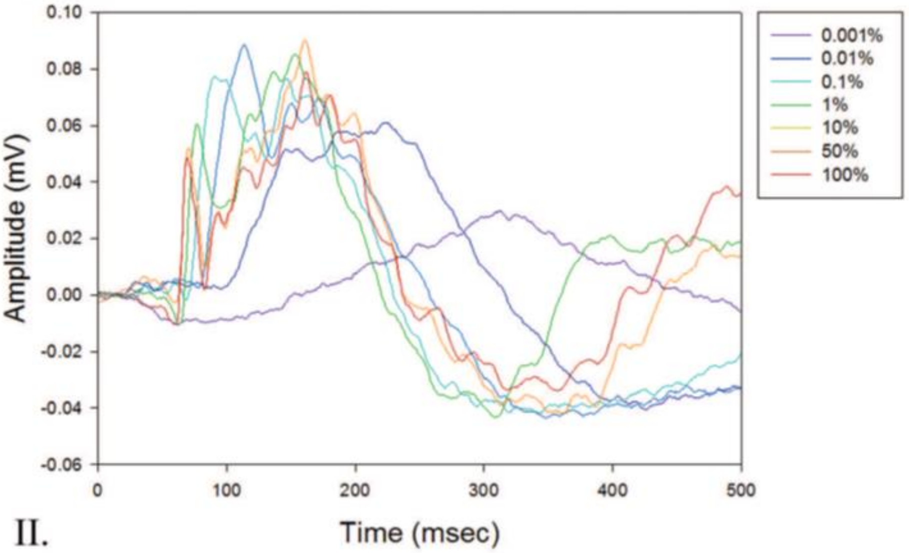

II.

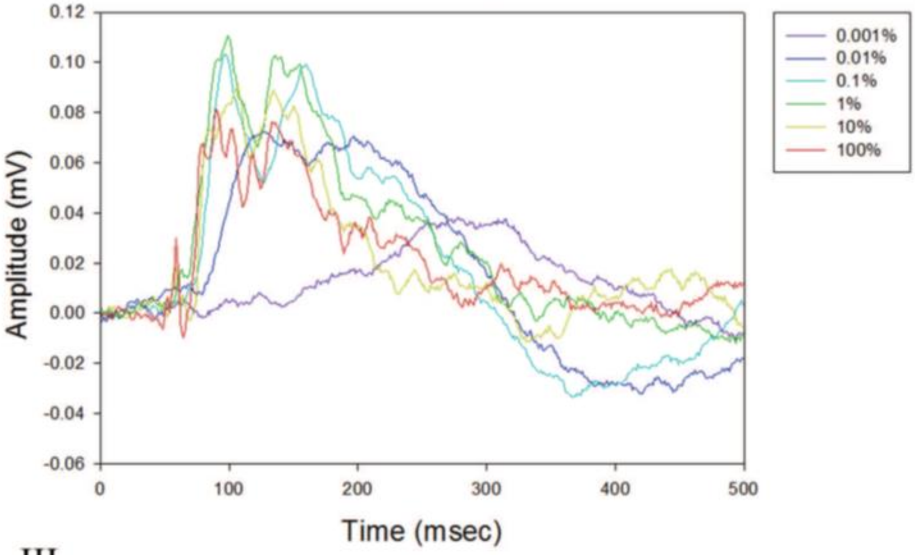

III.

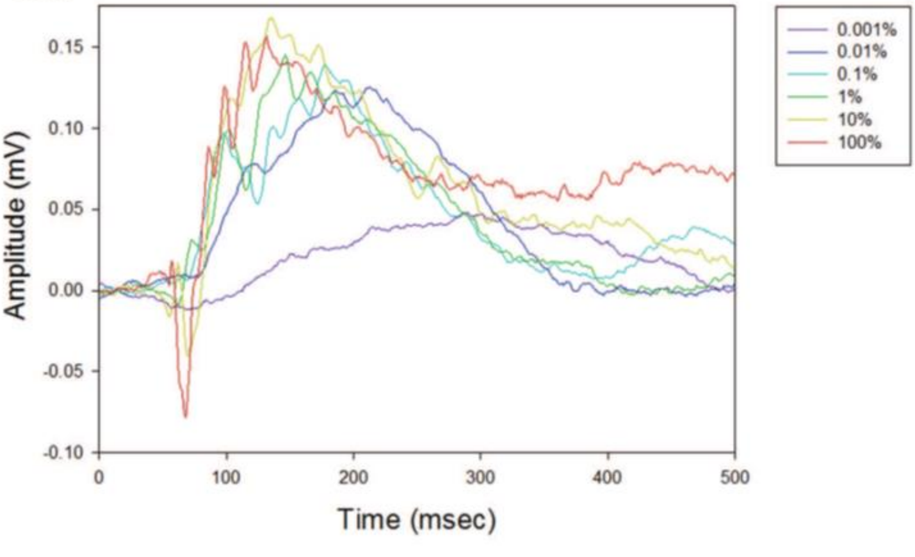

Figure S4: Three representative VEP responses in uninjured eyes. Three examples of uninjured nerves VEP responses to full-light intensity series.

INJURED NOT-TREATED

I.

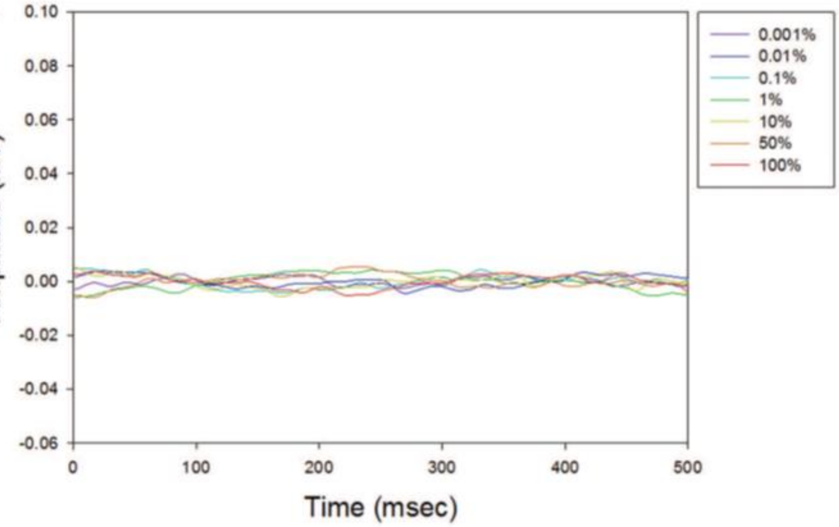

II.

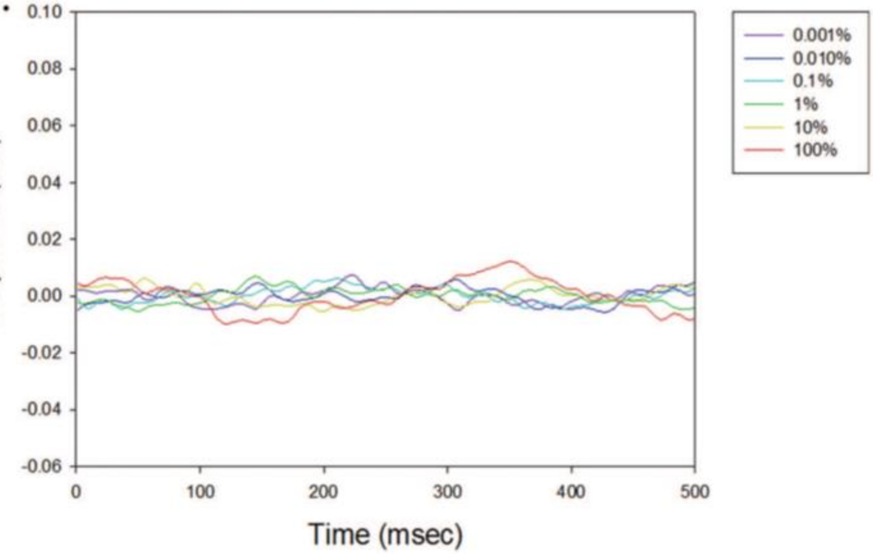

III.

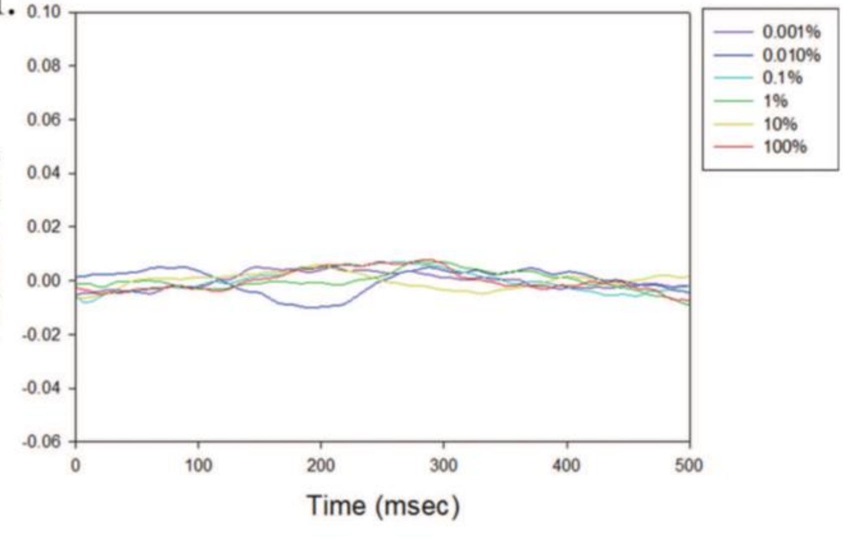

Figure S5: Three representative VEP responses in injured eyes not treated. Three examples of injured nerves with no treatment VEP responses to full-light intensity series.

INJURED WITH FOUR-DRUG TREATMENT

I.

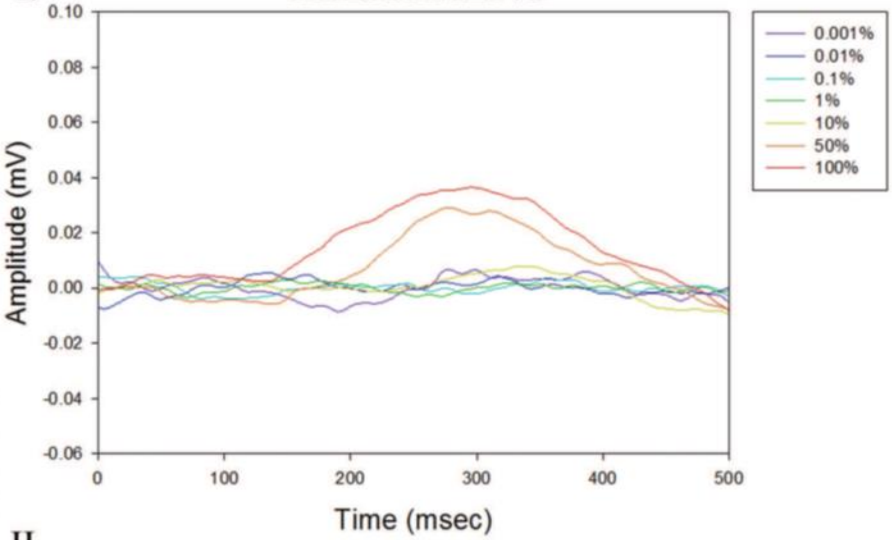

II.

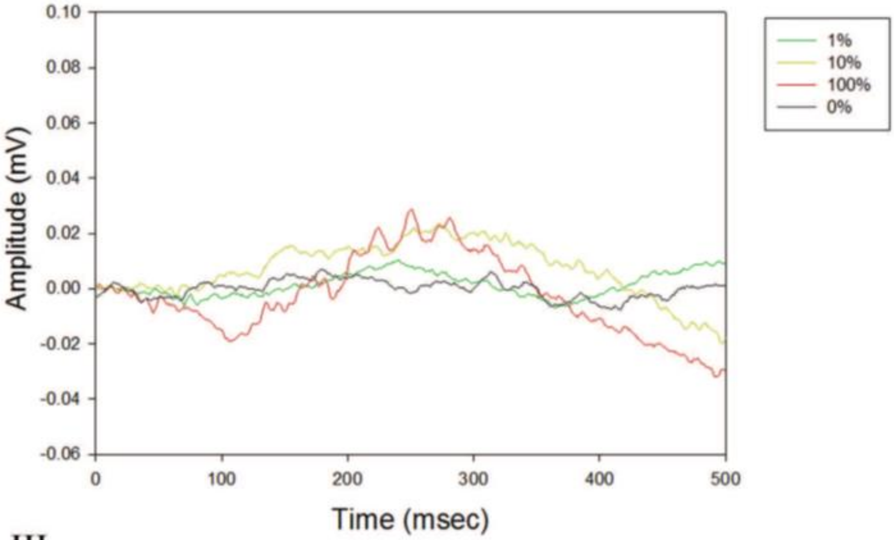

III.

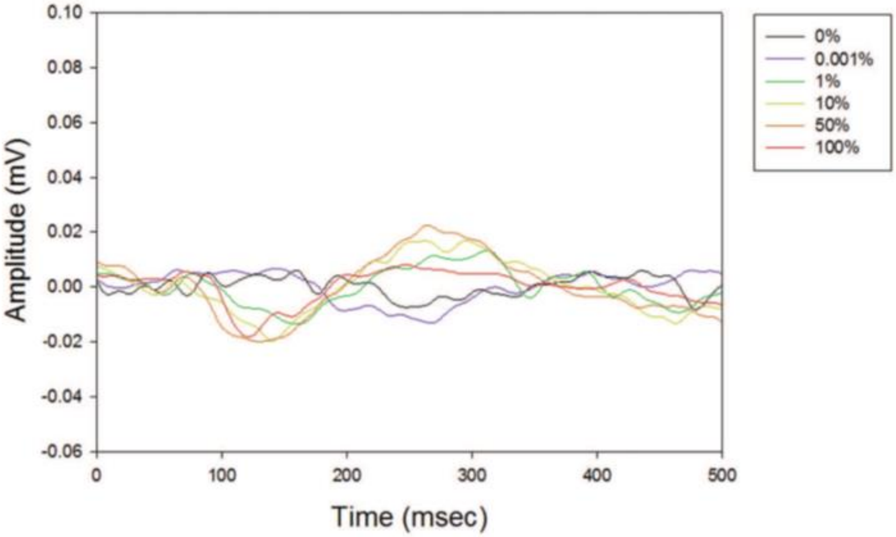

Figure S6: 4-drug treatment partially restores VEP responses. Three examples of injured nerves with four-drug treatment VEP responses to full-light intensity series.

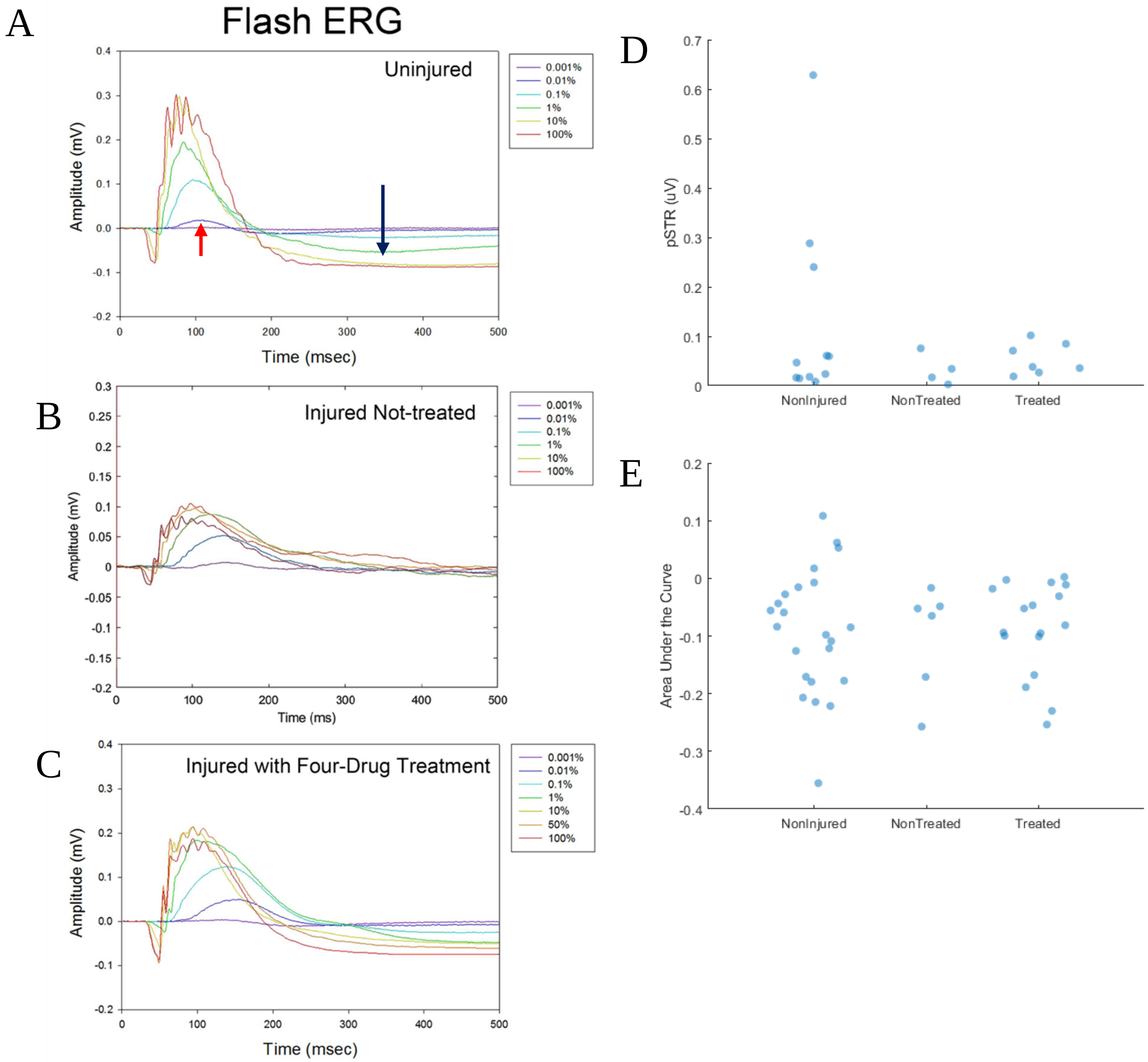

A
Flash ERG
Uninjured
Amplitude (mV)
Time (msec)
0.001%
0.01%
0.1%
1%
10%
100%
B
Injured Not-treated
Amplitude (mV)
Time (ms)
C
Injured with Four-Drug Treatment
Amplitude (mV)
Time (msec)
50%
D
pSTR (uV)
NonInjured
NonTreated
Treated
E
Area Under the Curve

Figure S7 – Four-drug combination effect on the fERG. Two weeks after optic crushes, We performed flash electroretinograms (fERG), which is a strong response to the natural stimuli of light in a graded series of intensities (0.001 to 100% max flash intensity). Representative panels of flash electroretinograms (fERG) traces in response to full light spectrum: (A) Uninjured, (B) Injured Not-treated, and (C) Injured with four-drug treatment. (D) The positive scotopic threshold response (pSTR, red arrow), which is considered a measure of retinal ganglion cell health, was quantified, but no significant changes were found between uninjured eyes and injured eyes, regardless of treatment. (E) The area under the PhNR, another measure of ganglion cell health, was quantified as the area under the curve of the ERG from 0.25 s to 0.5 s and a one-tailed independent student t-test performed. Again, there were no significant changes between groups.

4-drug treatment individual slices displaying torturous pattern of axonal regeneration

A. Z-Slice #240 out of 537 slices

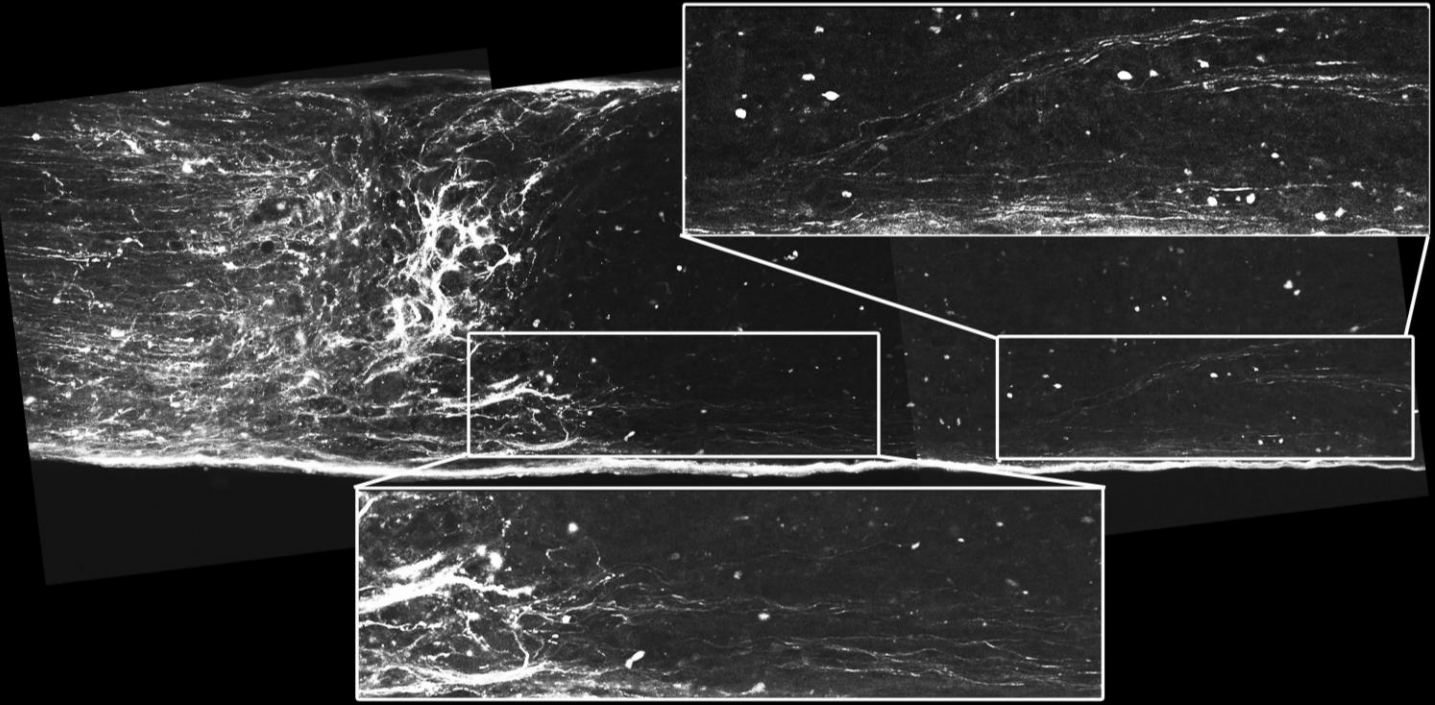

B. Z-Slice #274 out of 537 slices

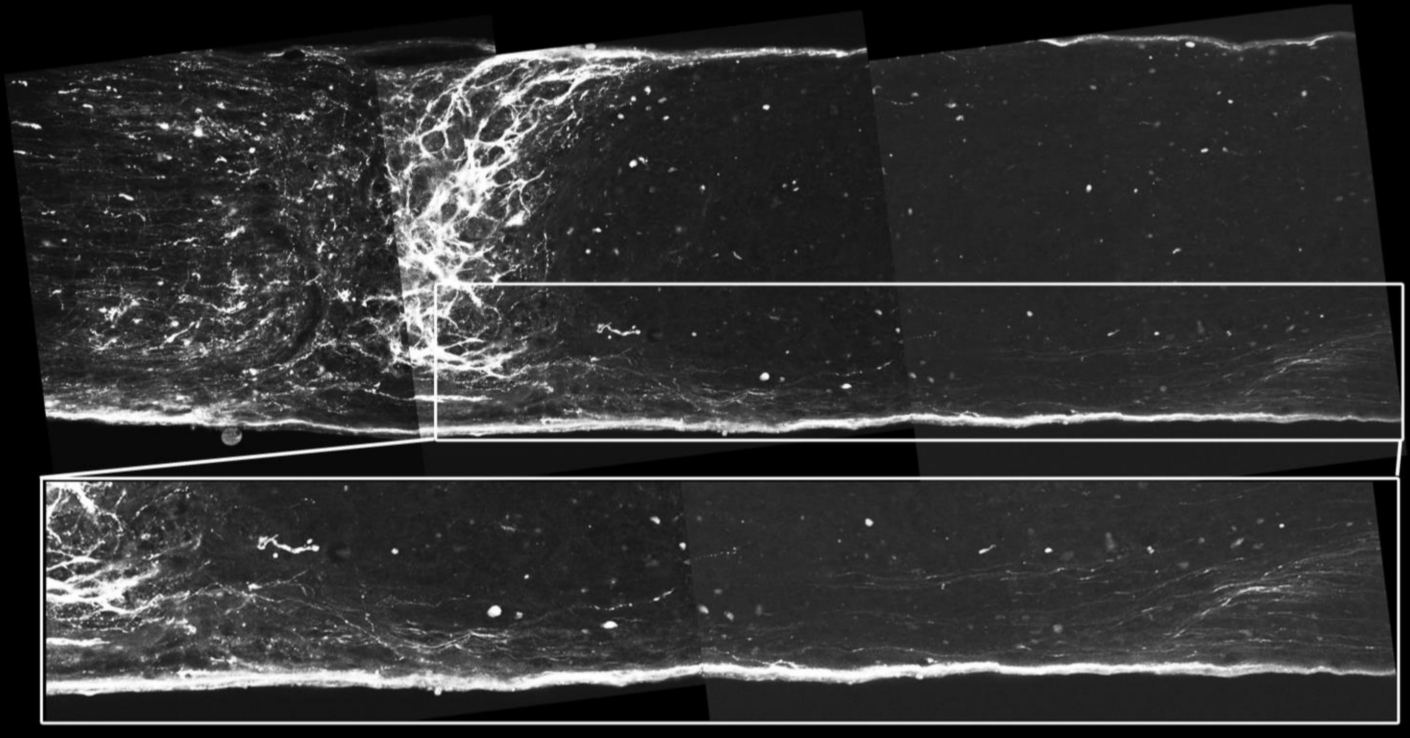

Figure S8: Individual Z-slices of ONC with 4-drug treatment reveal a torturous pattern of axonal regeneration. The image in Figures 2-II & III are a 2-D projection after 3-DISCO clearing of nearly 540 Z-slices, to reveal the pattern of CTB-labeled axons within the whole nerve bundle. Looking at individual Z-slices of this same samples, towards the center of the nerve we look at section #240 out of 537 (A.) and #274 out of 537 (B.). These individual slices or planes of the nerve reveal the CTB-labeled crush site and torturous growth at the crush site and about 1mm beyond that. These two images are just two slices out of the 537, that are used to generate the images in Figures 2-II & III.

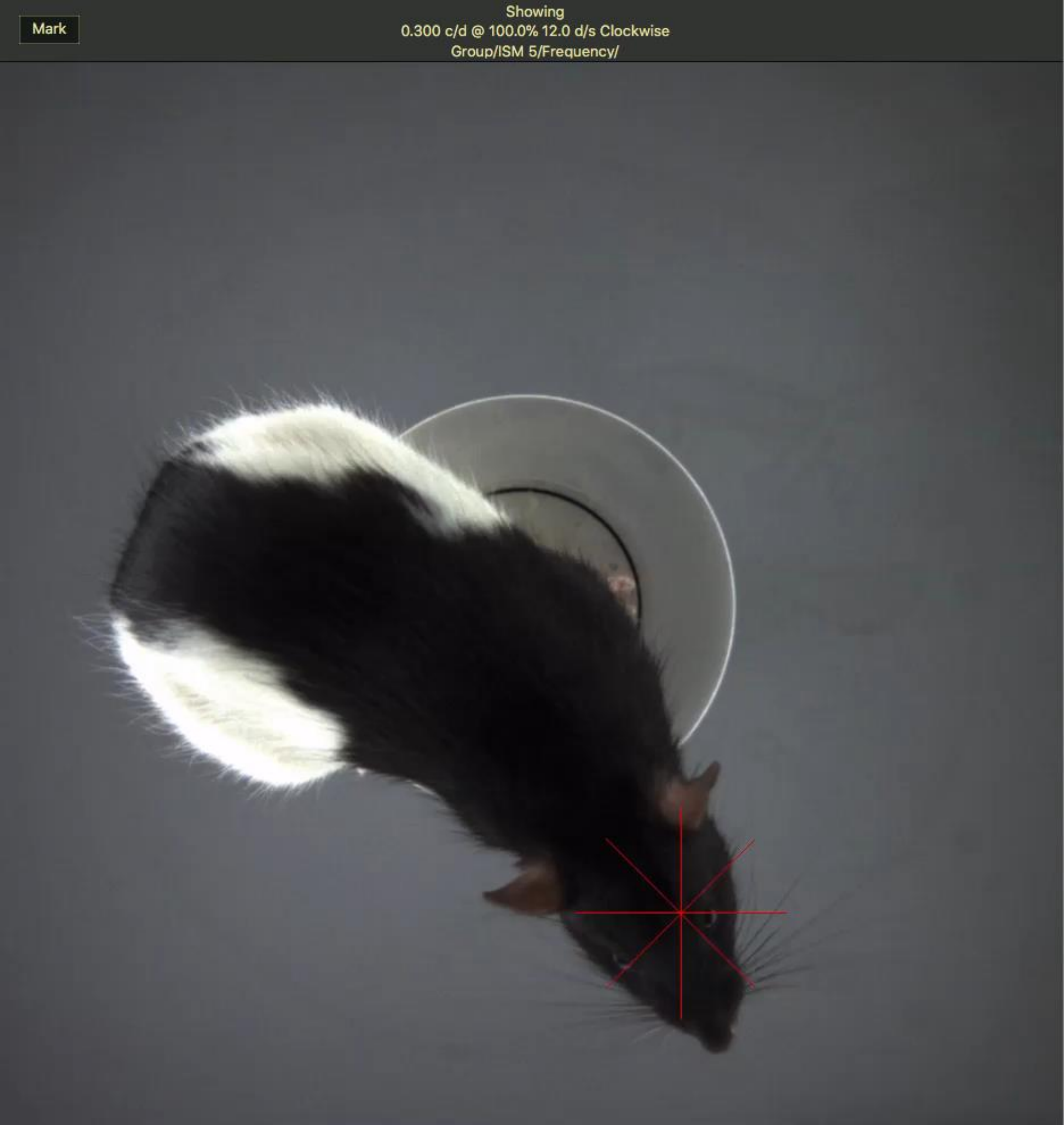
Mark
Showing
0.300 c/d @ 100.0% 12.0 d/s Clockwise
Group/ISM 5/Frequency/

Video 1: Example Optokinetic Response. Animals were placed into the OptoMetry system and presented with drifting visual stimuli. Tracking from both the left and right eye was recorded. This animal can be seen tracking the stimulus at 34 s.

Computational model of Drug Action

The extensive regeneration obtained by the four-drug combination raises the question of how might the drug combination observed effects. For this we used the top down version of the neurite-outgrowth multicompartment ODE model we have developed (18). This model analyzes the dynamics of a whole cell process in terms of interactions between three sub cellular processes *Production of Membrane Components, Vesicle Transport and Exocytosis* and *Microtubule Growth.* This model is built on the microtubule growth model developed by Margolin et al. (31) with the vesicle transport model by Heinrich and Rapaport (32). We used this model of neurite outgrowth as a surrogate for regeneration of the injured axon.

Basic model

This model is an extension of the previous model by Yadaw et al (18). A key difference between this model and the previous one is the number of compartments. Here we use six compartments as this needed to account for the action of drugs. The six compartment include two terminal compartments Trans Golgi Network (TGN), growth cone plasma membrane (GC-PM) and four intermediate compartments (cell body cytoplasm (CBC), neurite shaft cytoplasm proximal (NSC-P), neurite shaft cytoplasm distal (NSC-D), growth cone plasma membrane (GCC). The dynamics of the model starts with production of membrane components at TGN and followed by coat protein B mediated vesicles budding from TGN into cell body cytoplasm, coat protein B containing vesicles are anterograde moving vesicles which are actively transported from cell body cytoplasm to growth cone cytoplasm by the kinesin motor protein. Retrograde (endocytosed) vesicles movement process starts with coat protein A containing vesicles budding at growth cone plasma membrane and it is actively transported to TGN by dynein motor protein. When anterograde and retrograde vesicles reach to target compartments GC-PM and TGN respectively then vesicles tether to the target compartments by interactions between vesicle-SNARE and target-SNAREs and this is PLOS followed by fusion with target compartment. Kinesin mediated anterograde moving vesicles have different binding rates in intermediate compartments because the microtubule associated protein tau is distributed as a gradient increasing from CBC to GCC where Kinesin competes with tau for microtubule binding sites (37-43). This results in presence of a reservoir of membrane vesicles in the distal neurite shaft near the growth cone. Using these specifications, we determined the size of reservoir under various drug treatment conditions. The size of the membrane reservoir near the growth cone would be indicative of the capability of axonal growth in response to treatment with different drugs and the four -drug combination. As the reservoir is depleted more membrane is added to the growth cone allowing the neurite to grow longer. Some but not all of the kinetic parameters are known. Consequently, we used an analytical approach to calculate the remaining parameter when an overall experimentally measured constrained such as velocity of axonal outgrowth is measured. The validity of this approach was established by experimental validation of computational predictions for the formation of dystrophic bulbs and velocity of neurite outgrowth Arjun's Paper,). The overall model used for the calculation of kinetic parameters from the analytical solution is shown below

Model of Drug Action on Axonal Regeneration

Since HU-210 and IL-6 stimulate transcription, we have assumed that this stimulation leads to biosynthesis of molecules needed for axon regeneration at sufficient levels at all times. Based on this assumption the levels of all components are buffered in the model when the cell body is stimulated by HU-210 + IL-6.

The experimentally observed velocities of axonal growth for different drugs and drug combinations were used as a constraint for our model to calculate required membrane fusion at growth cone plasma membrane. The model constraints specified the rate of back-transported membrane from the growth cone plasma membrane to the TGN and the fraction of moving and stationary vesicles in the CBC, NSC-P, NSC-D and GCC compartments (Supp Figure 9) We assumed that endocytosed vesicles should transport 0.5 μm^2 membrane/min back to the TGN based on experiments in the literature (18) and calculated other parameters of the model analytically for a given neurite outgrowth velocity. Velocities of growth were obtained from the morphology experiments. We determined these rates based upon in vivo axonal regeneration, where we graphed the Intensity of Fluorescent signal of CTB over distance (µm) from the crush site and measured average growth with the indicated combinations or single drug over a 3 weeks period. They then divided the total average length of growth by 21days and determined the average velocities. In Supplementary Figures 10A&B we show at steady state, the distribution of all membrane proteins (SNAREs, motor proteins and recruitment factors – Coat proteins A (retrograde) & B (anterograde)), membrane (vesicles) and fluxes in the terminal and intermediate compartments used in the model. (Supplementary Figure 10).

Application of taxol and APC in gel foam at the site of injury has two effects. Taxol increases stabilization of the dynamic microtubule thus enabling microtubule elongation. APC clears extracellular inhibitory environment at injury site which can increase intracellular vesicles mobility from vesicles reservoir NSC-D to growth cone plasma membrane (GC-PM). Experimentally HU-210 and IL-6 applied at cell body allow neurite outgrowth with a velocity v = 0.5 µm/h. When Taxol along with HU-210 + IL-6 is applied, neurites grow at 1 µm/h. Although Taxol stabilizes dynamic microtubules and we should achieve faster growth rates, overall growth of microtubule bundle inhibited by external debris and hence it cannot grow faster despite having fast growing capacity. It is known that when growing microtubules hit, the membrane they collapse (44). In case of APC application at injury site, neurite outgrowth shows a velocity v = 2 µm/h because APC clears inhibitory environment, hence vesicles can move from vesicle reservoir NSC-D to GCC and fuse with growth cone plasma membrane (0.6052 $\frac{\mu m^2}{min}, calculated\ analytically$). However, since there is insufficient microtubule elongation the neurite does not grow very fast.

Analytical solutions (equations 1-158 shown below) for the model identified how the four drug combination could allow axons to grow with a velocity v = 16 µm/h. Depletion of the membrane vesicle pool in the NSC-D compartment allows for the expansion of the growth cone plasma membrane which in turn enables the microtubule's to elongate without inhibition. . A three-

dimensional plot relating axon length to apparent GTPase activity of the microtubules and concentrations of vesicles in the NSC-D compartment is shown in. XA in the main paper The calculated values for the vesicles levels in the NSC-D compartment under the various drug conditions are shown in and corresponding rates of microtubule elongation and axon length are shown in XC and XD in the main paper. The profile of the plots indicate that the seemingly synergistic effects of the four drug combination can be explained parsimoniously by the action of APC, which by removing the extracellular inhibitory environment allows for vesicle movement from the NCS-D compartment to the GC-PM compartment. This increase in the plasma membrane at the e growth cone coordinated with the enhanced growth rate of the microtubule due to Taxol dependent stabilization of the dynamic microtubules allows for the observed overall growth rate of the axon. Overall our model indicates that relief of inhibition of growth cone plasma membrane expansion is sufficient to account for the observed experimental growth rates.

Analytical Solution

The development of the analytical solution is extension and rearrangement of previously developed model (18). Here, we assumed initial forward transport rate at TGN is constant because HU-210 and IL-6 applied at cell body by intravitreal injection increases the capacity of membrane production at TGN so transport is not limited by availability of membrane vesicles. To achieve a specific axonal outgrowth velocity, we calculated fraction of bound kinesin mediate membrane vesicle transport from the reservoir (NSC-D) to GCC. Membrane that is incorporated into vesicles that bud from the TGN can be separated into 4 different membrane vesicle types (I, II, III, IV). Membrane vesicles of the type-I will be added to the growing neurite shaft cytoplasm distal (NSC-D) as part of the neurite shaft cytoplasm reservoir for anterograde moving vesicles where NSC-D work as a reservoir next to growth cone which can be mobilized on sudden demand for membrane at growth cone. Type-II membrane vesicles will be added to the GC and from there to the growing neurite shaft. Type-III and type-IV membrane vesicles will also be added to the GC, but the fused membrane will then be incorporated into endocytic retrograde vesicles. Similar to first membrane vesicle set, the third vesicle set will be added to the growing neurite shaft cytoplasm, though this set is much smaller due to an assumed nine-fold lower amount of stationary retrograde vesicles in the neurite shaft cytoplasm. The fourth membrane vesicle set will be back transported to the TGN, i.e. this set constitutes the cycling membrane between the TGN and the GC. As a simplification, we assume in our solution that membranes transported in an anterograde manner is only transported as vesicles that bud from the TGN with coat protein B and retrogradely transported membranes are vesicles that bud from the GC with coat protein A.

The microtubule dynamics model depends on dynamic microtubule profile, nucleation rate, degradation rate, stabilization rate and hydrolysis rates. We used Margolin model (31) for simulation of dynamic microtubules for fixed effective tubulin concentration and different hydrolysis rates (0.60 sec^{-1} – 0.80 sec^{-1}). To calculate the microtubule bundle length in axonal shaft, we calculated the length of dynamic and stable microtubules and divided by the number of microtubules in cross section. Dynamic microtubules have a stochastic profile, which changes as they undergo catastrophic disassembly by hydrolysis of GTP bound tubulins at tip. Application of

taxol and APC in gel foam at the site of injury create four different criteria of GTP hydrolysis rate of dynamic microtubules. We call (i) ‘apparent base hydrolysis rate’ in absence of (Taxol & APC) where catastrophic disassembly of dynamic microtubules happens due to GTP hydrolysis as well as external inhibitory signal at crush site; (ii) ‘apparent hydrolysis rate under taxol’ when taxol present at injury site which reduce GTP hydrolysis rate but not external inhibitory signal, (iii) ‘base hydrolysis rate’ when APC alone is present at injury site, (iv) ‘hydrolysis rate under taxol’ when (Taxol & APC) both drugs are present at injury site, in this case taxol reduces hydrolysis rate. We found ascending order relationship in (i) > (ii) > (iii) > (iv) for these four conditions.

For an experimentally observed axonal outgrowth velocity, the amount of cycling membrane vesicles and percentage of stationary anterograde vesicles in (NSC-P & NSC-D) and retrograde vesicles in (NSC-P & NSC-D) allows for the calculation of the fluxes of the four different membrane vesicle types. Since HU210 and IL-6, applied at cell body increases biosynthesis of molecules and membrane vesicles at Trans Golgi Network such that membrane vesicle production is not limiting. We calculated the optimum value of “Initial forward transport rate” which satisfies the growth velocities of all drug combination by satisfying know biological constraints such as (recycling membrane rate, retrograde vesicles motor binding rate with microtubule bundle and mobilization of anterograde vesicles from NSC-D compartment to growth cone plasma membrane with full capacity. We need to produce membrane budding at TGN compartment is 1.4001 $\mu m^2/min$.

$$initial\ forward\ transport\ rate = 1.4001\ \frac{\mu m^2}{min} \quad (1)$$

Type II membrane vesicle transport rate (Final forward transport rate):

$$Growth\ related\ k\ membrane\ production = \frac{Axonal\ outgrowth\ velocity}{2\pi\ \times radius\ of\ neurite}\ . \quad (2)$$

Type IV membrane vesicle transport rate (Final Backward transport rate):

$$final\ back\ transport\ rate = cycling\ rate\ , \quad (3)$$

$$a2\ travel\ time\ new\ NSCP = \frac{Axonal\ outgrowth\ velocity}{v_d} \quad (4)$$

$$a2\ travel\ time\ new\ NSCD = \frac{Axonal\ outgrowth\ velocity}{v_d} \quad (5)$$

$$rate\ lost\ na2\ NSCP = \frac{final\ backward\ transport\ rate \times a2\ travel\ time\ new\ NSCP}{anticipated\ fraction\ of\ bound\ a2\ vesicles\ in\ NSCP} \quad (6)$$

$$rate\ lost\ na2\ NSCD = \frac{final\ backward\ transport\ rate \times a2\ travel\ time\ new\ NSCD}{anticipated\ fraction\ of\ bound\ a2\ vesicles\ in\ NSCD} \quad (7)$$

Type III membrane vesicle transport rate (Initial back transport rate):

$$\begin{aligned}&initial\ back\ transport\ rate\\&\qquad = final\ backward\ transport\ rate\\&\qquad + \ rate\ of\ accumulation\ of\ stationary\ na_2\ NSCP\\&\qquad + \ rate\ of\ accumulation\ of\ stationary\ na_2\ NSCD\end{aligned} \tag{8}$$

$$\begin{aligned}&Final\ forward\ transport\ rate = initial\ back\ transport\ rate\ +\\&\qquad Growth\ related\ k\ membrane\ production\end{aligned} \tag{9}$$

$$b_1\ travel\ time\ new\ NSCP = \frac{Axonal\ outgrowth\ velocity}{v_k} \tag{10}$$

$$b_1\ travel\ time\ new\ NSCD = \frac{Axonal\ outgrowth\ velocity}{v_k} \tag{11}$$

$$\begin{aligned}&rate\ of\ accomulation\ of\ stationary\ vesicles\ (surface\ area)\ nb_1 NSCP\\&\qquad = Initial\ forward\ transport\ rate \times b_1\ travel\ time\ new\ NSCP\\&\qquad \times fraction\ of\ bound\ b_1\ vesicles\ in\ NSCP\end{aligned} \tag{12}$$

$$\begin{aligned}&rate\ of\ accomulation\ of\ stationary\ vesicles\ (surface\ area)\ nb_1 NSC\mathrm{D}\\&= Initial\ forward\ transport\ rate - Final\ forward\ transport\ rate\\&- rate\ of\ accomulation\ of\ stationary\ vesicles\ (surface\ area)\ nb_1 NSCP\end{aligned} \tag{13}$$

$$\begin{aligned}&fraction\ of\ bound\ b_1 vesicles\ in\ NSC\mathrm{D}\\&= \frac{Final\ forward\ transport\ rate \times b_1 travel\ time\ new\ NSCD}{rate\ of\ accomulation\ of\ stationary\ vesicles\ (surface\ area)\ nb_1 NSC\mathrm{D}}\end{aligned} \tag{14}$$

Type I membrane vesicle transport rate (Initial forward transport rate):

$$\begin{aligned}&rate\ of\ accomulation\ of\ stationary\ vesicles (surface\ area) in\ NSC\\&\qquad = rate\ of\ accumulation\ of\ stationary\ vesicles\ (surface\ area)\ in\ NSCP\\&\qquad + rate\ of\ accumulation\ of\ stationary\ vesicles\ (surface\ area) in\ NSCD\\&\qquad = (rate\ of\ accumulation\ of\ stationary\ na_2\ in\ NSCP\\&\qquad + \ rate\ of\ accumulation\ of\ stationary\ nb_1\ in\ NSCP)\\&\qquad + (rate\ of\ accumulation\ of\ stationary\ na_2\ in\ NSCD\\&\qquad + \ rate\ of\ accumulation\ of\ stationary\ nb_1\ in\ NSCD)\end{aligned} \tag{15}$$

$$w_G^B = \frac{initial\ forward\ transport\ rate}{cc1_G \times s_G}, \quad (16)$$

$$w_{PM}^A = \frac{initial\ backward\ transport\ rate}{cc2_{PM} \times s_{PM}}, \quad (17)$$

$$w_{PM}^B = w_G^B \times factor\ for\ site\ specific\ budding, \quad (18)$$

$$w_G^A = w_{PM}^A \times factor\ for\ site\ specific\ budding. \quad (19)$$

The fluxes also allowed us to calculate the initial membrane surface areas in the cytoplasmic compartments.

$$b_1\ travel\ time\ NSCP\ at\ start = \frac{NSCP\ length\ at\ start}{v_k}, \quad (20)$$

$$b_1\ travel\ time\ NSCD\ at\ start = \frac{NSCD\ length\ at\ start}{v_k}, \quad (21)$$

$$n_{MTCP}^{B_G} = \frac{final\ forward\ transport\ rate\ \times\ b_1\ travel\ time\ NSCP\ at\ start}{anticipated\ fraction\ of\ bound\ b_1\ vesicles\ in\ NSCP}, \quad (22)$$

$$n_{MTCD}^{B_G} = \frac{final\ forward\ transport\ rate\ \times b_1\ travel\ time\ NSCD\ at\ start}{anticipated\ fraction\ of\ bound\ b1\ vesicles\ in\ NSCD}, \quad (23)$$

$$n_{GCC}^{B_G} = \frac{final\ forward\ transport\ rate}{anticipated\ fraction\ of\ fusing\ b_1\ \ vesicles\ in\ GCC}, \quad (24)$$

$$a_2\ travel\ time\ NSCP\ at\ start = \frac{NSCP\ length\ at\ start}{v_d}, \quad (25)$$

$$a_2\ travel\ time\ NSCD\ at\ start = \frac{NSCD\ length\ at\ start}{v_d}, \quad (26)$$

$$n_{NSCP}^{A_{PM}} = \frac{final\ backword\ transport\ rate\ \times\ a_2\ travel\ time\ NSCP\ at\ start}{anticipated\ fraction\ of\ bound\ a_2\ vesicles\ in\ NSCP}, \quad (27)$$

$$n_{NSCD}^{A_{PM}} = \frac{final\ backword\ transport\ rate\ \times\ a_2\ travel\ time\ NSCD\ at\ start}{anticipated\ fraction\ of\ bound\ a_2\ vesicles\ in\ NSCD}, \quad (28)$$

$$n_{CBC}^{A_{PM}} = \frac{final\ backword\ transport\ rate}{anticipated\ fraction\ of\ fusion\ a_2\ vesicles\ in\ CBC}. \quad (29)$$

Similarly, to the fraction of anterograde vesicles that are bound to the microtubule and therefore moving in the neurite shaft cytoplasm, we define the fractions of moving anterograde vesicles in the GC cytoplasm (i.e. vesicles that fuse with the GC membrane) and moving retrograde vesicles in the CB cytoplasm (i.e. vesicles that fuse with the TGN). The anticipated membrane fusion rate at the growth cone (or final forward transport rate) is equal to the sum of the fluxes of the second, third and fourth membrane types, the anticipated membrane fusion rate at the TGN (or final backward transport rate) is equal to the flux of the fourth membrane type. This allows the calculation of the number of v-SNAREs that are associated with each set of vesicles in both cytoplasmic compartments. We want to estimate the amount of v-SNAREs V in the GC cytoplasm or U in the CB cytoplasm that enables dynamic balance For this the fractions of fusing vesicles should equal the anticipated fractions delivered to the compartment and that the total membrane fluxes equal the final forward (type II) and final backward (Type IV) membrane fluxes for a predefined amount of t-SNAREs Y at the TGN and X at the GC for specified tethering rates tethering rates.

$$kappaXU_{pm} = kappaXU_g \times factor\ for\ site\ specific\ SNARE\ complex\ formation\ , \quad (30)$$

$$kappaXU_g = kappaXU_{pm} \times factor\ for\ site\ specific\ SNARE\ complex\ formation\ , \quad (31)$$

$$YV\ SNARE\ complexes\ GCC\ PM = \frac{final\ forward\ transport\ rate}{anterograde\ vesicle\ surface\ area} \times SNARE\ complex\ per\ vesicle\ fusion\ , \quad (32)$$

$$VV_{GCC}^{B_G} = \frac{YV\ SNARE\ complexes\ GCC\ PM}{kappaYV_{pm}} \times YY_{pm}\ , \quad (33)$$

$$V_{GCC}^{B_G} = \frac{VV_{GCC}^{B_G}}{n_{GCC}^{B_G}}\ . \quad (34)$$

Vesicles that fuse with the TGN or the GC transmit their SNAREs to the target organelle. Consequently, the vesicles that enter the CB cytoplasm or GC cytoplasm from the NSC should contain the same number of SNAREs to keep the number of SNARE molecules in the cytoplasmic compartments constant. Additionally, v-SNAREs need to be back transported to the GC or the TGN to be available for the next set of budding vesicles. The fluxes for each v-SNARE can be associated to 4 different types in a similar manner as the membrane fluxes. Initial and final forward and backward fluxes can be calculated accordingly.

Similarly, to the v-SNAREs V, we calculate the corresponding parameter for v-SNARE U.

$$XU\ SNARE\ complexes\ CBC\ G = \frac{final\ backtransport\ rate}{anterograde\ vesicle\ surface\ area} \times SNARE\ complex\ per\ vesicle\ fusion\ , \quad (35)$$

$$UU_{CBC}^{A_{PM}} = \frac{XU\ SNARE\ complexes\ CBC\ G}{kappaXU_G} \times XX_G\ , \quad (36)$$

$$U_{CBC}^{A_{PM}} = \frac{UU_{CBC}^{A_{PM}}}{n_{CBC}^{A_{PM}}}\,, \tag{37}$$

$$U\_backward = U_{CBC}^{A_{PM}}\,, \tag{38}$$

$$UU\ final\ backward = U\_backward \times final\ backtransport\ rate, \tag{39}$$

$$UU\ initial\ backward = U\_backward \times initial\ backtransport\ rate, \tag{40}$$

$$UU\ final\ forward = UU\ Initial\ backward\,, \tag{41}$$

$$U_{backward} = \frac{UU\ final\ forward}{final\ forward\ transport\ rate}\,, \tag{42}$$

$$UU\ initial\ forward = initial\ forward\ transport\ rate\ \times\ U\_forward. \tag{43}$$

SNAREs are recruited into the budding vesicles in a competitive manner, i.e. all four SNAREs compete with each other for the SNARE binding spots of the budding vesicles. The amounts of t-SNAREs Y and X at the GC and the TGN are predefined. Considering this we calculated the amount of v-SNAREs V and U at the TGN and GC that allow the calculated initial forward and backward v-SNARE fluxes.

$$Y_{PM} = \frac{YY_{PM}}{s_{PM}}, \tag{44}$$

$$X_{PM} = kxa\ \times \frac{Y_{PM}}{kya}, \tag{45}$$

$$cont_{PM} = 1 + \frac{Y_{PM}}{kya} + \frac{X_{PM}}{kxa}, \tag{46}$$

$$bs = initial\ backtransport\ rate\ \times snare\ binding\ site\ per\ vesicle\ area\,, \tag{47}$$

$$U_{PM} = \frac{UU\ initial\ backward\ \times cont_{PM} \times kua}{\big(bs - (UU\ initial\ backward + VV\ initial\ backward\,)\big)}\,, \tag{48}$$

$$V_{PM} = \frac{VV\ initial\ backward\ \times cont_{PM} \times kva}{\big(bs - (UU\ initial\ backward + VV\ initial\ backward\,)\big)}\,, \tag{49}$$

$$UU_{PM} = U_{PM}\ \times s_{PM}\,, \tag{50}$$

$$XX_{PM} = X_{PM}\ \times s_{PM}. \tag{51}$$

Though t-SNAREs have a higher dissociation constant for association with the vesicles than v-SNAREs they still are transported between the TGN and GC. We calculate their transport rates in a similar manner as for the vesicle SNAREs.

$$snare\ saturation\ denom\ 2a = 1 + \frac{X_{PM}}{kxa} + \frac{U_{PM}}{kua} + \frac{Y_{PM}}{kya} + \frac{V_{PM}}{kva}, \quad (52)$$

$$sya2 = \frac{snare\ binding\ site\ per\ vesicle\ area \times \frac{Y_{PM}}{kya}}{snare\ saturation\ denom\ 2a}, \quad (53)$$

$$YY\ initial\ backward = sya2 \times initial\ backtransport\ rate, \quad (54)$$

$$YY\ final\ backward = sya2 \times final\ backtransport\ rate, \quad (55)$$

$$Y\ initial\ backward = \frac{YY\ initial\ backward}{initial\ backtransport\ rate}, \quad (56)$$

$$YY\ final\ forward = YY\ initial\ backward,, \quad (57)$$

$$Y\ forward = \frac{YY\ final\ forward}{final\ forward\ transport\ rate}, \quad (58)$$

$$Y\ initial\ forward = Y\ forward \times initial\ forward\ transport\ rate\ ., \quad (59)$$

The 'consumption' of membrane proteins that are associated with the vesicles that are added to the NSC reservoir demands the continuous production of membrane proteins at the TGN. The protein production rates can be calculated by considering the concentration of each protein at anterograde and retrograde moving vesicles and the amount of membrane that will be added to the NSC reservoir as shown for the SNAREs V and Y.

$$\begin{aligned} k\ VV\ production &= (rate\ of\ accumulation\ of\ stationary\ nb_1\ NSCP \times V\ forward \\ &+ rate\ of\ accumulation\ of\ stationary\ na_2\ NSCP \times V\ backward) \\ &+ (rate\ of\ accumulation\ of\ stationary\ nb_1\ NSCD \times V\ forward \\ &+ rate\ of\ accumulation\ of\ stationary\ na_2\ NSCD \times V\ backward) \end{aligned} \quad (60)$$

$$\begin{aligned} k\ YY\ production &= (rate\ of\ accumulation\ of\ stationary\ nb_1\ NSCP \times Y\ forward \\ &+ rate\ of\ accumulation\ of\ stationary\ na_2\ NSCP \times Y\ backward) \\ &+ (rate\ of\ accumulation\ of\ stationary\ nb_1\ NSCD \times Y\ forward \\ &+ rate\ of\ accumulation\ of\ stationary\ na_2\ NSCD \times Y\ backward) \end{aligned} \quad (61)$$

The concentration of the t-SNARE Y at the TGN is calculated via the following equations.

$$X_G = \frac{XX_G}{s_G}, \quad (62)$$

$$YY_G = \max\left(0, Y\ forward \times \frac{X_G}{kxb} \times kyb \times s_G - k\ YY\ production\right). \quad (63)$$

TGN concentrations for the v-SNARE V and U are calculated in a similar manner as their concentrations at the GC.

t-SNARE X movements are calculated similarly as t-SNARE Y movements.

$$snare\ saturation\ denom\ 1b = 1 + \frac{X_G}{kxb} + \frac{U_G}{kub} + \frac{Y_G}{kyb} + \frac{V_G}{kvb}\ , \tag{64}$$

$$sxb1 = \frac{snare\ binding\ spot\ per\ vesicle\ area \times \frac{X_G}{kxb}}{snare\ saturation\ denom\ 1b}\ , \tag{65}$$

$$X\ forward = \ sxb1\ , \tag{66}$$

$$XX\ initial\ forward = X\ forward \times initial\ forward\ transport\ rate\ , \tag{67}$$

$$XX\ final\ forward = X\ forward \times final\ forward\ transport\ rate\ , \tag{68}$$

$$XX\ initial\ backward = XX\ final\ forward\ , \tag{69}$$

$$X\ backward = \frac{XX\ initial\ forward}{initial\ backward\ transport\ rate}\ , \tag{70}$$

$$XX\ final\ backward = X\ backward \times final\ backward\ transport\ rate\ . \tag{71}$$

Production rates for SNAREs U and X are calculated.

SNARE X, U and V protein amounts at the TGN are calculated:

$$\begin{aligned} k\ UU\ production \\ &= (rate\ of\ accumulation\ of\ stationary\ nb_1\ NSCP \times U\ forward \\ &+ \ rate\ of\ accumulation\ of\ stationary\ na_2\ NSCP \times U\ backward) \\ &+ (rate\ of\ accumulation\ of\ stationary\ nb_1\ NSCD \times U\ forward \\ &+ rate\ of\ accumulation\ of\ stationary\ na_2\ NSCD\ \times U\ backward) \end{aligned} \tag{72}$$

$$\begin{aligned} k\ XX\ production \\ &= (rate\ of\ accumulation\ of\ stationary\ nb_1\ NSCP \times X\ forward \\ &+ \ rate\ of\ accumulation\ of\ stationary\ na_2\ NSCP \times X\ backward) \\ &+ (rate\ of\ accumulation\ of\ stationary\ nb_1\ NSCD \times X\ forward \\ &+ rate\ of\ accumulation\ of\ stationary\ na_2\ NSCD\ \times X\ backward) \end{aligned} \tag{73}$$

Protein for all vesicle membrane proteins in the different compartments are calculated:

$$UU_G = \max(0, (UU_G - k\ UU\ production)), \tag{74}$$

$$XX_G = \max(0, (XX_G - k\ XX\ production))\ , \tag{75}$$

$$VV_G = \max(0, (VV_G - k\ VV\ production)). \tag{76}$$

We calculated the amount of motor protein receptors that are associated with anterograde and retrograde vesicles in the neurite shaft cytoplasm that is necessary to allow the anticipated fraction of microtubule bound (i.e. moving) vesicles. This enabled the calculation of the motor protein receptors in the other cytoplasmic compartments and the amount of motor protein receptors at the TGN and GC as well as their production rates in a similar manner as we did it for the v-SNAREs.

$$\left.\begin{aligned} VV_{CBC}^{B_G} &= n_{CBC}^{B_G} \times V\,forward \\ VV_{NSC}^{B_G} &= n_{NSC}^{B_G} \times V\,forward \\ VV_{GCC}^{B_G} &= n_{GCC}^{B_G} \times V\,forward \\ VV_{GCC}^{A_{PM}} &= n_{GCC}^{A_{PM}} \times V\,backward \\ VV_{NSCP}^{A_{PM}} &= n_{NSCP}^{A_{PM}} \times V\,backward \\ VV_{NSCD}^{A_{PM}} &= n_{NSCD}^{A_{PM}} \times V\,backward \\ VV_{CBC}^{A_{PM}} &= n_{CBC}^{A_{PM}} \times V\,backward \end{aligned}\right\}, \tag{77}$$

$$\left.\begin{aligned} UU_{CBC}^{B_G} &= n_{CBC}^{B_G} \times U\,forward \\ UU_{NSC}^{B_G} &= n_{NSC}^{B_G} \times U\,forward \\ UU_{GCC}^{B_G} &= n_{GCC}^{B_G} \times U\,forward \\ UU_{GCC}^{A_{PM}} &= n_{GCC}^{A_{PM}} \times U\,backward \\ UU_{NSCP}^{A_{PM}} &= n_{NSCP}^{A_{PM}} \times U\,backward \\ UU_{NSCD}^{A_{PM}} &= n_{NSCD}^{A_{PM}} \times U\,backward \\ UU_{CBC}^{A_{PM}} &= n_{CBC}^{A_{PM}} \times U\,backward \end{aligned}\right\}, \tag{78}$$

$$\left.\begin{aligned} XX_{CBC}^{B_G} &= n_{CBC}^{B_G} \times X\,forward \\ XX_{NSC}^{B_G} &= n_{NSC}^{B_G} \times X\,forward \\ XX_{GCC}^{B_G} &= n_{GCC}^{B_G} \times X\,forward \\ XX_{GCC}^{A_{PM}} &= n_{GCC}^{A_{PM}} \times X\,backward \\ XX_{NSCP}^{A_{PM}} &= n_{NSCP}^{A_{PM}} \times X\,backward \\ XX_{NSCD}^{A_{PM}} &= n_{NSCD}^{A_{PM}} \times X\,backward \\ XX_{CBC}^{A_{PM}} &= n_{CBC}^{A_{PM}} \times X\,backward \end{aligned}\right\}, \tag{79}$$

$$\left.\begin{aligned} YY_{CBC}^{B_G} &= n_{CBC}^{B_G} \times Y\ forward \\ YY_{NSC}^{B_G} &= n_{NSC}^{B_G} \times Y\ forward \\ YY_{GCC}^{B_G} &= n_{GCC}^{B_G} \times Y\ forward \\ YY_{GCC}^{A_{PM}} &= n_{GCC}^{A_{PM}} \times Y\ backward \\ YY_{NSCP}^{A_{PM}} &= n_{NSCP}^{A_{PM}} \times Y\ backward \\ YY_{NSCD}^{A_{PM}} &= n_{NSCD}^{A_{PM}} \times Y\ backward \\ YY_{CBC}^{A_{PM}} &= n_{CBC}^{A_{PM}} \times Y\ backward \end{aligned}\right\}. \quad (80)$$

Finally, we calculated the fluxes for the other stationary proteins, i.e. recruitment factors 1 and 2 as well as the initial protein amounts in the different cytoplasmic compartments.

$$KK_{NSCP}^{B_G} = \frac{log(1 - anticipated\ fraction\ of\ bound\ vesicles\ in\ NSCP)}{log(1 - fraction\ bound\ kk\ a1b1\ NSCP)}, \quad (81)$$

$$KK_{NSCD}^{B_G} = \frac{log(1 - anticipated\ fraction\ of\ bound\ vesicles\ in\ NSCD)}{log(1 - fraction\ bound\ kk\ a1b1\ NSCD)}, \quad (82)$$

$$K_{NSCP}^{B_G} = \frac{KK_{NSCP}^{B_G}}{anterograde\ vesicles\ surface\ area}, \quad (83)$$

$$K_{NSCD}^{B_G} = \frac{KK_{NSCD}^{B_G}}{anterograde\ vesicles\ surface\ area}, \quad (84)$$

$$K\ forward = K_{NSCD}^{B_G}, \quad (85)$$

$$KK\ initial\ forward = K\ forward \times initial\ forward\ transport\ rate, \quad (86)$$

$$KK\ final\ forward = K\ forward \times final\ forward\ transport\ rate, \quad (87)$$

$$KK\ initial\ backward = KK\ final\ forward, \quad (88)$$

$$K\ backward = \frac{KK\ initial\ backward}{initial\ backward\ traport\ rate}, \quad (89)$$

$$MBSPVA = motor\ protein\ binding\ sites\ per\ vesicles\ area,$$

$$K_G = \frac{kkb \times KK\ initial\ forward}{initial\ forward\ traport\ rate \times MBSPVA - KK\ initial\ forward}, \quad (90)$$

$$K_{PM} = \frac{kka \times KK\ initial\ backward}{initial\ backward\ traport\ rate \times MBSPVA - KK\ initial\ backward}, \quad (91)$$

$$KK_G = K_G \times s_G, \quad (92)$$

$$KK_{PM} = K_{PM} \times s_{PM}, \quad (93)$$

$$KK_{CBC}^{B_G} = k\ forward \times n_{CBC}^{B_G}, \tag{94}$$

$$KK_{NSC}^{B_G} = k\ forward \times n_{NSC}^{B_G}, \tag{95}$$

$$KK_{GCC}^{B_G} = k\ forward \times n_{GCC}^{B_G}, \tag{96}$$

$$KK_{CBC}^{B_{PM}} = k\ backward \times n_{CBC}^{A_{PM}}, \tag{97}$$

$$KK_{NSC}^{B_{PM}} = k\ backward \times n_{NSC}^{A_{PM}}, \tag{98}$$

$$KK_{GCC}^{B_{PM}} = k\ backward \times n_{GCC}^{A_{PM}}, \tag{99}$$

$$\begin{aligned} k\ KK\ production &= (rate\ of\ accumulation\ of\ stationary\ nb_1\ NSCP \times K\ forward \\ &+ rate\ of\ accumulation\ of\ stationary\ na_2\ NSCP \times K\ backward) \\ &+ (rate\ of\ accumulation\ of\ stationary\ nb_1\ NSCD \times K\ forward \\ &+ rate\ of\ accumulation\ of\ stationary\ na_2\ NSCD \times K\ backward) \end{aligned} \tag{100}$$

$$DD_{NSCP}^{B_G} = \frac{log(1 - anticipated\ fraction\ of\ bound\ vesicles\ in\ NSCP)}{log(1 - fraction\ bound\ kk\ a1b1\ NSCP)}, \tag{101}$$

$$DD_{NSCD}^{B_G} = \frac{log(1 - anticipated\ fraction\ of\ bound\ vesicles\ in\ NSCD)}{log(1 - fraction\ bound\ kk\ a1b1\ NSCD)}, \tag{102}$$

$$D_{NSCP}^{B_G} = \frac{DD_{NSCP}^{B_G}}{anterograde\ vesicles\ surface\ area}, \tag{103}$$

$$D_{NSCD}^{B_G} = \frac{DD_{NSCD}^{B_G}}{anterograde\ vesicles\ surface\ area}, \tag{104}$$

$$D\ backward = DD_{NSC}^{A_{PM}}, \tag{105}$$

$$DD\ initial\ backward = D\ backward \times initial\ backward\ transport\ rate, \tag{106}$$

$$DD\ final\ forward = DD\ initial\ backward, \tag{107}$$

$$DD\ initial\ forward = D\ forward \times initial\ forward\ transport\ rate, \tag{108}$$

$$D_{G=} = \frac{kdb \times DD\ initial\ forward}{initial\ forward\ traport\ rate \times MBSPVA - DD\ initial\ forward}, \tag{109}$$

$$K_{PM} = \frac{kda \times \ DD\ initial\ backward}{initial\ backward\ traport\ rate \times MBSPVA - DD\ initial\ backward}, \tag{110}$$

$$DD_G = D_G \ \times s_G\ , \tag{111}$$

$$DD_{PM} = D_{PM} \ \times s_{PM}\ , \tag{112}$$

$$DD_{CBC}^{B_G} \ = D\ forward \times n_{CBC}^{B_G}, \tag{113}$$

$$DD_{NSC}^{B_G} \ = D\ forward \times n_{NSC}^{B_G}, \tag{114}$$

$$DD_{GCC}^{B_G} \ = D\ forward \times n_{GCC}^{B_G}, \tag{115}$$

$$DD_{CBC}^{A_{PM}} = D\ backward \times n_{CBC}^{A_{PM}}, \tag{116}$$

$$DD_{NSC}^{A_{PM}} = D\ backward \times n_{NSC}^{A_{PM}}, \tag{117}$$

$$DD_{GCC}^{A_{PM}} = D\ backward \times n_{GCC}^{A_{PM}}, \tag{118}$$

$$\begin{aligned} k\ DD\ production \\ &= (rate\ of\ accumulation\ of\ stationary\ nb_1\ NSCP \times D\ forward \\ &+ \ rate\ of\ accumulation\ of\ stationary\ na_2\ NSCP \times D\ backward) \\ &+ (rate\ of\ accumulation\ of\ stationary\ nb_1\ NSCD \times D\ forward \\ &+ rate\ of\ accumulation\ of\ stationary\ na_2\ NSCD\ \times D\ backward) \end{aligned} \tag{119}$$

$$C1_G = \frac{CC1_G}{s_G}, \tag{120}$$

$CBSPVA = \ cargo\ binding\ sites\ per\ unit\ vesicle\ area.$

$$sc1b1 = \frac{CBSPVA\ \times \frac{C1_G}{kc1b}}{\left(1 + \frac{C1_G}{kc1b}\right)}, \tag{121}$$

$$C1\ forward = \ sc1b1, \tag{122}$$

$$CC1\ final\ forward = \ C1\ forward\ \times final\ forward\ transport\ rate, \tag{123}$$

$$CC1\ initial\ backward = \ CC1\ final\ forward, \tag{124}$$

$$C1\ backward = \ \frac{CC1\ initial\ backward}{initial\ backtransport\ rate}, \tag{125}$$

$$C1_{PM} = \ \frac{kc1a \times \ CC1\ initial\ backward}{initial\ backward\ traport\ rate \times MBSPVA - CC1\ initial\ backward}, \tag{126}$$

$$CC1_{PM} = C1_{PM} \times s_{PM}, \tag{127}$$

$$CC1_{CBC}^{B_G} = C1\,forward \times n_{CBC}^{B_G}, \tag{128}$$

$$CC1_{NSC}^{B_G} = C1\,forward \times n_{NSC}^{B_G}, \tag{129}$$

$$CC1_{GCC}^{B_G} = C1\,forward \times n_{GCC}^{B_G}, \tag{130}$$

$$CC1_{CBC}^{A_{PM}} = C1\,backward \times n_{CBC}^{A_{PM}}, \tag{131}$$

$$CC1_{NSC}^{A_{PM}} = C1\,backward \times n_{NSC}^{A_{PM}}, \tag{132}$$

$$CC1_{GCC}^{A_{PM}} = C1\,backward \times n_{GCC}^{A_{PM}}, \tag{133}$$

$$\begin{aligned} k\,CC1\,production &= (rate\ of\ accumulation\ of\ stationary\ nb_1\ NSCP \times C1\,forward \\ &+ rate\ of\ accumulation\ of\ stationary\ na_2\ NSCP \times C1\,backward) \\ &+ (rate\ of\ accumulation\ of\ stationary\ nb_1\ NSCD \times C1\,forward \\ &+ rate\ of\ accumulation\ of\ stationary\ na_2\ NSCD \times C1\,backward) \end{aligned} \tag{134}$$

$$C2_{PM} = \frac{CC2_{PM}}{s_{PM}}, \tag{135}$$

$$sc2a2 = \frac{CBSPVA \times \frac{C2_{PM}}{kc2a}}{\left(1 + \frac{C2_{PM}}{kc2a}\right)}, \tag{136}$$

$$C2\,backward = sc2a2, \tag{137}$$

$$CC2\,initial\,backward = C2\,backward \times initial\,back\,transport\,rate, \tag{138}$$

$$CC2\,final\,forward = CC2\,initial\,backward, \tag{139}$$

$$C2\,forward = \frac{CC2\,final\,forward}{final\,forward\,transport\,rate}, \tag{140}$$

$$C2\,initial\,forkward = C2\,forward \times initial\,forward\,transport\,rate, \tag{141}$$

$$C2_G = \frac{kc2b \times CC2\,initial\,forward}{initial\,forward\,traport\,rate \times MBSPVA - CC2\,initial\,forward}, \tag{142}$$

$$CC2_G = C2_G \times s_G, \tag{143}$$

$$CC2_{CBC}^{B_G} = C2\,forward \times n_{CBC}^{B_G}, \tag{144}$$

$$CC2^{B_G}_{NSC} = C2\,forward \times n^{B_G}_{NSC}\,, \tag{145}$$

$$CC2^{B_G}_{GCC} = C2\,forward \times n^{B_G}_{GCC}\,, \tag{146}$$

$$CC2^{A_{PM}}_{CBC} = C2\,backward \times n^{A_{PM}}_{CBC}, \tag{147}$$

$$CC2^{A_{PM}}_{NSC} = C2\,backward \times n^{A_{PM}}_{NSC}, \tag{148}$$

$$CC2^{A_{PM}}_{GCC} = C2\,backward \times n^{A_{PM}}_{GCC}, \tag{149}$$

$$\begin{aligned} k\,CC2\,production &= (rate\ of\ accumulation\ of\ stationary\ nb_1\ NSCP \times C2\,forward \\ &+\ rate\ of\ accumulation\ of\ stationary\ na_2\ NSCP \times C2\,backward) \\ &+ (rate\ of\ accumulation\ of\ stationary\ nb_1\ NSCD \times C2\,forward \\ &+ rate\ of\ accumulation\ of\ stationary\ na_2\ NSCD \times C2\,backward) \end{aligned} \tag{150}$$

We have assumed a fixed effective tubulin concentration 9 µM and used Margolin model (Margolin et al., 2012) for simulations of dynamics microtubules for a range of hydrolysis rates (0.60 sec^{-1} – 0.80 sec^{-1}). To find the relationship between hydrolysis rate and average dynamic MT length, degradation rate of dynamic MT, axonal growth velocity. We assumed fixed Nucleation rate (65 $\min^{-1}$) and Stabilization rate (0.015 65 $\min^{-1}$) and used power fitting method to calculated fitting parameters values.

$$\left.\begin{aligned} a &= 0.7497 \\ b &= -0.07383 \\ c &= 0.091 \\ d &= -7.256 \\ e &= 13.35 \\ f &= 6.287 \end{aligned}\right\} \tag{151}$$

Consider an axonal growth velocity ‘v’ and hydrolysis rate ‘x’.

$$hydrolysis\ rate\ (x) = av^b \tag{152}$$

$$Average\ dyn\ MT\ length = cx^d \tag{153}$$

$$degradation\ rate = ex^f \tag{154}$$

$$\frac{d\ dyn_{MT}}{dt} = nucleation\ rate - (stabilization\ rate + degradation\ rate) \times dyn_{MT} \quad (155)$$

$$\frac{d\ stable_{MT\ length}}{dt} = \frac{stabilization\ rate \times dyn_{MT} \times Average\ dyn\ MT\ length}{Mts\ per\ crosssection} \quad (156)$$

$$dynamic_{MT\ length} = \frac{dyn_{MT} \times Average\ dyn\ MT\ length}{Mts\ per\ crosssection} \quad (157)$$

$$microtubule\ bundle\ length = dynamic_{MT\ length} + stable_{MT\ length} \quad (158)$$

Supp Table 1: Parameters and Initial Values This parameter set allows growth at 2 µm/h

Parameter	Value	Units	Reference
Number of microtubules per cross-section	20<br>(10 – 100)	#	(Fadic et al., 1985)
Diameter of neurite	1.0<br>(1.0 – 3.0)	µm	(Harris and Stevens, 1989)
Length of growth cones	10	µm	(Beller et al., 2013)
Kinesin motor protein anterograde velocity	1	µm/s	(Vale et al., 1996)<br>(Carter and Cross, 2005)
Dynein motor protein retrograde velocity	0.65	µm/s	(King and Schroer, 2000)<br>(Nishiura et al., 2004)
Vesicle diameter	39.7 ± 6.6	nm	(Zhang et al., 1998)
Growth cone surface area	70 - 200	μm^2	(Kunda et al., 2001)
Dissociation constant of SNARE SX with coat protein B. (k_{sx}^{A})	1.00	Molecules	Heinrich and Rapaport
Dissociation constant of SNARE SU with coat protein B. (k_{su}^{A})	1.00	Molecules	Heinrich and Rapaport
Dissociation constant of SNARE SX with coat protein B. (k_{sx}^{B})	10000	Molecules	Heinrich and Rapaport
Dissociation constant of SNARE SU with coat protein B. (k_{su}^{B})	100	Molecules	Heinrich and Rapaport
Dissociation constant of SNARE SY with coat protein B. (k_{sy}^{A})	10000	Molecules	Heinrich and Rapaport
Dissociation constant of SNARE SV with coat protein B. (k_{sv}^{A})	100	Molecules	Heinrich and Rapaport
Dissociation constant of SNARE SY with coat protein B. (k_{sy}^{B})	1.00	Molecules	Heinrich and Rapaport
Dissociation constant of SNARE SV with coat protein B. (k_{sv}^{B})	1.00	Molecules	Heinrich and Rapaport

Dissociation constants of recruitment factor 1 from coat protein A ($k_{r_1}^A$)	1.00	Molecules	Based on Henrich and Rappaport model
Dissociation constants of recruitment factor 1 from coat protein A ($k_{r_1}^B$)	10000	Molecules	Based on Henrich and Rappaport model
Dissociation constants of recruitment factor 2 from coat protein A ($k_{r_2}^A$)	10000	Molecules	Based on Henrich and Rappaport model
Dissociation constants of recruitment factor 2 from coat protein A ($k_{r_2}^B$)	1.00	Molecules	Based on Henrich and Rappaport model
Cargo binding spots per vesicle surface area	2	#	
Dissociation constants of motor kinesin kin_G from coat protein A (k_{kin}^A)	10	Molecules	Based on Henrich and Rappaport model
Dissociation constants of motor kinesin kin_G from coat protein B (k_{kin}^B)	0.1	Molecules	Based on Henrich and Rappaport model
Dissociation constants of motor Dynein Dyn_G from coat protein B (k_{dyn}^A)	0.1	Molecules	Based on Henrich and Rappaport model
Dissociation constants of motor Dynein Dyn_G from coat protein B (k_{dyn}^B)	10	Molecules	Based on Henrich and Rappaport model
Length of CBC compartment	1	µm	Assumed based on initial neurite length
Length of GCC compartment	2	µm	Assumed based on initial neurite length
Length of NSC compartment	20	µm	Assumed based on initial neurite length
Scale parameter multiplier (a_1)	3.146e-12		Predicted from (Margolin et al., 2012)
Scale parameter exponent (a_2)	1.0311		Predicted from (Margolin et al., 2012)
Scale parameter multiplier (b_1)	8.7342e-19		Predicted from (Margolin et al., 2012)
Scale parameter exponent (b_2)	4.2122		Predicted from (Margolin et al., 2012)
Shape multiplier (m)	0.02784		Predicted from (Margolin et al., 2012)
Shape constant (b)	0.15547		Predicted from (Margolin et al., 2012)
Degradation multiplier (d1)	-12.78		Predicted from (Margolin et al., 2012)
Degradation exponent (d2)	2.93e10		Predicted from (Margolin et al., 2012)
Anterograde vesicle surface area	0.05	μm^2	(Zhang et al., 1998)
Retrograde vesicle surface area	0.05	μm^2	(Zhang et al., 1998)
Snare binding spots per vesicle surface area	1400	#	Predicted

Motor binding spots per vesicle surface area	140	#	Predicted
SNARE Y production rate at TNG	3.4372	min^{-1}	Predicted
SNARE V production rate at TNG	0.74401	min^{-1}	Predicted
SNARE X production rate at TNG	9.4813	min^{-1}	Predicted
SNARE U production rate at TNG	774.5352	min^{-1}	Predicted
Motor K production rate at TNG	14.9856	min^{-1}	Predicted
Motor D production rate at TNG	12.3926	min^{-1}	Predicted
Recruitment factor 1 production rate at TNG	2.9971e-05	min^{-1}	Predicted
Recruitment factor 2 production rate at TNG	2.4785e-05	min^{-1}	Predicted
Vesicles budding rate at TGN with coat A (w_G^A)	1.0009e-05	µm^2	Predicted
Vesicles budding rate at Plasma Membrane with coat A (w_{PM}^A)	0.00010009	µm^2	Predicted
Vesicles budding rate at TGN with coat B (w_G^B)	0.00027089	µm^2	Predicted
Vesicles budding rate at Plasma Membrane with coat A (w_{PM}^B)	2.7078e-05	µm^2	Predicted
Tethering rate constant of vesicles (X, U) to target compartment Golgi (κ_{XU_G})	2e-06	1/ (molecules min)	Predicted
Tethering rate constant of vesicles (X, U) to target compartment Growth Cone Plasma Membrane ($\kappa_{XU_{PM}}$)	2e-07	1/ (molecules min)	Predicted
Tethering rate constant of vesicles (Y, V) to target compartment Golgi (κ_{YV_G})	5e-06	1/ (molecules min)	Predicted
Tethering rate constant of vesicles (Y, V) to target compartment Growth Cone Plasma Membrane ($\kappa_{YV_{PM}}$)	5e-05	1/ (molecules min)	Predicted
Required snare complex per vesicle fusion (R_{SCPVF})	5.00	#	Predicted
Fraction of bound kinesin motor with microtubules in CBC compartment	95	percent	Assumed based on (Dixit at al., 2008, LaPointe et al., 2009)
Fraction of bound kinesin motor with microtubules in NSC-P compartment	95	Percent	Assumed based on (Dixit at al., 2008, LaPointe et al., 2009)

Fraction of bound kinesin motor with microtubules in NSC-D compartment	0.022	Percent	Assumed based on (Dixit at al., 2008, LaPointe et al., 2009)
Fraction of bound kinesin motor with microtubules in GCC compartment	1.0	Percent	Assumed based on (Dixit at al., 2008, LaPointe et al., 2009)
Fraction of bound dynein motor with microtubules in CBC compartment	1.0	Percent	Assumed based on (Dixit at al., 2008, LaPointe et al., 2009)
Fraction of bound dynein motor with microtubules in NSC-P compartment	90	Percent	Assumed based on (Dixit at al., 2008, LaPointe et al., 2009)
Fraction of bound dynein motor with microtubules in NSC-D compartment	90	Percent	Assumed based on (Dixit at al., 2008, LaPointe et al., 2009)
Fraction of bound dynein motor with microtubules in CBC compartment	95	percent	Assumed based on (Dixit at al., 2008, LaPointe et al., 2009)
Nucleation rate of microtubules	65	1/min	Predicted from (Margolin et al., 2012)
Membrane production rate at TGN	0.8539	1/min	Predicted
Stabilization rate of dynamic microtubules	0.015	1/min	Predicted
Degradation rate of dynamic MTs	1.5819	1/min	Predicted from (Margolin et al., 2012)
**Initial Values**			
Trans Golgi Network (TGN)	50	μm^2	Predicted
Coat B budded vesicles membrane surface area from TGN in CBC	0.03	μm^2	Predicted
Coat B budded vesicles membrane surface area from TGN in NSC-P	0.10617	μm^2	Predicted
Coat B budded vesicles membrane surface area from NSC-P in NSC-D	449.0088	μm^2	Predicted
Coat B budded vesicles membrane surface area from TGN in GCC	60.5195	μm^2	Predicted
Growth cone plasma membrane	50	μm^2	Predicted
Coat A budded vesicles membrane surface area from TGN in CBC	2.2911e-06	μm^2	Predicted
Coat A budded vesicles membrane surface area from TGN in NSC	0.0008038	μm^2	Predicted

		μm^2	
Coat A budded vesicles membrane surface area from TGN in GCC	0.0067794		Predicted
Coat B budded vesicles membrane surface area from plasma membrane in CBC	0.01252	μm^2	Predicted
Coat B budded vesicles membrane surface area from plasma membrane in NSC-P	6.5014e-05	μm^2	Predicted
Coat B budded vesicles membrane surface area from plasma membrane in NSC-D	6.5014e-05	μm^2	Predicted
Coat B budded vesicles membrane surface area from plasma membrane in GCC	4.0126e-05	μm^2	Predicted
Coat A budded vesicles membrane surface area from plasma membrane in CBC	1.000	μm^2	Predicted
Coat A budded vesicles membrane surface area from plasma membrane in NSC-P	0.14245	μm^2	Predicted
Coat A budded vesicles membrane surface area from plasma membrane in NSC-D	0.14245	μm^2	Predicted
Coat A budded vesicles membrane surface area from plasma membrane in GCC	0.2	μm^2	Predicted
Neurite shaft surface area	62.8319	μm^2	Predicted
SNAREs Y in TGN compartment	5.7375	molecules	Predicted
Total vesicle snare Y with Coat B budded from TNG in CBC	0.13762	molecules	Predicted
Total vesicle snare Y with Coat B budded from TNG in NSC-P	0.48706	Molecules	Predicted
Total vesicle snare Y with Coat B budded from TNG in NSC-D	2059.7539	Molecules	Predicted
Total vesicle snare Y with Coat B budded from TNG in GCC	277.6231	Molecules	Predicted
SNAREs Y in GC compartment	20141.5989	Molecules	Predicted

Total vesicle snare Y with Coat A budded from TNG in CBC	1.9228e-11	Molecules	Predicted
Total vesicle snare Y with Coat A budded from TNG in NSC-P	6.746e-09	Molecules	Predicted
Total vesicle snare Y with Coat A budded from TNG in NSC-D	6.746e-09	Molecules	Predicted
Total vesicle snare Y with Coat A budded from TNG in GCC	0.00011986	Molecules	Predicted
Total vesicle snare Y with Coat B budded from GC in CBC	10.449	Molecules	Predicted
Total vesicle snare Y with Coat B budded from GC in NSC-P	0.054339	Molecules	Predicted
Total vesicle snare Y with Coat B budded from GC in NSC-D	0.054339	Molecules	Predicted
Total vesicle snare Y with Coat B budded from GC in GCC	0.033537	Molecules	Predicted
Total vesicle snare Y with Coat A budded from GC in CBC	5.5472	Molecules	Predicted
Total vesicle snare Y with Coat A budded from GC in NSC-P	0.7902	Molecules	Predicted
Total vesicle snare Y with Coat A budded from GC in NSC-D	0.7902	Molecules	Predicted
Total vesicle snare Y with Coat A budded from GC in GCC	1.1094	Molecules	Predicted
SNAREs V in TGN compartment	0.14	Molecules	Predicted
Total vesicle snare V with Coat B budded from TNG in CBC	0.029789	Molecules	Predicted
Total vesicle snare V with Coat B budded from TNG in NSC-P	0.10543	Molecules	Predicted
Total vesicle snare V with Coat B budded from TNG in NSC-D	445.8522	Molecules	Predicted

Total vesicle snare V with Coat B budded from TNG in GCC	60.094	Molecules	Predicted
SNAREs V in GC compartment	43.5983	Molecules	Predicted
Total vesicle snare V with Coat A budded from TNG in CBC	1.2923e-07	Molecules	Predicted
Total vesicle snare V with Coat A budded from TNG in NSC-P	4.5337e-05	molecules	Predicted
Total vesicle snare V with Coat A budded from TNG in NSC-D	4.5337e-05	molecules	Predicted
Total vesicle snare V with Coat A budded from TNG in GCC	0.00049928	Molecules	Predicted
Total vesicle snare V with Coat B budded from GC in CBC	7.0517	Molecules	Predicted
Total vesicle snare V with Coat B budded from GC in NSC-P	0.036543	Molecules	Predicted
Total vesicle snare V with Coat B budded from GC in NSC-D	0.036543	Molecules	Predicted
Total vesicle snare V with Coat B budded from GC in GCC	0.022554	Molecules	Predicted
Total vesicle snare V with Coat A budded from GC in CBC	1.2007	Molecules	Predicted
Total vesicle snare V with Coat A budded from GC in NSC-P	0.17105	Molecules	Predicted
Total vesicle snare V with Coat A budded from GC in NSC-D	0.17105	Molecules	Predicted
Total vesicle snare V with Coat A budded from GC in GCC	0.24015	Molecules	Predicted
SNAREs X in TGN compartment	19990.5187	Molecules	Predicted
Total vesicle snare X with Coat B budded from TNG in CBC	0.37962	Molecules	Predicted

Total vesicle snare X with Coat B budded from TNG in NSC-P	1.3435	Molecules	Predicted
Total vesicle snare X with Coat B budded from TNG in NSC-D	5681.6884	Molecules	Predicted
Total vesicle snare X with Coat B budded from TNG in GCC	765.8041	Molecules	Predicted
SNARE X in GC compartment	2.0142	Molecules	Predicted
Total vesicle snare X with Coat A budded from TNG in CBC	0.0019236	Molecules	Predicted
Total vesicle snare X with Coat A budded from TNG in NSC-P	0.67487	Molecules	Predicted
Total vesicle snare X with Coat A budded from TNG in NSC-D	0.67487	Molecules	Predicted
Total vesicle snare X with Coat A budded from TNG in GCC	5.6971	Molecules	Predicted
Total vesicle snare X with Coat B budded from GC in CBC	0.00048599	molecules	Predicted
Total vesicle snare X with Coat B budded from GC in NSC-P	5.4461e-10	Molecules	Predicted
Total vesicle snare X with Coat B budded from GC in NSC-D	5.4461e-10	Molecules	Predicted
Total vesicle snare X with Coat B budded from GC in GCC	3.3613e-10	Molecules	Predicted
Total vesicle snare X with Coat A budded from GC in CBC	15.3015	Molecules	Predicted
Total vesicle snare X with Coat A budded from GC in NSC-P	2.1797	Molecules	Predicted
Total vesicle snare X with Coat A budded from GC in NSC-D	2.1797	Molecules	Predicted

Total vesicle snare X with Coat A budded from GC in GCC	3.0603	Molecules	Predicted
SNAREs U in TGN compartment	15563.6828	Molecules	Predicted
Total vesicle snare U with Coat B budded from TNG in CBC	31.0112	Molecules	Predicted
Total vesicle snare U with Coat B budded from TNG in NSC-P	109.7533	Molecules	Predicted
Total vesicle snare U with Coat B budded from TNG in NSC-D	464143.3168	Molecules	Predicted
Total vesicle snare U with Coat B budded from TNG in GCC	62559.3683	Molecules	Predicted
SNARE U in GC compartment	453.8692	Molecules	Predicted
Total vesicle snare U with Coat A budded from TNG in CBC	0.001279	Molecules	Predicted
Total vesicle snare U with Coat A budded from TNG in NSC-P	0.44874	Molecules	Predicted
Total vesicle snare U with Coat A budded from TNG in NSC-D	0.44874	Molecules	Predicted
Total vesicle snare U with Coat A budded from TNG in GCC	3.7795	Molecules	Predicted
Total vesicle snare U with Coat B budded from GC in CBC	0.0011828	Molecules	Predicted
Total vesicle snare U with Coat B budded from GC in NSC-P	3.6171e-06	Molecules	Predicted
Total vesicle snare U with Coat B budded from GC in NSC-D	3.6171e-06	Molecules	Predicted
Total vesicle snare U with Coat B budded from GC in GCC	2.2324e-06	Molecules	Predicted
Total vesicle snare U with Coat A budded from GC in CBC	1250	Molecules	Predicted

Total vesicle snare U with Coat A budded from GC in NSC-P	178.0627	Molecules	Predicted
Total vesicle snare U with Coat A budded from GC in NSC-D	178.0627	Molecules	Predicted
Total vesicle snare U with Coat A budded from GC in GCC	250	Molecules	Predicted
Kinesin receptors K in TGN compartment	0.83333	Molecules	Predicted
Total kinesin receptors K with Coat B budded from TNG in CBC	0.6	Molecules	Predicted
Total kinesin receptors K with Coat B budded from TNG in NSC-P	2.1235	Molecules	Predicted
Total kinesin receptors K with Coat B budded from TNG in NSC-D	8980.1762	Molecules	Predicted
Total kinesin receptors K with Coat B budded from TNG in GCC	1210.3894	Molecules	Predicted
kinesin receptors K in GC compartment	104.4112	Molecules	Predicted
Total kinesin receptors K with Coat A budded from TNG in CBC	7.0251e-07	Molecules	Predicted
Total kinesin receptors K with Coat A budded from TNG in NSC-P	0.00024645	Molecules	Predicted
Total kinesin receptors K with Coat A budded from TNG in NSC-D	0.00024645	Molecules	Predicted
Total kinesin receptors K with Coat A budded from TNG in GCC	0.0020644	Molecules	Predicted
Total kinesin receptors K with Coat B budded from GC in CBC	1.6756	Molecules	Predicted
Total kinesin receptors K with Coat B budded from GC in NSC-P	0.0087054	Molecules	Predicted
Total kinesin receptors K with Coat B budded from GC in NSC-D	0.0087054	Molecules	Predicted

Total kinesin receptors K with Coat B budded from GC in GCC	0.0053728	Molecules	Predicted
Total kinesin receptors K with Coat A budded from GC in CBC	24.1848	Molecules	Predicted
Total kinesin receptors K with Coat A budded from GC in NSC-P	3.4451	Molecules	Predicted
Total kinesin receptors K with Coat A budded from GC in NSC-D	3.4451	Molecules	Predicted
Total kinesin receptors K with Coat A budded from GC in GCC	4.837	Molecules	Predicted
Dynein receptor D in TGN compartment	66.9821	Molecules	Predicted
Total dynein receptor D with Coat B budded from TNG in CBC	0.49618	Molecules	Predicted
Total dynein receptor D with Coat B budded from TNG in NSC-P	1.7561	Molecules	Predicted
Total dynein receptor D with Coat B budded from TNG in NSC-D	7426.2931	Molecules	Predicted
Total dynein receptor D with Coat B budded from TNG in GCC	1000.9499	Molecules	Predicted
Dynein receptor D in GC compartment	0.83333	Molecules	Predicted
Total dynein receptor D with Coat A budded from TNG in CBC	0.00030677	Molecules	Predicted
Total dynein receptor D with Coat A budded from TNG in NSC-P	0.10763	Molecules	Predicted
Total dynein receptor D with Coat A budded from TNG in NSC-D	0.10763	Molecules	Predicted
Total dynein receptor D with Coat A budded from TNG in GCC	0.90727	Molecules	Predicted
Total dynein receptor D with Coat B budded from GC in CBC	0.0038582	Molecules	Predicted

Total dynein receptor D with Coat B budded from GC in NSC-P	1.9925e-05	Molecules	Predicted
Total dynein receptor D with Coat B budded from GC in NSC-D	1.9925e-05	Molecules	Predicted
Total dynein receptor D with Coat B budded from GC in GCC	1.2297e-05	Molecules	Predicted
Total dynein receptor D with Coat A budded from GC in CBC	20	Molecules	Predicted
Total dynein receptor D with Coat A budded from GC in NSC-P	2.849	Molecules	Predicted
Total dynein receptor D with Coat A budded from GC in NSC-D	2.849	Molecules	Predicted
Total dynein receptor D with Coat A budded from GC in GCC	4	Molecules	Predicted
Recruitment factor 1 in TGN compartment	100	Molecules	Predicted
Total recruitment factor 1 with Coat B budded from TNG in CBC	1.2e-06	Molecules	Predicted
Total recruitment factor 1 with Coat B budded from TNG in NSC-P	4.2469e-06	Molecules	Predicted
Total recruitment factor 1 with Coat B budded from TNG in NSC-D	0.01796	Molecules	Predicted
Total recruitment factor 1 with Coat B budded from TNG in GCC	0.00242	Molecules	Predicted
recruitment factor 1 in GC compartment	0.0012092	Molecules	Predicted
Total recruitment factor 1 with Coat A budded from TNG in CBC	3.0544e-05	Molecules	Predicted
Total recruitment factor 1 with Coat A budded from TNG in NSC-P	0.010716	Molecules	Predicted
Total recruitment factor 1 with Coat A budded from TNG in NSC-D	0.010716	Molecules	Predicted

Total recruitment factor 1 with Coat A budded from TNG in GCC	0.090358	Molecules	Predicted
recruitment factor 1 in GC-PM compartment	0.0012092	Molecules	Predicted
Total recruitment factor 1 with Coat A budded from GC in CBC	4.8369e-05	Molecules	Predicted
Total recruitment factor 1 with Coat A budded from GC in NSC-P	6.8901e-06	Molecules	Predicted
Total recruitment factor 1 with Coat A budded from GC in NSC-D	6.8901e-06	Molecules	Predicted
Total recruitment factor 1 with Coat A budded from GC in GCC	9.6737e-06	Molecules	Predicted
Total recruitment factor 1 with Coat B budded from GC in CBC	7.4581e-11	Molecules	Predicted
Total recruitment factor 1 with Coat B budded from GC in NSC-P	3.6518e-13	Molecules	Predicted
Total recruitment factor 1 with Coat B budded from GC in NSC-D	3.6518e-13	Molecules	Predicted
Total recruitment factor 1 with Coat B budded from GC in GCC	2.2539e-13	Molecules	Predicted
Recruitment factor 2 in TGN compartment	0.00082696	Molecules	Predicted
Total recruitment factor 2 with Coat B budded from TNG in CBC	9.9234e-07	Molecules	Predicted
Total recruitment factor 2 with Coat B budded from TNG in NSC-P	3.512e-06	Molecules	Predicted
Total recruitment factor 2 with Coat B budded from TNG in NSC-D	0.014852	Molecules	Predicted
Total recruitment factor 2 with Coat B budded from TNG in GCC	0.0020019	Molecules	Predicted
Recruitment factor 2 in GC compartment	100	Molecules	Predicted

Total recruitment factor 2 with Coat A budded from TNG in CBC	1.1325e-14	Molecules	Predicted
Total recruitment factor 2 with Coat A budded from TNG in NSC-P	3.9661e-12	Molecules	Predicted
Total recruitment factor 2 with Coat A budded from TNG in NSC-D	3.9661e-12	Molecules	Predicted
Total recruitment factor 2 with Coat A budded from TNG in GCC	3.829e-11	Molecules	Predicted
Total recruitment factor 2 with Coat B budded from GC in CBC	0.16687	Molecules	Predicted
Total recruitment factor 2 with Coat B budded from GC in NSC-P	0.00086636	Molecules	Predicted
Total recruitment factor 2 with Coat B budded from GC in NSC-D	0.00086636	Molecules	Predicted
Total recruitment factor 2 with Coat B budded from GC in GCC	0.0005347	Molecules	Predicted
Total recruitment factor 2 with Coat A budded from GC in CBC	3.9999e-05	Molecules	Predicted
Total recruitment factor 2 with Coat A budded from GC in NSC-P	5.6979e-06	Molecules	Predicted
Total recruitment factor 2 with Coat A budded from GC in NSC-D	5.6979e-06	Molecules	Predicted
Total recruitment factor 2 with Coat A budded from GC in GCC	7.9998e-06	Molecules	Predicted
Effective tubulin	9	µM	Gardner et al., 2011
No of dynamic MTs in neurite shaft	20		Yu and Baas, 1994)
Initial total length of stable MTs length	15	µm	Assumed initial MTB length initial neurite length

For other velocities parameter values can be calculated using the equations described above. Matlab script for these calculations is available upon request.

Supplementary Figure - 10

A1

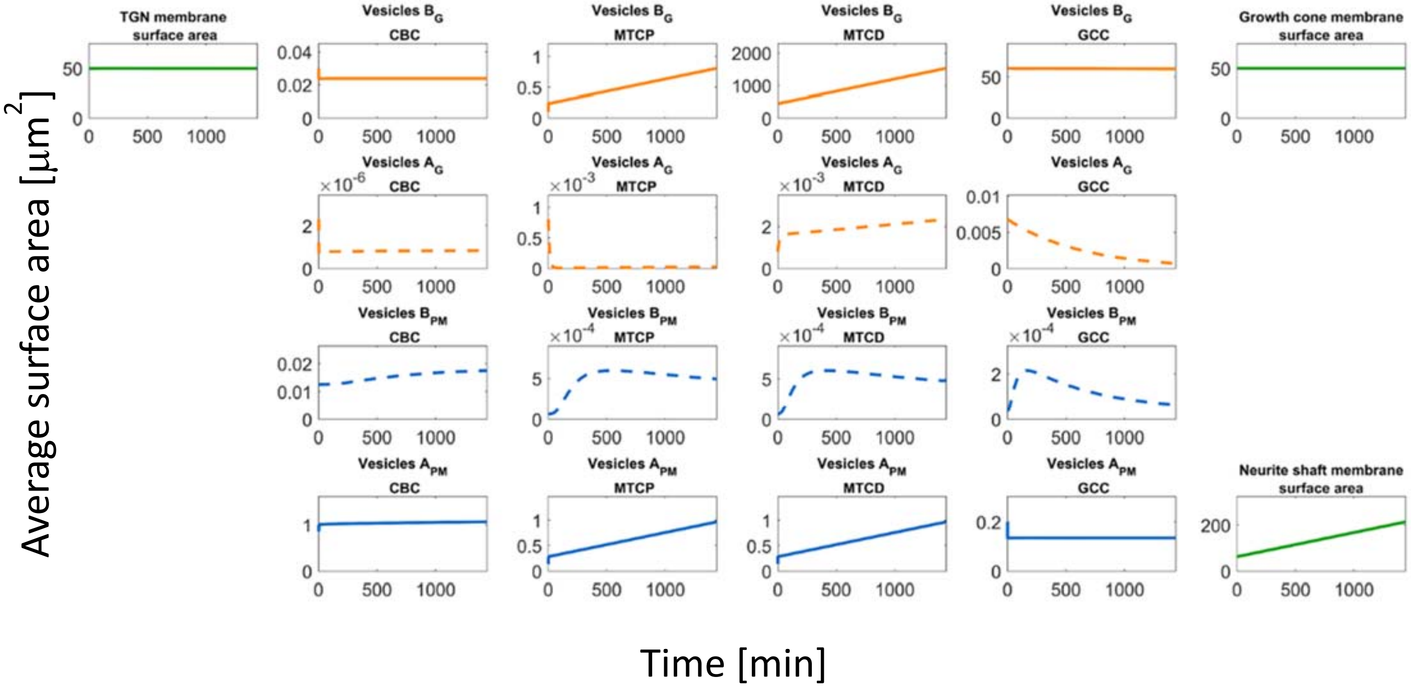

A2

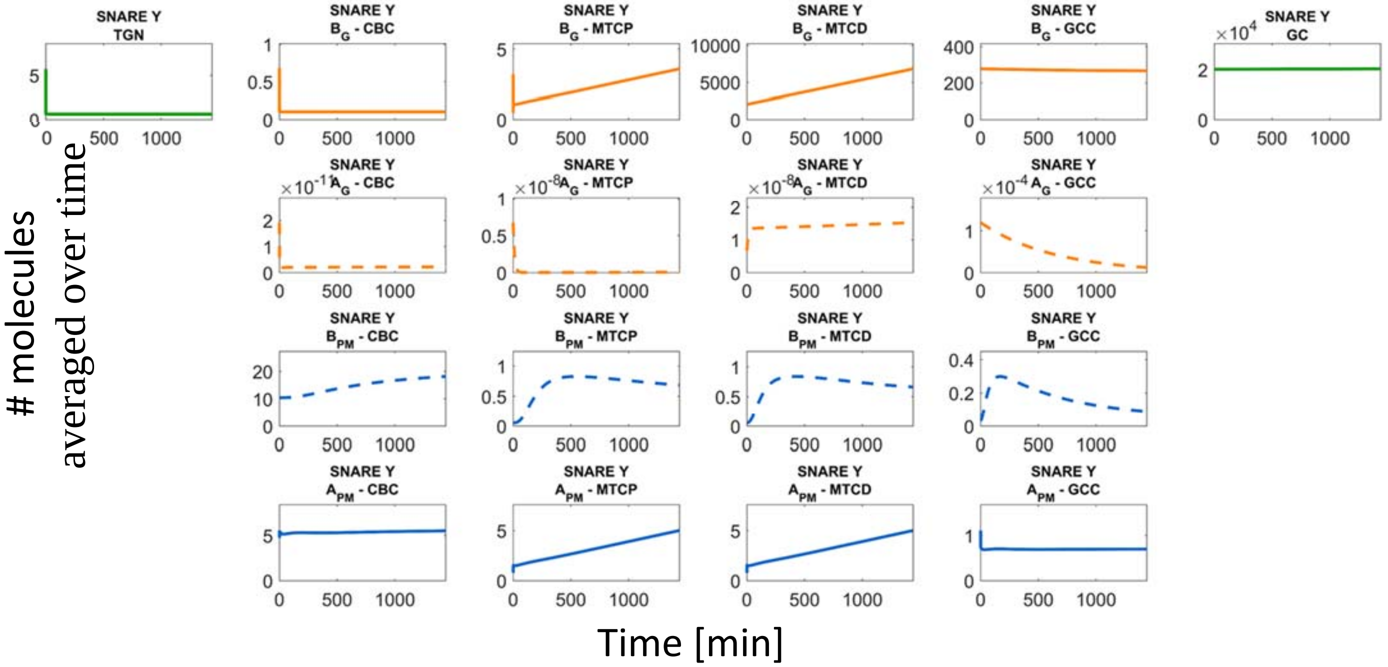

A3

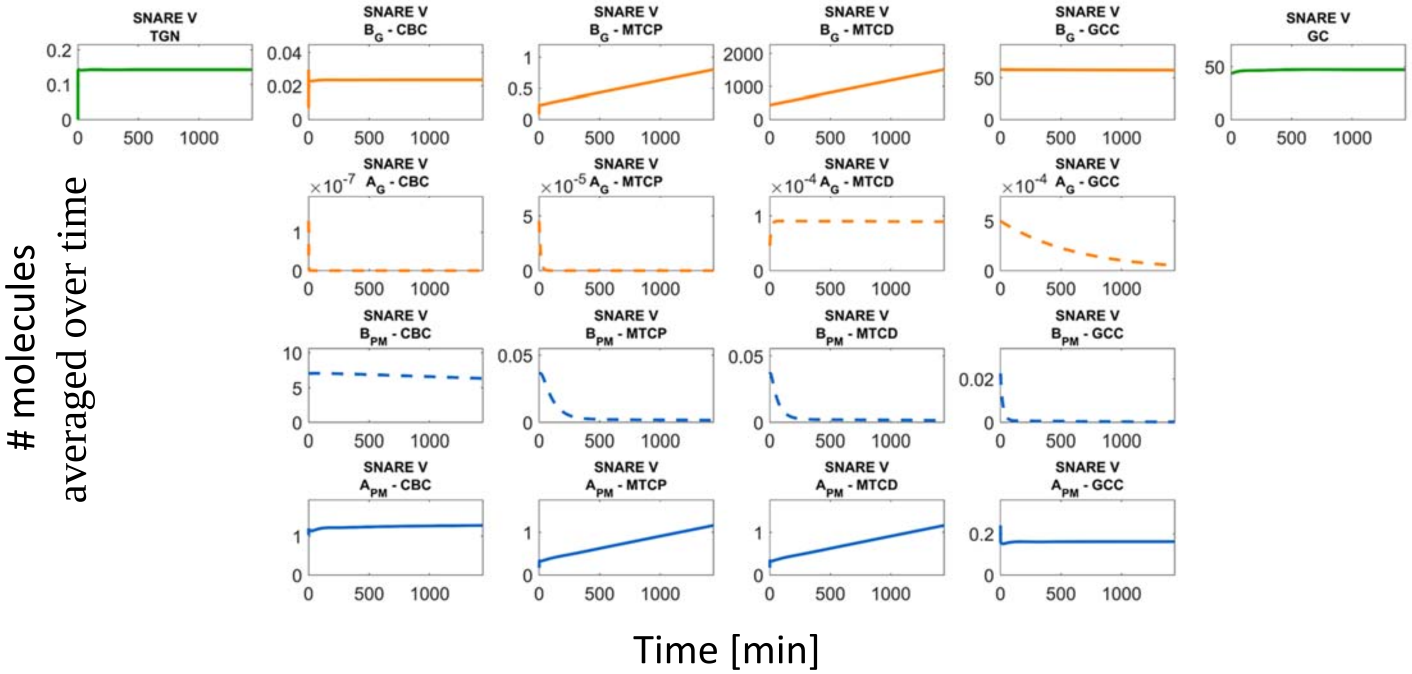
SNARE V TGN
SNARE V B_G - CBC
SNARE V B_G - MTCP
SNARE V B_G - MTCD
SNARE V B_G - GCC
SNARE V GC
SNARE V A_G - CBC
SNARE V A_G - MTCP
SNARE V A_G - MTCD
SNARE V A_G - GCC
SNARE V B_PM - CBC
SNARE V B_PM - MTCP
SNARE V B_PM - MTCD
SNARE V B_PM - GCC
SNARE V A_PM - CBC
SNARE V A_PM - MTCP
SNARE V A_PM - MTCD
SNARE V A_PM - GCC
molecules averaged over time
Time [min]

A4

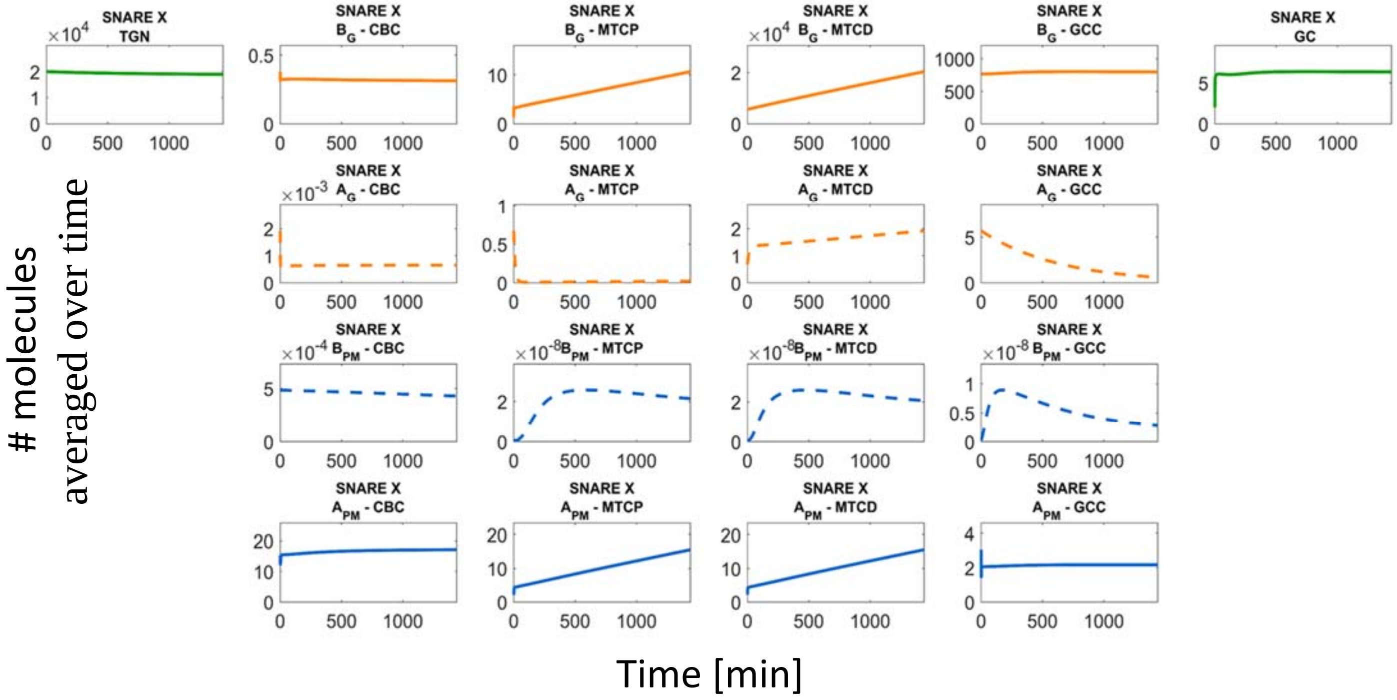
SNARE X TGN
SNARE X B_G - CBC
SNARE X B_G - MTCP
SNARE X B_G - MTCD
SNARE X B_G - GCC
SNARE X GC
SNARE X A_G - CBC
SNARE X A_G - MTCP
SNARE X A_G - MTCD
SNARE X A_G - GCC
SNARE X B_PM - CBC
SNARE X B_PM - MTCP
SNARE X B_PM - MTCD
SNARE X B_PM - GCC
SNARE X A_PM - CBC
SNARE X A_PM - MTCP
SNARE X A_PM - MTCD
SNARE X A_PM - GCC
molecules averaged over time
Time [min]

A5

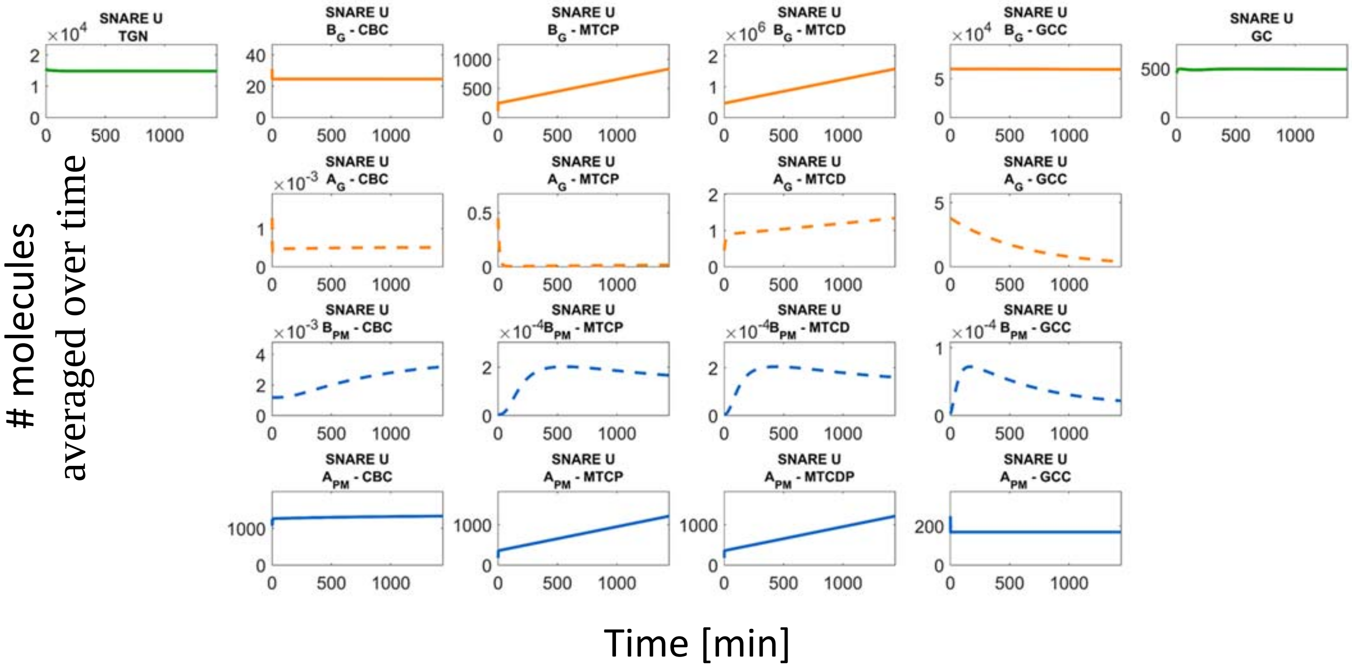
SNARE U TGN
SNARE U B_G - CBC
SNARE U B_G - MTCP
SNARE U B_G - MTCD
SNARE U B_G - GCC
SNARE U GC
SNARE U A_G - CBC
SNARE U A_G - MTCP
SNARE U A_G - MTCD
SNARE U A_G - GCC
SNARE U B_PM - CBC
SNARE U B_PM - MTCP
SNARE U B_PM - MTCD
SNARE U B_PM - GCC
SNARE U A_PM - CBC
SNARE U A_PM - MTCP
SNARE U A_PM - MTCDP
SNARE U A_PM - GCC
molecules averaged over time
Time [min]

A6

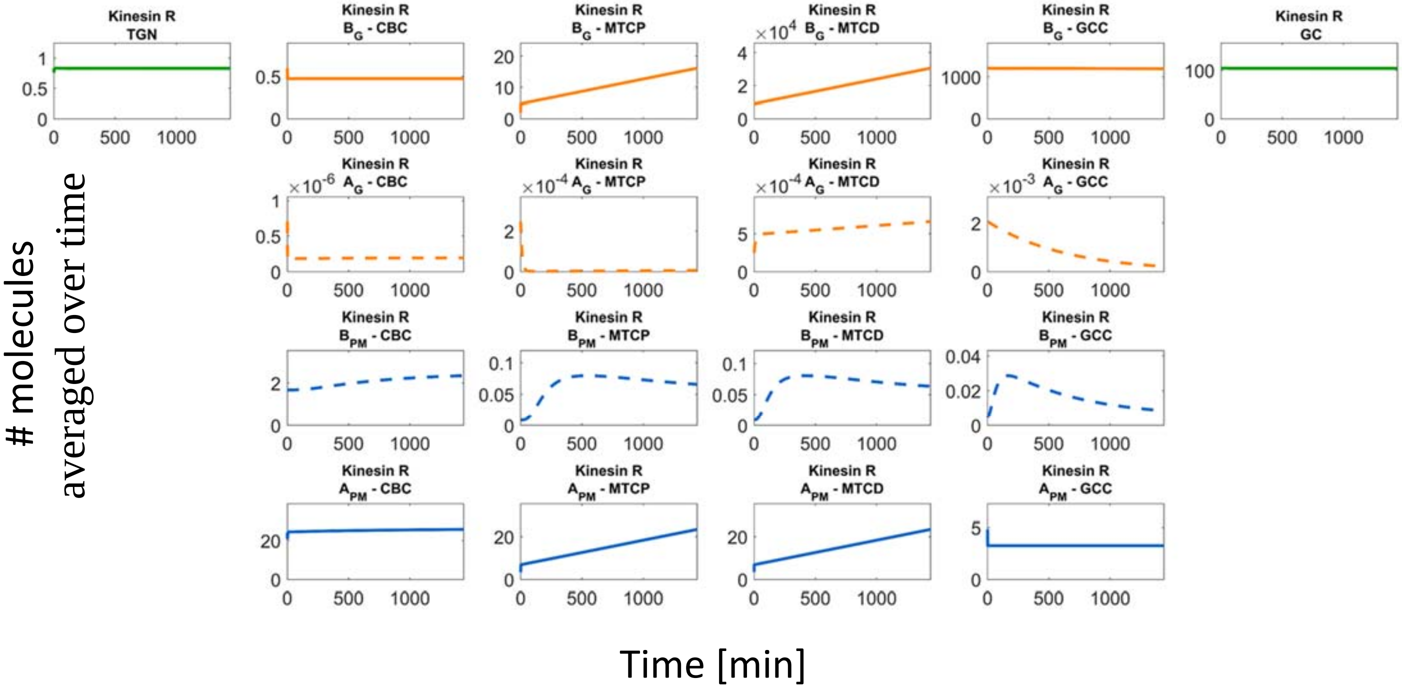
Kinesin R TGN
Kinesin R B_G - CBC
Kinesin R B_G - MTCP
Kinesin R B_G - MTCD
Kinesin R B_G - GCC
Kinesin R GC
Kinesin R A_G - CBC
Kinesin R A_G - MTCP
Kinesin R A_G - MTCD
Kinesin R A_G - GCC
Kinesin R B_PM - CBC
Kinesin R B_PM - MTCP
Kinesin R B_PM - MTCD
Kinesin R B_PM - GCC
Kinesin R A_PM - CBC
Kinesin R A_PM - MTCP
Kinesin R A_PM - MTCD
Kinesin R A_PM - GCC
molecules averaged over time
Time [min]

A7

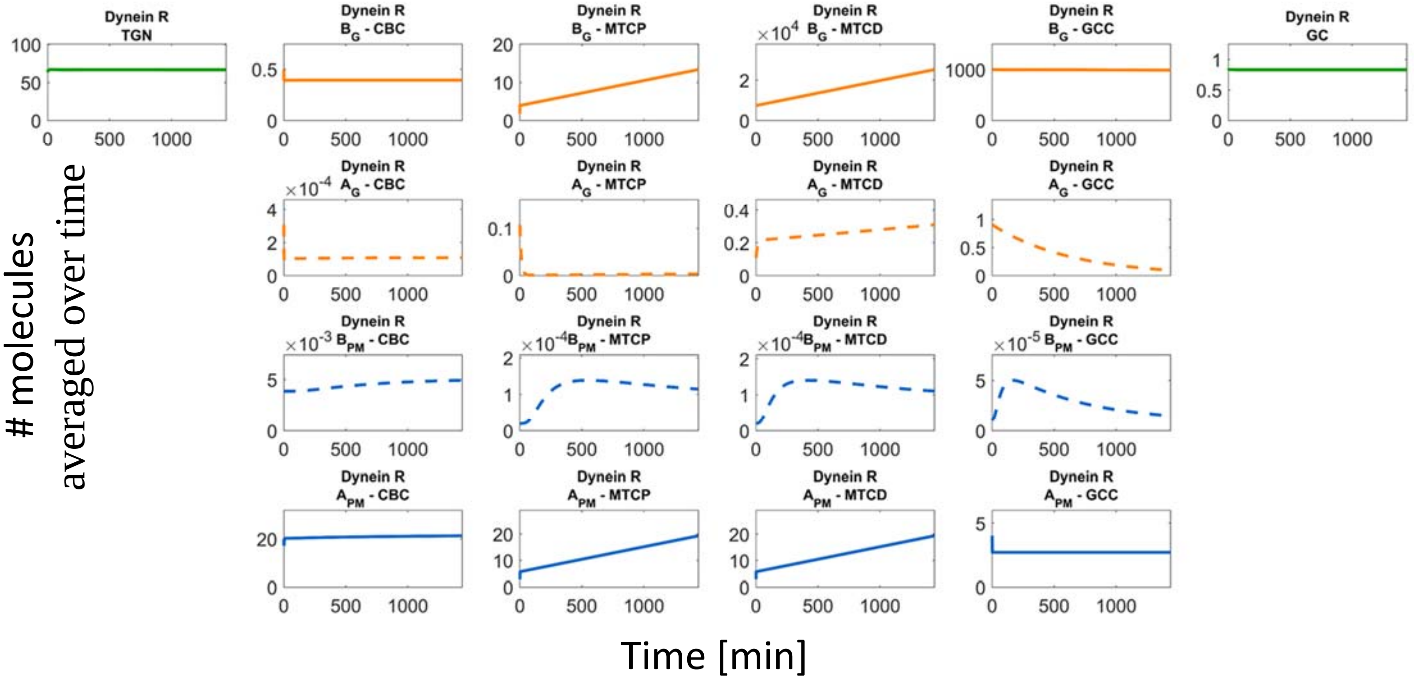

Dynein R TGN
Dynein R B_G - CBC
Dynein R B_G - MTCP
Dynein R B_G - MTCD
Dynein R B_G - GCC
Dynein R GC
Dynein R A_G - CBC
Dynein R A_G - MTCP
Dynein R A_G - MTCD
Dynein R A_G - GCC
Dynein R B_{PM} - CBC
Dynein R B_{PM} - MTCP
Dynein R B_{PM} - MTCD
Dynein R B_{PM} - GCC
Dynein R A_{PM} - CBC
Dynein R A_{PM} - MTCP
Dynein R A_{PM} - MTCD
Dynein R A_{PM} - GCC
molecules averaged over time
Time [min]

A8

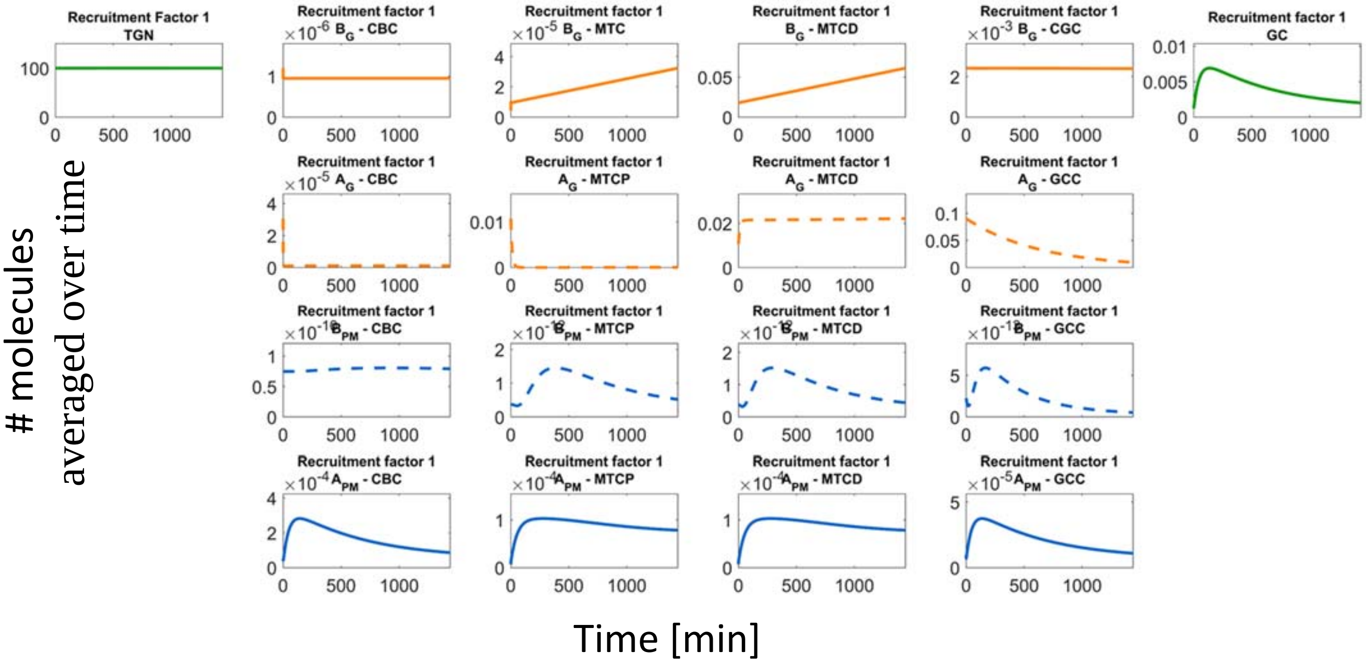

Recruitment Factor 1 TGN
Recruitment factor 1 B_G - CBC
Recruitment factor 1 B_G - MTC
Recruitment factor 1 B_G - MTCD
Recruitment factor 1 B_G - CGC
Recruitment factor 1 GC
Recruitment factor 1 A_G - CBC
Recruitment factor 1 A_G - MTCP
Recruitment factor 1 A_G - MTCD
Recruitment factor 1 A_G - GCC
Recruitment factor 1 B_{PM} - CBC
Recruitment factor 1 B_{PM} - MTCP
Recruitment factor 1 B_{PM} - MTCD
Recruitment factor 1 B_{PM} - GCC
Recruitment factor 1 A_{PM} - CBC
Recruitment factor 1 A_{PM} - MTCP
Recruitment factor 1 A_{PM} - MTCD
Recruitment factor 1 A_{PM} - GCC
molecules averaged over time
Time [min]

A9

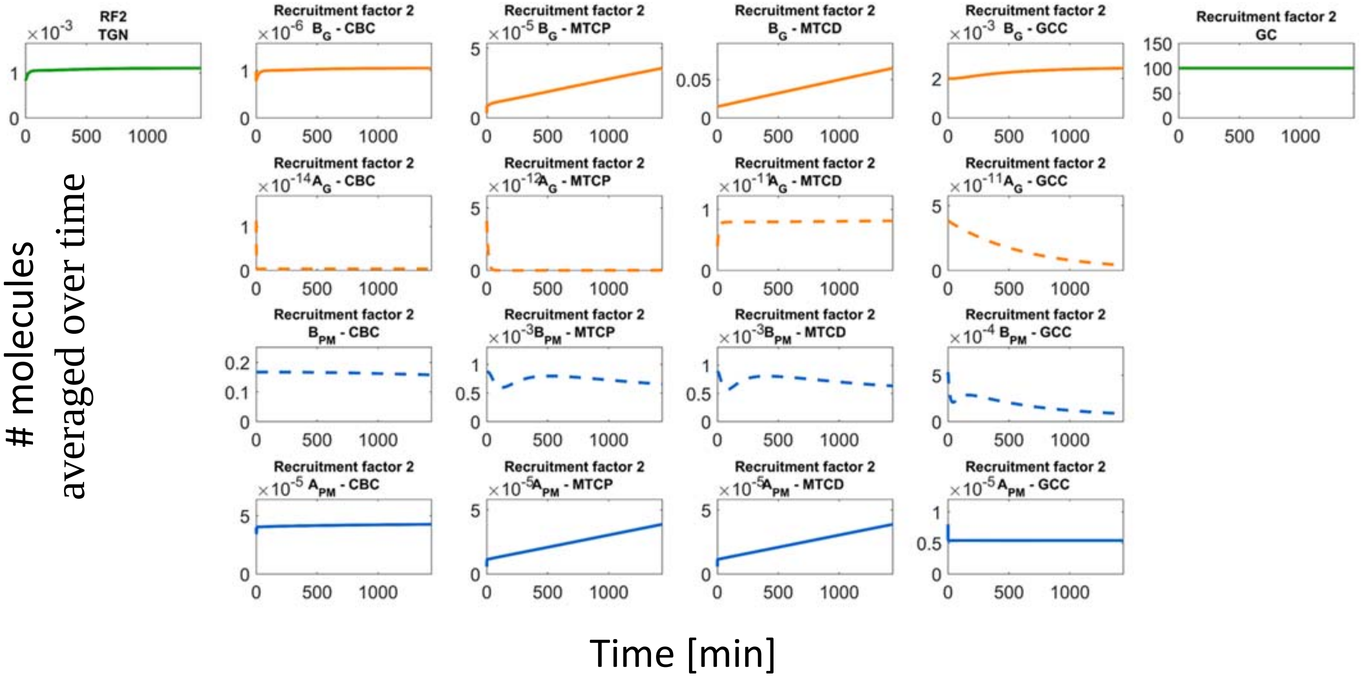

RF2 TGN
Recruitment factor 2 B_G - CBC
Recruitment factor 2 B_G - MTCP
Recruitment factor 2 B_G - MTCD
Recruitment factor 2 B_G - GCC
Recruitment factor 2 GC
Recruitment factor 2 A_G - CBC
Recruitment factor 2 A_G - MTCP
Recruitment factor 2 A_G - MTCD
Recruitment factor 2 A_G - GCC
Recruitment factor 2 B_PM - CBC
Recruitment factor 2 B_PM - MTCP
Recruitment factor 2 B_PM - MTCD
Recruitment factor 2 B_PM - GCC
Recruitment factor 2 A_PM - CBC
Recruitment factor 2 A_PM - MTCP
Recruitment factor 2 A_PM - MTCD
Recruitment factor 2 A_PM - GCC
molecules averaged over time
Time [min]

A10

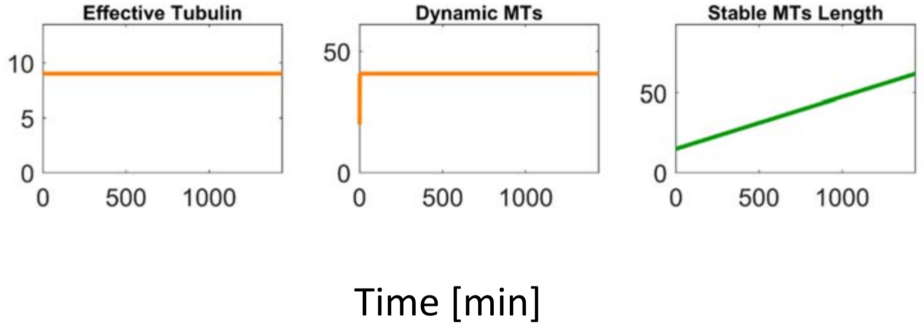

Effective Tubulin
Dynamic MTs
Stable MTs Length
Time [min]

B1

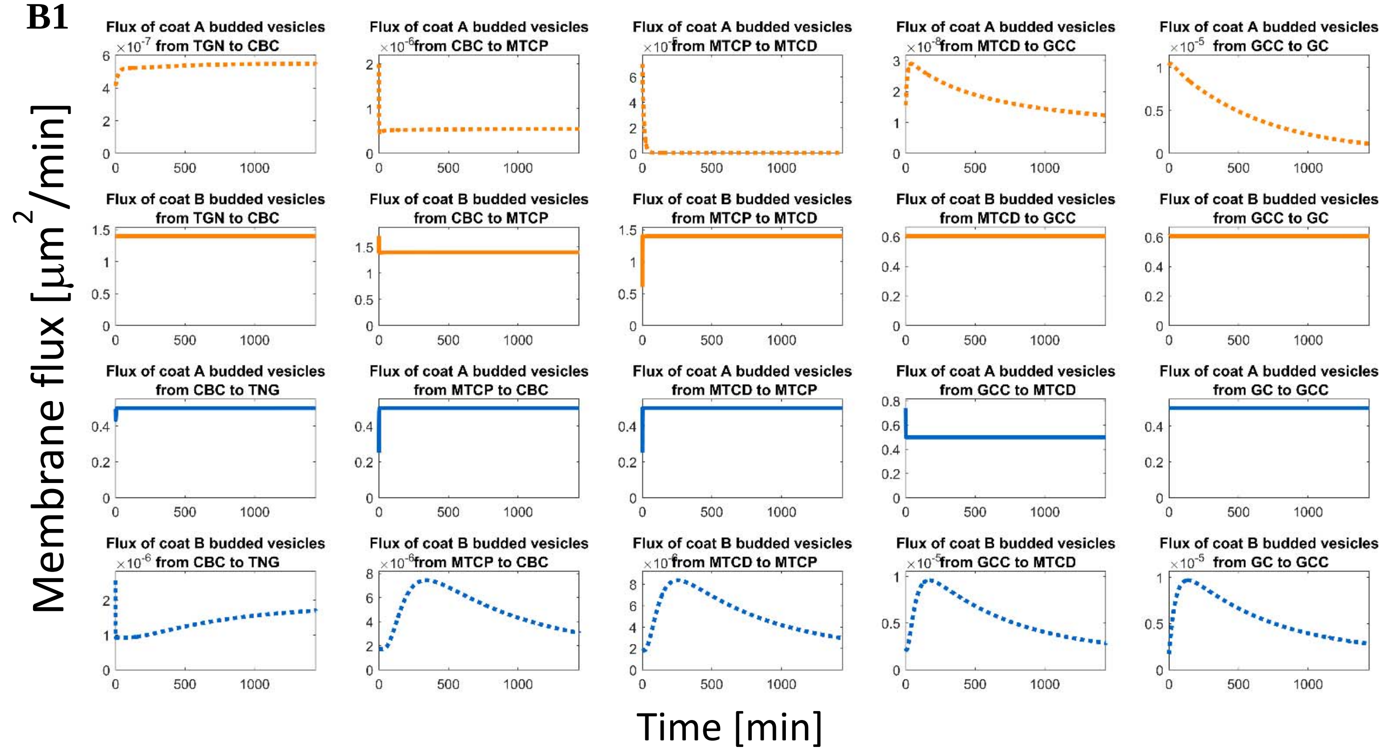

Membrane flux [μm^2/min]
Time [min]
Flux of coat A budded vesicles from TGN to CBC
Flux of coat A budded vesicles from CBC to MTCP
Flux of coat A budded vesicles from MTCP to MTCD
Flux of coat A budded vesicles from MTCD to GCC
Flux of coat A budded vesicles from GCC to GC
Flux of coat B budded vesicles from TGN to CBC
Flux of coat B budded vesicles from CBC to MTCP
Flux of coat B budded vesicles from MTCP to MTCD
Flux of coat B budded vesicles from MTCD to GCC
Flux of coat B budded vesicles from GCC to GC
Flux of coat A budded vesicles from CBC to TNG
Flux of coat A budded vesicles from MTCP to CBC
Flux of coat A budded vesicles from MTCD to MTCP
Flux of coat A budded vesicles from GCC to MTCD
Flux of coat A budded vesicles from GC to GCC
Flux of coat B budded vesicles from CBC to TNG
Flux of coat B budded vesicles from MTCP to CBC
Flux of coat B budded vesicles from MTCD to MTCP
Flux of coat B budded vesicles from GCC to MTCD
Flux of coat B budded vesicles from GC to GCC

B2

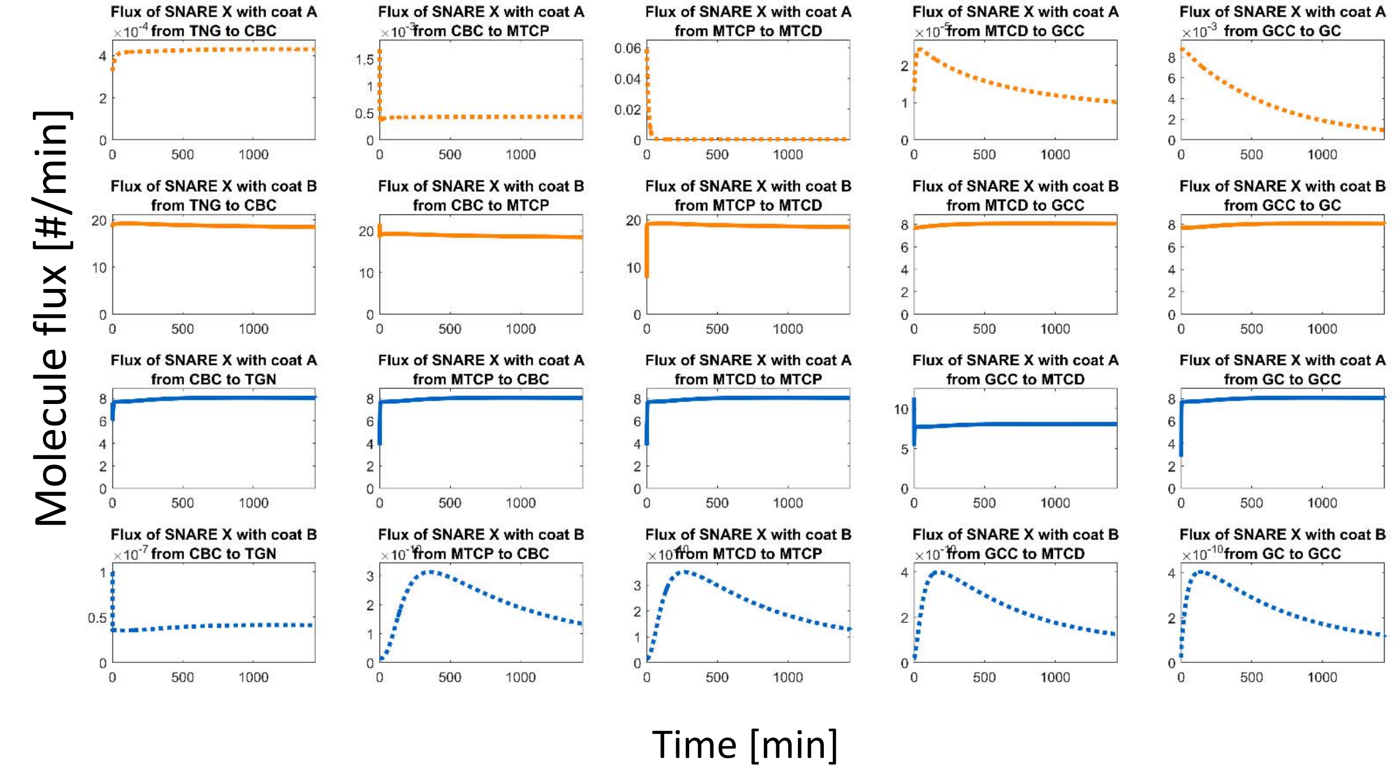

Molecule flux [#/min]
Time [min]
Flux of SNARE X with coat A from TNG to CBC
Flux of SNARE X with coat A from CBC to MTCP
Flux of SNARE X with coat A from MTCP to MTCD
Flux of SNARE X with coat A from MTCD to GCC
Flux of SNARE X with coat A from GCC to GC
Flux of SNARE X with coat B from TNG to CBC
Flux of SNARE X with coat B from CBC to MTCP
Flux of SNARE X with coat B from MTCP to MTCD
Flux of SNARE X with coat B from MTCD to GCC
Flux of SNARE X with coat B from GCC to GC
Flux of SNARE X with coat A from CBC to TGN
Flux of SNARE X with coat A from MTCP to CBC
Flux of SNARE X with coat A from MTCD to MTCP
Flux of SNARE X with coat A from GCC to MTCD
Flux of SNARE X with coat A from GC to GCC
Flux of SNARE X with coat B from CBC to TGN
Flux of SNARE X with coat B from MTCP to CBC
Flux of SNARE X with coat B from MTCD to MTCP
Flux of SNARE X with coat B from GCC to MTCD
Flux of SNARE X with coat B from GC to GCC

B3

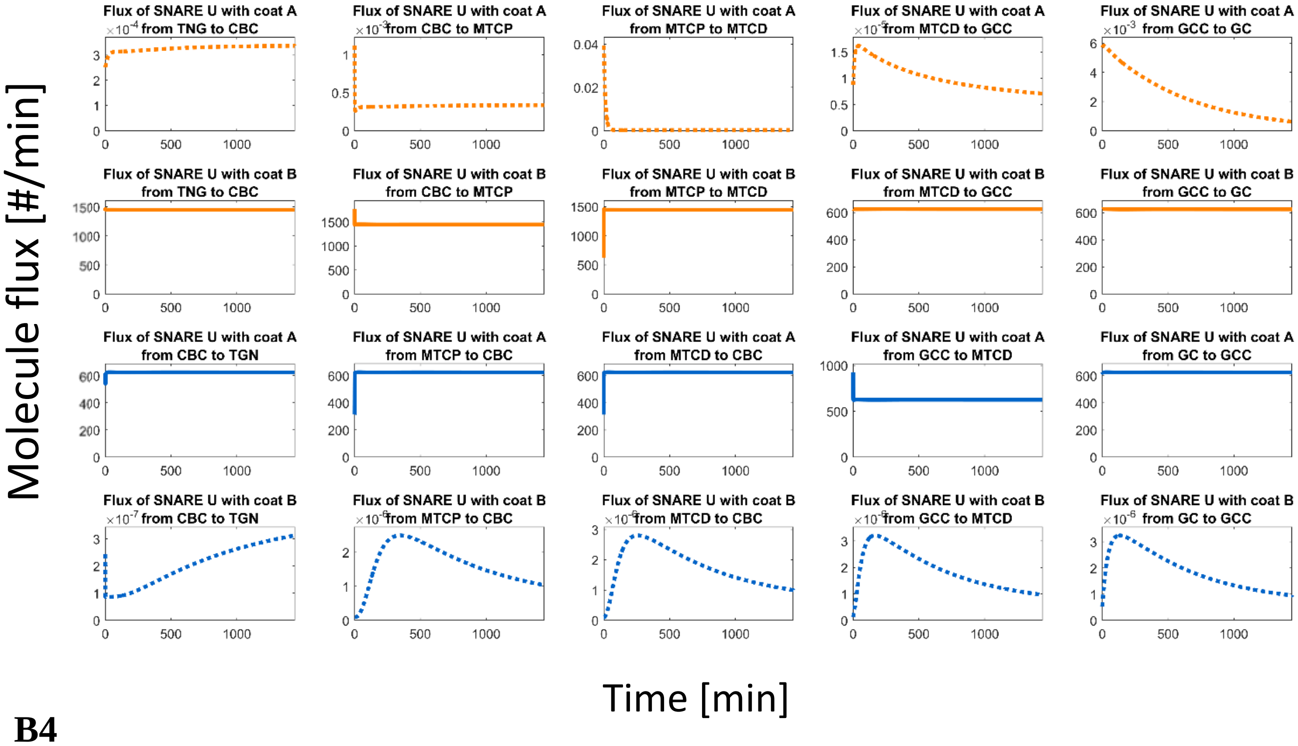
Flux of SNARE U with coat A from TNG to CBC
Flux of SNARE U with coat A from CBC to MTCP
Flux of SNARE U with coat A from MTCP to MTCD
Flux of SNARE U with coat A from MTCD to GCC
Flux of SNARE U with coat A from GCC to GC
Flux of SNARE U with coat B from TNG to CBC
Flux of SNARE U with coat B from CBC to MTCP
Flux of SNARE U with coat B from MTCP to MTCD
Flux of SNARE U with coat B from MTCD to GCC
Flux of SNARE U with coat B from GCC to GC
Flux of SNARE U with coat A from CBC to TGN
Flux of SNARE U with coat A from MTCP to CBC
Flux of SNARE U with coat A from MTCD to CBC
Flux of SNARE U with coat A from GCC to MTCD
Flux of SNARE U with coat A from GC to GCC
Flux of SNARE U with coat B from CBC to TGN
Flux of SNARE U with coat B from MTCP to CBC
Flux of SNARE U with coat B from MTCD to CBC
Flux of SNARE U with coat B from GCC to MTCD
Flux of SNARE U with coat B from GC to GCC
Molecule flux [#/min]
Time [min]

B4

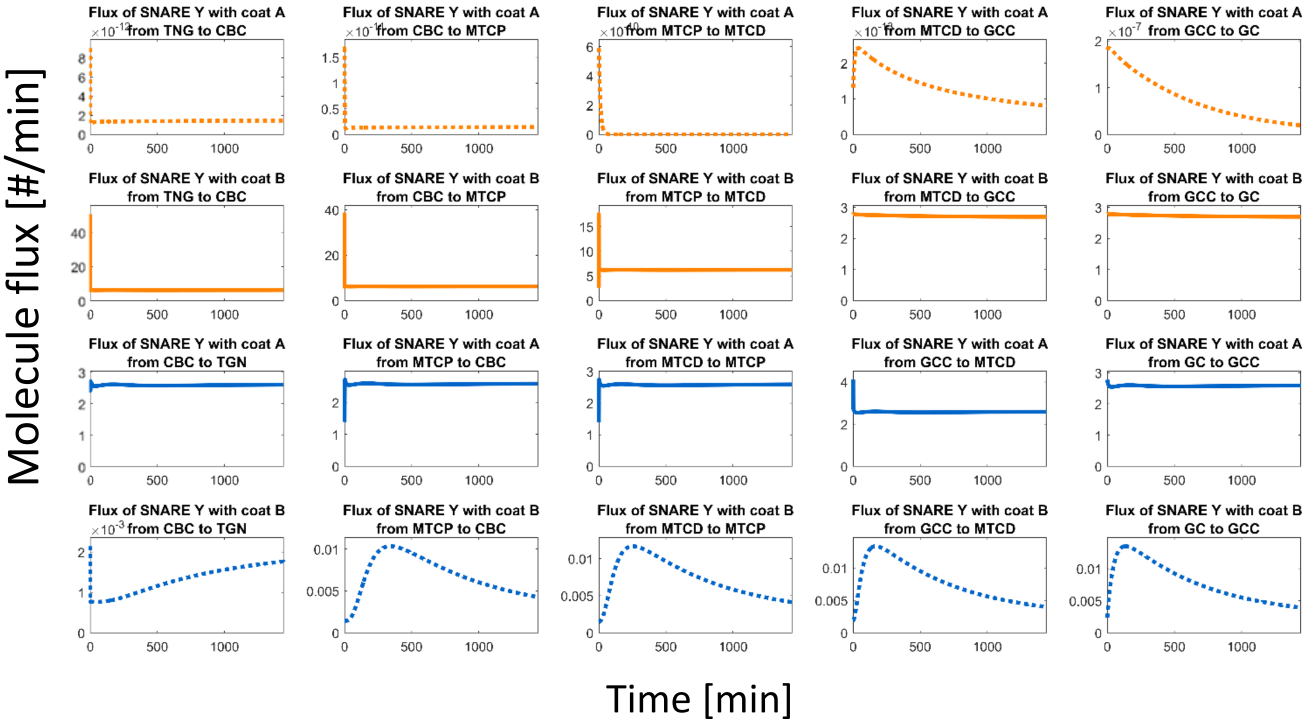
Flux of SNARE Y with coat A from TNG to CBC
Flux of SNARE Y with coat A from CBC to MTCP
Flux of SNARE Y with coat A from MTCP to MTCD
Flux of SNARE Y with coat A from MTCD to GCC
Flux of SNARE Y with coat A from GCC to GC
Flux of SNARE Y with coat B from TNG to CBC
Flux of SNARE Y with coat B from CBC to MTCP
Flux of SNARE Y with coat B from MTCP to MTCD
Flux of SNARE Y with coat B from MTCD to GCC
Flux of SNARE Y with coat B from GCC to GC
Flux of SNARE Y with coat A from CBC to TGN
Flux of SNARE Y with coat A from MTCP to CBC
Flux of SNARE Y with coat A from MTCD to MTCP
Flux of SNARE Y with coat A from GCC to MTCD
Flux of SNARE Y with coat A from GC to GCC
Flux of SNARE Y with coat B from CBC to TGN
Flux of SNARE Y with coat B from MTCP to CBC
Flux of SNARE Y with coat B from MTCD to MTCP
Flux of SNARE Y with coat B from GCC to MTCD
Flux of SNARE Y with coat B from GC to GCC
Molecule flux [#/min]
Time [min]

B5

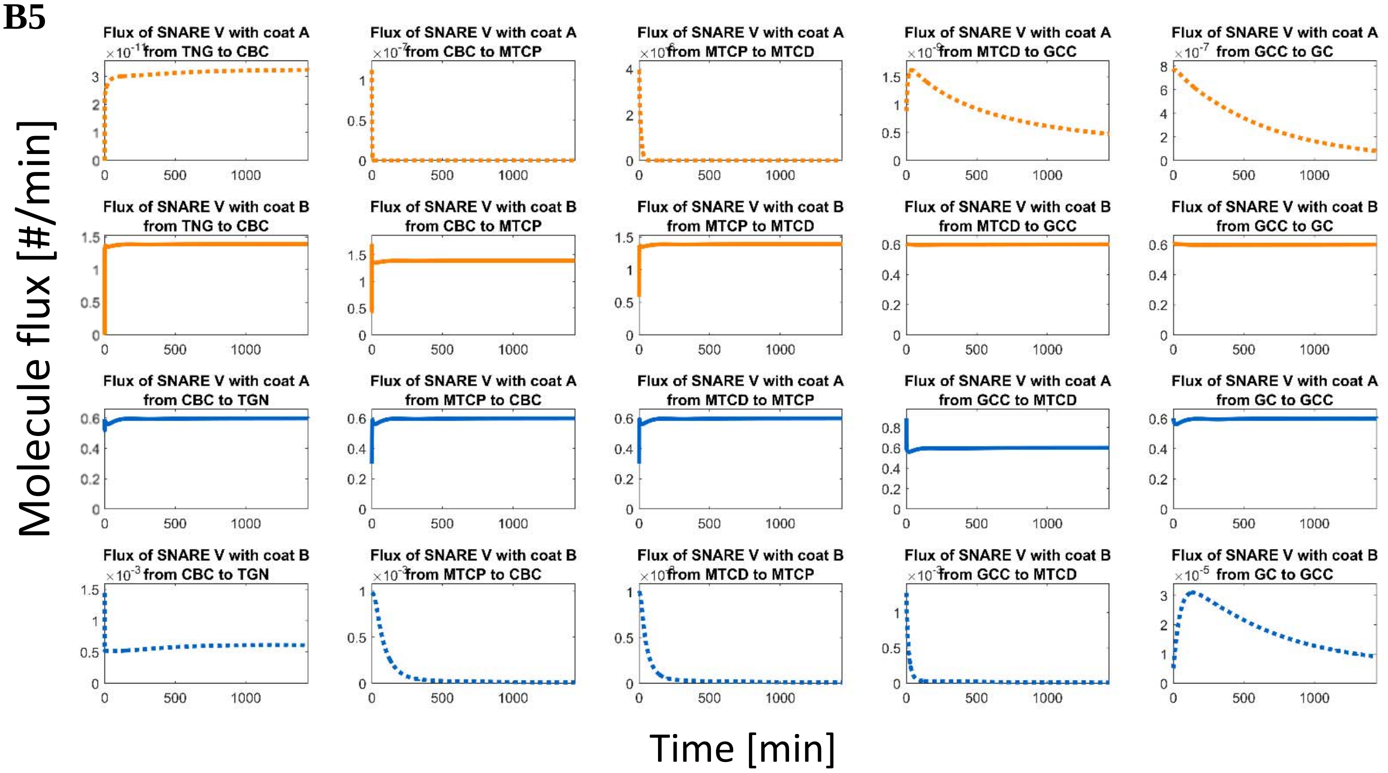
Flux of SNARE V with coat A from TNG to CBC
Flux of SNARE V with coat A from CBC to MTCP
Flux of SNARE V with coat A from MTCP to MTCD
Flux of SNARE V with coat A from MTCD to GCC
Flux of SNARE V with coat A from GCC to GC
Flux of SNARE V with coat B from TNG to CBC
Flux of SNARE V with coat B from CBC to MTCP
Flux of SNARE V with coat B from MTCP to MTCD
Flux of SNARE V with coat B from MTCD to GCC
Flux of SNARE V with coat B from GCC to GC
Flux of SNARE V with coat A from CBC to TGN
Flux of SNARE V with coat A from MTCP to CBC
Flux of SNARE V with coat A from MTCD to MTCP
Flux of SNARE V with coat A from GCC to MTCD
Flux of SNARE V with coat A from GC to GCC
Flux of SNARE V with coat B from CBC to TGN
Flux of SNARE V with coat B from MTCP to CBC
Flux of SNARE V with coat B from MTCD to MTCP
Flux of SNARE V with coat B from GCC to MTCD
Flux of SNARE V with coat B from GC to GCC
Molecule flux [#/min]
Time [min]

B6

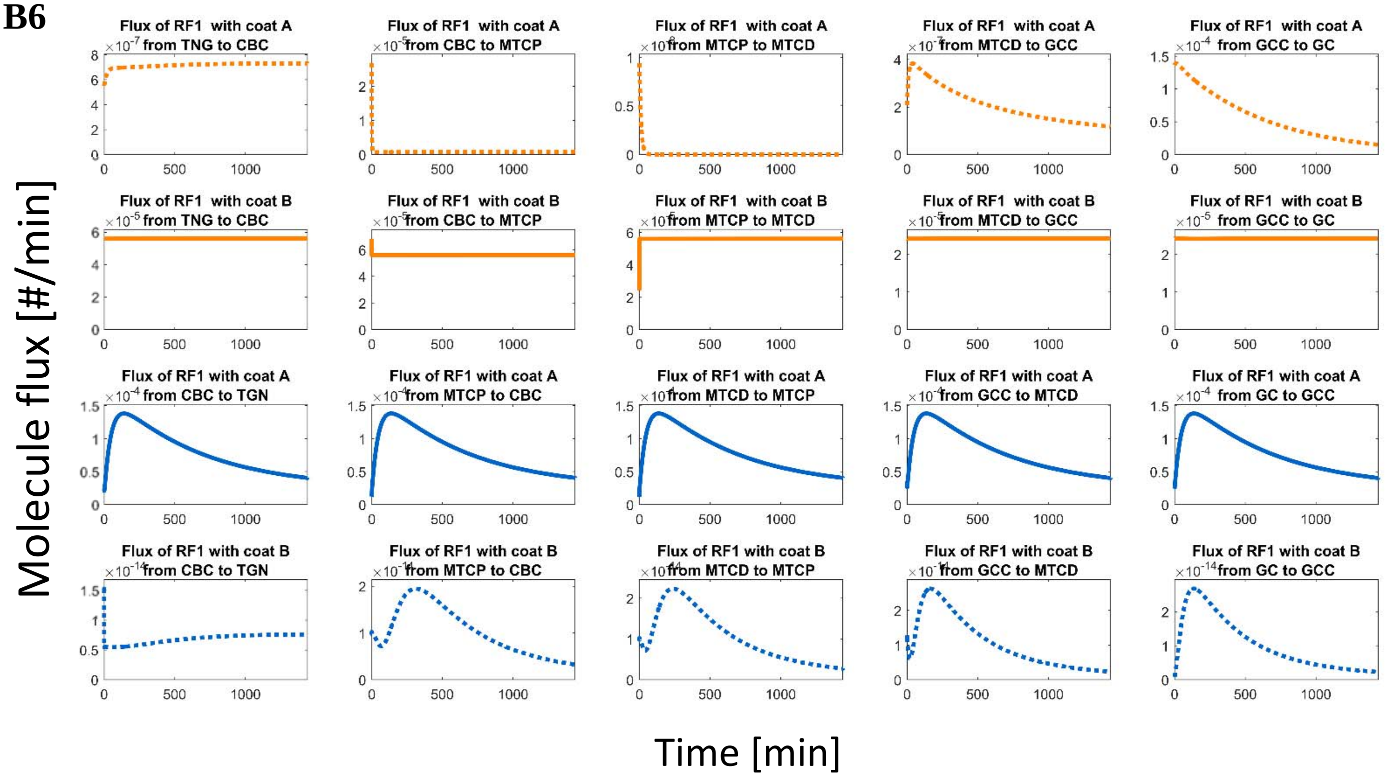
Flux of RF1 with coat A from TNG to CBC
Flux of RF1 with coat A from CBC to MTCP
Flux of RF1 with coat A from MTCP to MTCD
Flux of RF1 with coat A from MTCD to GCC
Flux of RF1 with coat A from GCC to GC
Flux of RF1 with coat B from TNG to CBC
Flux of RF1 with coat B from CBC to MTCP
Flux of RF1 with coat B from MTCP to MTCD
Flux of RF1 with coat B from MTCD to GCC
Flux of RF1 with coat B from GCC to GC
Flux of RF1 with coat A from CBC to TGN
Flux of RF1 with coat A from MTCP to CBC
Flux of RF1 with coat A from MTCD to MTCP
Flux of RF1 with coat A from GCC to MTCD
Flux of RF1 with coat A from GC to GCC
Flux of RF1 with coat B from CBC to TGN
Flux of RF1 with coat B from MTCP to CBC
Flux of RF1 with coat B from MTCD to MTCP
Flux of RF1 with coat B from GCC to MTCD
Flux of RF1 with coat B from GC to GCC
Molecule flux [#/min]
Time [min]

B7

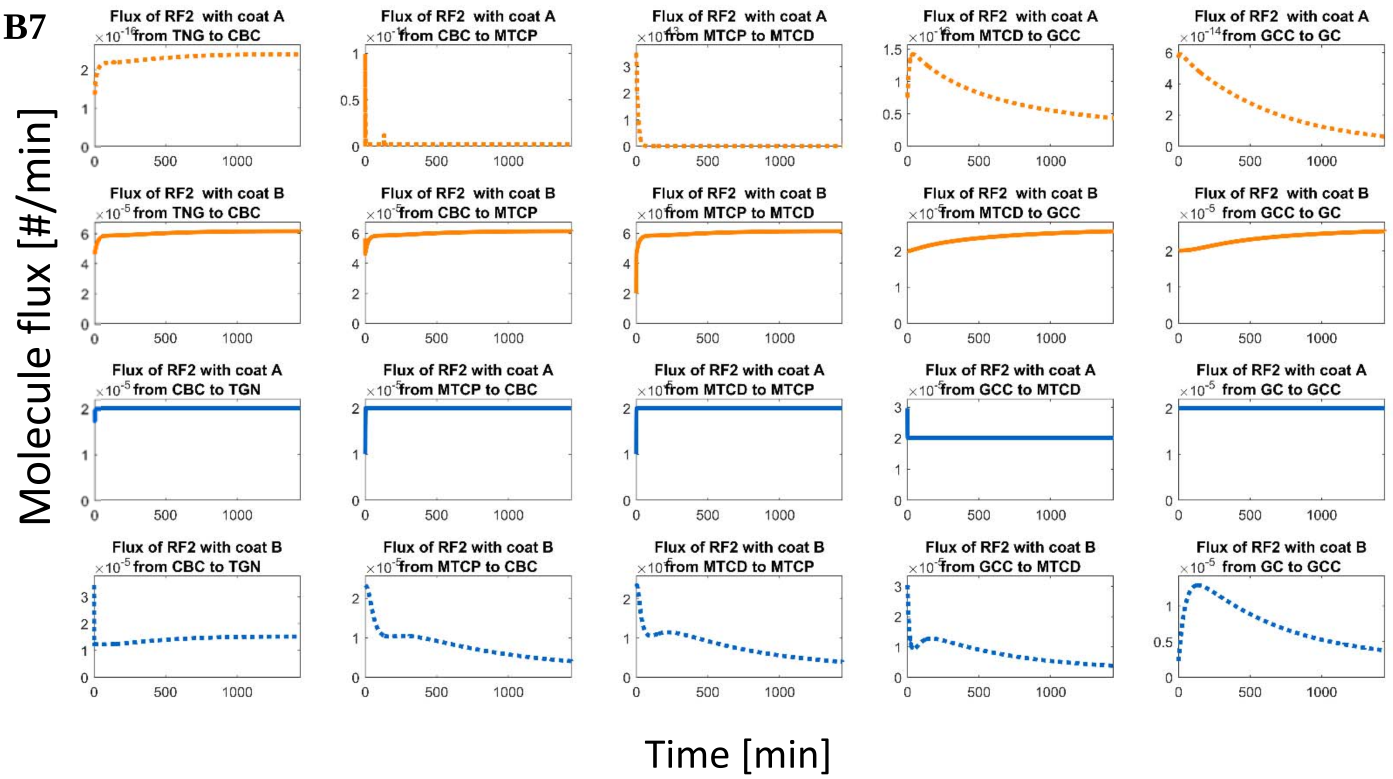

Supplementary Figure 10: Steady state distribution and fluxes of proteins and membrane vesicles. (**A)** At steady state all proteins and membrane amounts stay constant in the different compartments, except in the neurite shaft cytoplasm (NSC-P & NSC-D) and the neurite shaft. Neurite shaft growth is facilitated by continuous membrane addition, so the membrane surface area of the neurite shaft grows over the time. The NSC-D grows parallel to the neurite shaft and acts as a sink for transport vesicles, thereby accumulating vesicle membrane and vesicle membrane proteins. The figures show the membrane surface areas or protein amounts in each compartment within each set of vesicles. B_G refers to anterograde vesicles that bud from the TGN with the coat protein B, A_G refers to anterograde vesicles that bud from the TGN with the coat A, B_{PM} refers to retrograde vesicles that bud from the GC-PM with coat protein B and A_{PM} to retrograde vesicles that bud from the GC with coat protein A. Green lines refer to TGN or growth cone plasma membrane (GC-PM), orange lines refer to anterograde moving vesicles (B_G, A_G) and blue lines to retrograde moving vesicles (B_{PM}, A_{PM}). Solid lines represent those vesicles that are mainly responsible for membrane transport in the indicated direction (i.e. B_G in the anterograde direction and A_{PM} in the retrograde direction). Figure 3(A1-A9) shows distribution of anterograde and retrograde (vesicles, SNAREs-Y, SNAREs-V, SNAREs-X, SNAREs-U, Kinesin receptor-k, Dynein receptor-d, Recruitment factor-1, Recruitment factor-2) and A10 shows effective tubulin concentration of the model, number of dynamic microtubules and dynamics of stable microtubules. **(B)** Figure (B1-B11) shows all fluxes, i.e. membrane or protein transfers from one compartment into the other are shown. Colors and line styles are the same as in (A).

Materials

Antibodies

**Name**	**Company**	**Cat. Number**
Purified anti-tubulin Beta 3 (TUBB3) mouse	Biolegend	801202
STAT3 (124H6) mouse	Cell Signaling	9139S
phospho YB1 (S102) (C34A2) Rabbit	Cell Signaling	2900S
p35/25 (C64810) Rabbit	Cell Signaling	2680S
Anti Albumin Chicken	Sigma	SAB3500217
phospho STAT3 (TYR705) (D3A7) Rabbit	Cell Signaling	9145S
Alexa Fluor 488 mouse	Invitrogen	A11029
Alexa Fluor 568 mouse	Invitrogen	A11031
Anti Cholera Toxin B Subunit Goat	List Biological Lab	703
GAP-43 Antibody	Novus Biologicals	NB300-143

Media and Supplements

**Name**	**Company**	**Cat. Number**
Neurobasal	Thermo Fisher Scientific	2103-049
Pen-Strep	Thermo Fisher Scientific	15140-122
Feta bovine serum	Thermo Fisher Scientific	10439-026
L-Glutamine	Thermo Fisher Scientific	25030-081
B27 Supplement	Thermo Fisher Scientific	17504044
Papain from papaya extract	Sigma	P5306
Deoxyribonuclease I from bovine pancreas	Sigma	DN25
Poly-L-lysine hydrobromide	Sigma	P1274
OptiPrep	Sigma	D1556

Fixing and Staining

Name	Company	Cat. Number
Triton X 100	Sigma	X100
Phosphate Buffer Saline (10X)	Boston Bioproducts	BM-220
Normal Donkey Serum	Jackson Immuno Research	017-000-121
Tetrahydrofuran	Sigma	401757
Sucrose	Sigma	S7905
Paraformyldehyde 16% solution	Electron Microscopy Science	15710-S
Hoeschst 33342	Invitrogen	953557
Mountant- Permafluor	Thermo Fisher Scientific	TA-030-FM
OCT Compound	Sakura Tissue Tek	4583
Corning Cover Glass	Corning	2980-245
50 mm Glass Bottom Dishes	Mattek Corp.	P50G-1.5-30-F
Standard neuron device	XONA	SND150

Western Blot

Name	Company	Cat. Number
SDS 4X Buffer	Boston Bioproducts	BP-110R
Albumin Standard 2mg/ml	Thermo Fisher Scientific	23210
BioRad Protein Assay	BioRad	5000006
mini-protean tgx precast gels	BioRad	456-1094
30% Acrylimade/Bis Solution	BioRad	160158
Odyssey Blocking Buffer (TBS)	Li-COR	927-500000
Halt Protease Phosphatase Inhibitors	Thermo Fisher Scientific	78443
IRDye® Goat anti-Rabbit 800CW Secondary	Li-COR	925-32211
IRDye® Goat anti-Mouse 680RD Secondary	Li-COR	925-68070
IRDye® 800CW Donkey anti-Chicken	Li-COR	925-32218
Tris-buffered saline (10X)	Boston Bioproducts	BM-300
Tris-glycine SDS Running Buffer (10X)	Boston Bioproducts	BP-150
TWEEN® 20	Sigma	P9416
Goat anti-Rabbit IgG (H+L) Secondary Antibody, HRP	Thermo Fisher Scientific	31460
Goat anti-Mouse IgG (H+L) Secondary Antibody, HRP	Thermo Fisher Scientific	31430
PMSF (Phenylmethylsulfonyl fluoride)	Sigma	10837091001
Pepstatin	Sigma	10253286001

RIPA Buffer (10X)	Cell Signaling	9806
Ammonium Persulfate	Thermo Fisher Scientific	17874
Sodium dodecyl sulfate	Sigma	L3771
Methanol	Sigma	179337
Transfer Buffer (10X)	Boston Bioproducts	BP-190
Calcein, AM	Thermo Fisher Scientific	C3200MP

Drugs

**Name**	**Company**	**Cat. Number**
Human Activated Protein C	Haematologic Technologies	HCAPC-0080
HU-210	SIGMA	H7909
Interleukin-6 from rat, recombinant	SIGMA	I0406
Paclitaxel (Taxol)	SIGMA	T7402
Dimethyl Sulphide, sterile	SIGMA	D2650

Surgeries

**Name**	**Company**	**Cat. Number**
Forane Isoflurane	Baxter	10019-360-40
Buprenorphine HCL	ParPharmaceutical	42023-179-01
Vetasan Ointment	Kinetic Vet	9006-06-00
Alcohol Swabs	Becton Dickinson	08290-3268-95-326895
Vetbond	3M	14695B
Nanofil Syringe	World Precision Instruments	nanofil
Neomycin ointment	Bausch + Lomb	24208-780-55
Ketamin HCL	Vedco Inc	50989-161-06
AnaSed Xylazine	AKORN	59399-111-50
